# Atlas of phenotypic, genotypic and geographical diversity present in the European traditional tomato

**DOI:** 10.1093/hr/uhac112

**Published:** 2022-05-17

**Authors:** Clara Pons, Joan Casals, Samuela Palombieri, Lilian Fontanet, Alessandro Riccini, Jose Luis Rambla, Alessandra Ruggiero, Maria del Rosario Figás, Mariola Plazas, Athanasios Koukounaras, Maurizio E Picarella, Maria Sulli, Josef Fisher, Peio Ziarsolo, Jose Blanca, Joaquin Cañizares, Maria Cammareri, Antonella Vitiello, Giorgia Batelli, Angelos Kanellis, Matthijs Brouwer, Richard Finkers, Konstantinos Nikoloudis, Salvador Soler, Giovanni Giuliano, Stephania Grillo, Silvana Grandillo, Dani Zamir, Andrea Mazzucato, Mathilde Causse, Maria José Díez, Jaime Prohens, Antonio Jose Monforte, Antonio Granell

**Affiliations:** 1 Instituto de Conservación y Mejora de la Agrodiversidad Valenciana (COMAV), Universitat Politècnica de València, València, Spain; 2 Instituto de Biología Molecular y Celular de Plantas (IBMCP). Consejo Superior de Investigaciones Científicas (CSIC), Universitat Politècnica de València, València, Spain; 3 Department of Agri-Food Engineering and Biotechnology/Miquel Agustí Foundation, Universitat Politècnica de Catalunya, Campus Baix Llobregat, Esteve Terrades 8, 08860 Castelldefels, Spain; 4 Institute of Biosciences and BioResources (IBBR), National Research Council of Italy (CNR), Via Università 133, 80055 Portici, Italy; 5 INRAE, UR1052, Génétique et Amélioration des Fruits et Légumes 67 Allé des Chênes, Centre de Recherche PACA, Domaine Saint Maurice, CS60094, Montfavet, 84143, France; 6 Department of Agriculture and Forest Sciences (DAFNE), Università degli Studi della Tuscia, Viterbo,Italy; 7 Aristotle University of Thessaloniki, School of Agriculture, Laboratory of Vegetable Crops, Thessaloniki, 54124 Greece; 8 Italian National Agency for New Technologies, Energy and Sustainable Economic Development (ENEA), Casaccia Research Centre, Rome, Italy; 9 Hebrew University of Jerusalem, Robert H Smith Inst Plant Sci & Genet Agr, Rehovot, Israel; 10 Group of Biotechnology of Pharmaceutical Plants, Laboratory of Pharmacognosy, Department of Pharmaceutical Sciences, Aristotle University of Thessaloniki, 54124 Thessaloniki, Greece; 11 Wageningen Univ & Res, Plant Breeding, POB 386, NL-6700 AJ Wageningen, Netherlands; 12 Agroindustrial Cooperative of Tympaki, 70200 Tympaki, Greece; 13 HM Clause, Portes-lès-Valence, France; 14 Department of Agriculture and Forest Sciences (DAFNE), University of Tuscia, 01100 Viterbo, Italy

## Abstract

The Mediterranean basin countries are considered secondary centres of tomato diversification. However, information on phenotypic and allelic variation of local tomato materials is still limited. Here we report on the evaluation of the largest traditional tomato collection, which includes 1499 accessions from Southern Europe. Analyses of 70 traits revealed a broad range of phenotypic variability with different distributions among countries, with the culinary end use within each country being the main driver of tomato diversification. Furthermore, eight main tomato types (phenoclusters) were defined by integrating phenotypic data, country of origin, and end use. Genome-wide association study (GWAS) meta-analyses identified associations in 211 loci, 159 of which were novel. The multidimensional integration of phenoclusters and the GWAS meta-analysis identified the molecular signatures for each traditional tomato type and indicated that signatures originated from differential combinations of loci, which in some cases converged in the same tomato phenotype. Our results provide a roadmap for studying and exploiting this untapped tomato diversity.

## Introduction

Tomato diversity is the result of the long-term interaction between humans and the *Solanum* sect. *Lycopersicon* species, through the anthropogenic selection pressure exercised progressively on the genetic variability present in the closest wild relatives of the crop during domestication, the diversification during tomato cultivation history, and more recently, introgressions from wild relatives by modern plant breeding activities. Since its arrival to Europe (beginning of the 16th century), the tomato was rapidly adopted into Spanish and Italian diets [1, 2] and later, since the 18th century, into the kitchens of the rest of the European countries, and afterwards, to the rest of the world [3]. The 500 years of cultivation and selection have resulted in a plethora of varieties that are firmly rooted in Southern Europe, which are likely the result of farmer-driven selection for adaptation to local environments and growing conditions and to fit the tastes of the local population. These varieties can be enclosed into the three major different tomato classes [4–7] based on their main end use: fresh market (FM), processing (PR) and long shelf-life (LSL). These groups are defined by specific characteristics: FM and PR tomatoes have a short ripening time and rapidly decay after harvest, with FM tomatoes consumed fresh (salads), and PR tomatoes cooked and/or canned. The LSL tomatoes show a long postharvest shelf life (between 4 and 6 months) and are consumed after preservation or aging, cooked, or spread on bread.

Nowadays, traditional European cultivars are not simple redundant original American landraces [8]. Traditional European cultivars display an impressive variability in fruit characteristics that are unique to this gene pool. This is clearly exemplified in mutations such as *sun* [9] or *alcobaça* (*alc*, [10]), which produce long fruits, or LSL cultivars, respectively, which originated in Mediterranean countries. Therefore, Southern Europe is considered a secondary centre of tomato diversification [8, 11, 12]. Moreover, the higher genetic diversity found in Spanish and Italian accessions as compared with other Southern European regions suggests that those regions might be independent secondary centres of diversity with a different history [12]. In addition, farmers introduced additional variability into the traditional pool (“traditionalization”) [12] from varieties developed by breeding companies since the 18^th^ to 21^st^ centuries [1].

**Figure 1:**
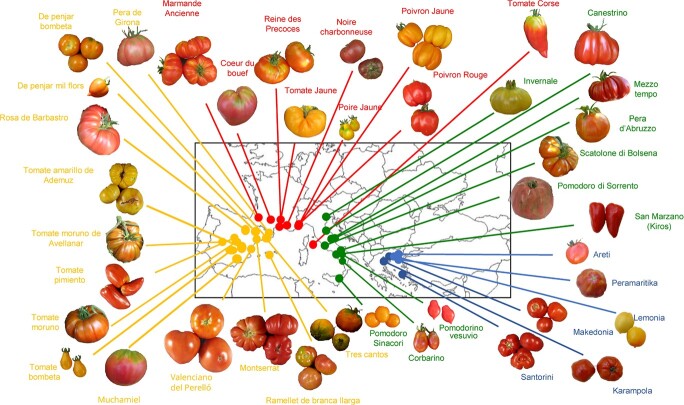
**Overview of European traditional tomato fruit variability still present in local markets.** Pictures of representative fruits of several traditional varieties. The traditional cultivation site of each variety is indicated on the map. The colour of lines and names indicate the country of origin: Spain (orange), France (red), Italy (green), and Greece (blue). See appendix file for higher resolution images

Although the “traditional” diversity has suffered a strong genetic erosion because of the replacement of traditional varieties by modern ones, a large number of traditional varieties can still be found in local markets (Fig.1) and are highly appreciated by local consumers. For instance, we find “Muchamiel”, “Moruno” [13], “Montserrat” and “Pera de Girona” [14] in Spain; “A Pera Abruzzese”, “San Marzano”, “Scatolone di Bolsena” or “Pomodoro di Sorrento” [15] in Italy; “Coeur de Boeuf” and “Marmande in France, and “Tomataki Santorinis” [16] in Greece. In addition, we find the Long Shelf Life (LSL) varieties “Penjar” and “Ramellet” in Spain [10], and “Da Serbo” or “del Piennolo”, “Corbarino” [17, 18], “Pomodorino Vesuvio” or “Sinacori”, in Italy [19]. This diversity is scarcely used outside of traditional cultivation areas or in recent modern breeding programs. Thus, traditional European diversity can be useful reservoir for genes that could be used to improve commercial varieties, not only to regain devalued consumer appreciation of tomato but also because traditional European tomato germplasm is well adapted to local environments. A greater knowledge about this tomato genetic resource is an important step for exploiting it. The former analyses of tomato variation have been mainly focused on differences between cultivated and wild species, in regard to domestication and modern plant breeding events [5, 20–26]. In contrast, information about the phenotypic and genetic variation present in traditional European tomatoes is still limited. Most studies have only been directed to a limited number of tomato varietal groups from Spain [10, 27], Italy [15, 28, 29], Greece [30] and Bulgaria [31]. Despite all of these valuable reports, there are no comprehensive studies that broadly cover the phenotypic variation of traditional tomatoes, and the underlying genetic diversity across Southern Europe.

In a recent work, 1044 European tomato accessions were classified into 27 landrace genetic groups and accessions, representing true vintage cultivars differentiated from “traditionalized” materials [12]. In the present report, we have extended this collection of traditional European tomatoes to 1499 accessions to thoroughly study the phenotypic diversity and its molecular underpinnings, and to provide a roadmap for using these untapped resources for tomato breeding.

## Results

### Overview of traditional European tomato phenotypic diversity

We established the TRADITOM collection (hereafter referred to as TRADITOM) by gathering 1499 traditional tomato genotypes from seed banks and research centre’s collections from Spain, Italy, France, and Greece (Supplementary Fig.1, Dataset S1 and Supplementary methods). With a few exceptions, the accessions represent varieties that had been cultivated in the Mediterranean basin for some time between 1950 and 2015 (Supplementary Fig. 1 and Dataset S1). These varieties represented the different end use classes in Southern Europe: fresh market (FM), processing (PR) or long shelf life (LSL). We phenotyped the collection and divided it into different sets (Supplementary Fig. 1), for seventy traits related to plant architecture and fruit shape, size, colour, and quality (Supplementary Table 1 and Supplementary methods), in ten different field trials across five countries, according to the current local cultivation practices (Dataset S2). Curated phenotypic data is available in Dataset S3.

**Figure 2:**
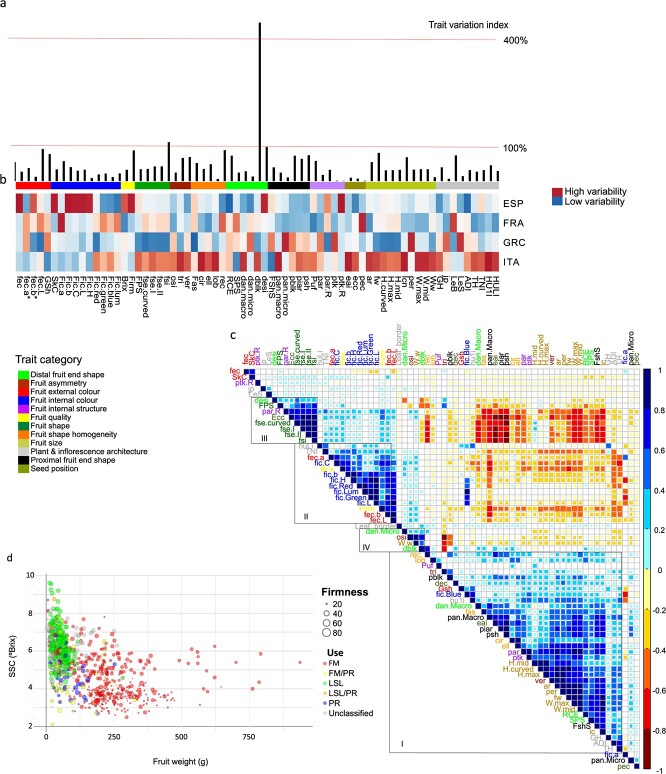
**Phenotypic variation and correlation between traits in European traditional tomato.** a) Bar diagram depicting global trait variation. Trait variation expressed as the index of qualitative variation (IQV) and the coefficient of variation (CV) for qualitative and quantitative variables, respectively. b) Heatmap comparing the amount of trait variation among countries. c) Spearman rank clustered correlation matrix of the phenotyped traits in the 9 trials. Large squares represent strong correlations and smaller squares represent weaker correlations. The colour scale indicates the correlation degree between traits, where 1 denotes a completely positive correlation (dark blue) and − 1 denotes a completely negative correlation (dark red) between two traits. Only significant correlations are shown (p < 0.01). The colour of the traits indicates the trait category. d) 4-D bubble plot displaying the relationship between fruit weight, SSC (°Brix) and fruit firmness and use. The x-axis represents fruit weight in g, the y-axis represents °brix, the bubble size represents firmness, and the bubble colour the traditional use. See appendix file for higher resolution images

Most of the analysed traits showed extensive phenotypic variation (Figure 2a and Dataset S4), with a variation index ranging from 1% to 447%. To study regional differences, we compared variation indexes, trait distribution, and averages between countries of origin (Figure 2b, Dataset S4 and Supplementary Fig. 2 to 10). Generally speaking, the Spanish accessions (ESP) were the most variable in fruit colour and quality traits, whereas the Italian (ITA) ones were variable in fruit morphology and plant architecture. The French (FRA) and Greek (GRC) accessions showed the highest variability for specific traits related to external and internal fruit colour, proximal and distal fruit shape, internal fruit structure, and plant architecture *(*Figure 2b). Also, we found significant differences (p < 0.001) in trait distribution and averages between countries of origin (Dataset S4 and Supplementary Fig. 2 to 10). Briefly, most traditional tomatoes had an indeterminate growth habit (Supplementary Fig. 2), but ITA were relatively rich in varieties with determinate (14.2%, *d_ij_* > 4) and semi-determinate growth habits (9%, *d_ij_* > 2). ESP had a significantly higher average plant height (*TH*) and average distances between inflorescences (*ADI*) than accessions from the other countries (more than 200 cm high and 29 cm average *ADI*), whereas FRA had the highest average plant height until the first and last inflorescence (*Hu1I, HuLI*), and total number of inflorescences (*Tni*) (Dataset S4 and Supplementary Fig. 2). The smallest plants were found in ITA (average *TH* = 147 cm; Dataset S4). Furthermore, ESP was mainly composed of two tomato types: red with yellow skin (51.5%), and pink with colourless skin and high firmness (45%, *d_ij_* > 4), the latter type mainly corresponded to LSL tomatoes (Supplementary Fig. 3 and 4). Most tomatoes (85%) were red, yellow tomatoes were more frequent in ITA (3.93%, *d_ij_* > 2), and yellow (6.42%, *d_ij_* > 2) and purple tomatoes (0.92%, *d_ij_* > 2) in FRA (Supplementary Fig.3). In addition, as compared to the other countries, the ITA fruits had the highest SSC and were the smallest, the reddest and the firmest, while the biggest, heaviest, and most loculated fruits were from FRA (Dataset S4 and Supplementary Fig. 3 to 5). Moreover, flat was the predominant fruit shape in TRADITOM, especially in GRC (~62%, *d_ij_* > 4) (Supplementary Fig. 6). The most elongated fruits were mainly found within ITA (Supplementary Fig. 6 and Dataset S4). Ellipsoid, heart, long, and obovoid fruit shapes were enriched in ITA (>10% each, *d_ij_* > 2); in contrast, these shapes were present, in most cases, in less than 5% of accessions from other countries or even absent, such as the heart shape in FRA. Furthermore, ITA and GRC fruits showed a higher obovoid asymmetry (high *osi*), and ESP and FRA fruits had a higher triangle asymmetry (Supplementary Fig. 7 and Dataset S4), with ESP being rich in oxheart shaped fruits (10%, *d_ij_* > 2) (Supplementary Fig. 6). Moreover, non-fasciated fruits predominated in general in TRADITOM (Supplementary Fig. 8), particularly in GRC, where they accounted for 98.5% of the accessions analysed. In contrast, ESP varieties were relatively richer in fruits showing severe fasciation, while ITA was rich in low fasciated ones (Supplementary Fig. 8). Proximal and distal fruit end were also different between countries of origin. FRA and ESP presented more pronounced and more intense green shoulders than GRC and ITA (Supplementary Fig. 3 and 9). Furthermore, ESP was rich in fruits with a weakly-ribbed calyx end (*rce* = 60%, *d_ij_* > 4) and stellate shape of the pistil scar (*sps*, 26%, *d_ij_* < 4), GRC in fruits with strong and intermediate *rce* (35%, *d_ij_* < 2) and irregular *sps* (42%, *d_ij_* > 4), ITA in fruits with weak *rce* (32%, *d_ij_* > 4) and dot *sps* (76%, *d_ij_* > 4) and FRA fruits in stellate *sps* (29%, *d_ij_* < 2) (Supplementary Fig. 10). In summary, there were differences in trait diversity among the countries of origin, perhaps indicating selection for local adaptations or for regional gastronomic preferences.

Correlations between all pairs of traits (Figure 2c and Supplementary results) revealed groups of five or more traits showing moderate or strong correlations between them (R^2^ = 0.25–1, p-value<0.01). As expected, most traits within a trait category were in the same correlation group, and most likely reflected strong developmental associations between these traits. Furthermore, correlations between traits belonging to different phenotypic categories were also found (Figure 2c). For example, many traits associated to fruit shape and size correlated to external and internal fruit colour and plant architecture (group I) traits, fruit quality with colour parameters (group II), and obovoid shape to strongly serrated leaf borders (group IV). In addition, moderate to strong negative correlations were found for most traits in group I with respect to the other groups. In short, the correlation analysis indicated that the biggest European traditional tomato fruits tended to be mostly flat, round, heart and oxheart, soft, pale, with a poor SSC, with uneven transversal shape, and were mostly produced by indeterminate plants with a low number of inflorescences, whereas long, obovoid and small fruits tended to have more intense colours, higher SSC and firmer fruits, and were produced by indeterminate plants with a large number of inflorescences.

The four-dimensional bubble plot analysis of important breeding traits, such as size, SSC, firmness, colour or shape, together with traditional end use (Figure 2d and Supplementary Fig. 11) indicated the presence of combinations of traits that did not follow the main trends observed in the correlation analysis (Figure 2c). For example, in spite of the negative correlation between fruit size and SSC, fruit size and firmness, or fruit size and colour, some traditional varieties produced large (>300-400 g) and firm (>60 units) tomatoes with a high SSC and fec.a^*^ values (indicative of a high red colour), and therefore high lycopene content (Figure 2d and Supplementary Fig. 11). On the contrary, some accessions with small fruits and poor SSC were also found (Figure 2d and Supplementary Fig. 11). Furthermore, despite the general positive correlation between long or obovoid fruits (with high *fse.I*) with SSC (Figure 2 and Supplementary Fig. 11), TRADITOM also contained varieties with round and flat (*fse.I* ≤ 1) fruits with good SSC (Supplementary Fig. 11).

### Modelling traditional European tomatoes

**Figure 3:**
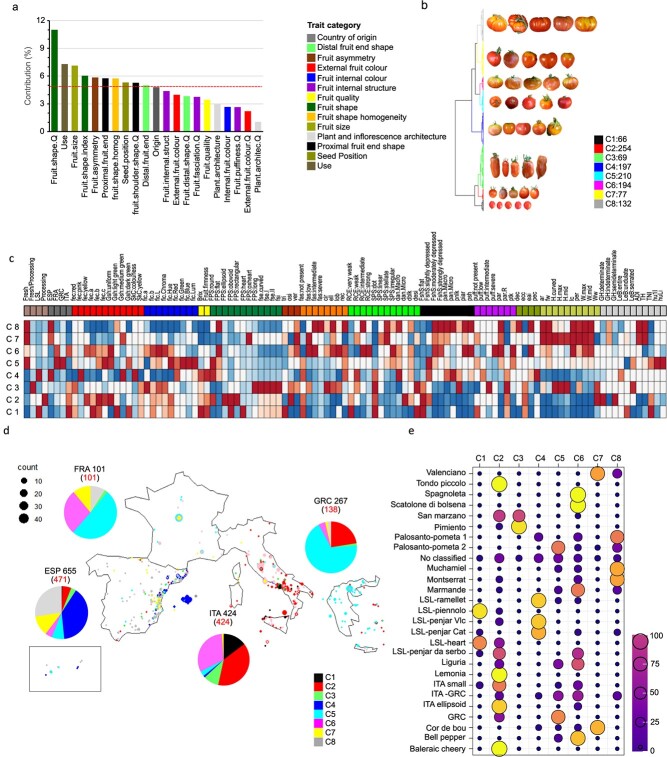
**Phenotypic map of traditional European tomato**. **a)** Bar-plot of the contribution of each group of variables to the total variance explained by the first three dimensions (C_1,2,3_). Only groups of variables with C_1,2,3_ > 1% are shown. The red dashed line on the graph indicates the threshold to consider a group of traits to have an important contribution to the global variability. Trait category followed by Q indicates qualitative groups of traits as detailed in Supplementary table 1. **b)** Dendrogram showing the eight phenoclusters resulting from the HPCP on the first five dimensions of the MFA. Tomato images correspond to five representative tomato accessions cluster. Each cluster is represented by a colour. The number of accessions in each cluster is indicated. **c)** Heat map depicting the phenotypic, usage and country of origin differentiation among phenoclusters. The colour code depicts the enrichment of each variable in each cluster, ranging from over-represented (dark red) to under-represented (dark blue) **d)** Geographical distribution of the phenoclusters. The pie charts indicate the proportion of accessions assigned to each phenocluster per country according to colour code depicted in 4b. The number of accessions per country in the collection and the georeferenced accessions used in MFA-HCPC are indicated in the pie-chart in black and red numbers, respectively. The dots in the map are proportional to the number of accessions georeferenced in each location. **e)** Bubble plot showing the representation of the traditional European genetic groups identified in ref. [12] in each phenocluster. The size and the colour of the bubble represent the percentage of each genetic group per phenocluster. See appendix file for higher resolution images

The analyses described above indicated a high geographic phenotypic variability, which may reflect local adaptations, regional gastronomic preferences and/or genetic founder effect. However, we found consistent trends of trait co-occurrence patterns in TRADITOM (Figure 2c). To model traditional European tomatoes, we used a multifactorial analysis (MFA [32, 33]), which allows us to analyse data of different nature together, followed by a hierarchical clustering of the MFA principal components (HCPC [34]). We analysed phenotypic data with country of origin and end use simultaneously (Dataset S5). Contributions and relationships of the groups and individual variables are depicted in Figure 3a and Supplementary Fig.12, respectively. The groups of traits that most contributed to the first three MFA dimensions (Figure 3a), and which therefore globally differentiated traditional European tomatoes, were *Fruit shape visually determined* (C_1,2,3_ = 11%), *Fruit size* (C_1,2,3_ = 7.13%), *Fruit shape index* (C_1,2,3_ = 6.04%), *Fruit asymmetry* (C_1,2,3_ = 5.84%), *Proximal fruit end* (C_1,2,3_ = 5.76%), *fruit shape homogeneity* (C_1,2,3_ = 5.74%), *Seed position* (C_1,2,3_ = 5.29%), *Fruit shoulder shape visually determined* (C_1,2,3_ = 5.26%) and *Distal fruit end* (C_1,2,3_ = 4.99%). Furthermore, the MFA indicated that *use* had an important contribution to TRADITOM variability (C_1,2,3_ = 7.31%) (Figure 3a and Supplementary Figs 12 and 13). Collectively, fruit morphology traits and end use, rather than country (Figure 3a, Supplementary Figs.12 and 13), were key factors driving traditional tomato diversification in Europe. Fruit colour, which did not show an important contribution to the overall variability (Figure 3a), but correlated with country in the second dimension (Supplementary Fig.12), seemed more likely to be related to appearance preferences in each country.

Next, using HCPC, we objectively classified accessions into eight homogeneous agromorphological clusters or phenoclusters (see methods for cluster number determination) (Figure 3b, c and d). Supplementary Table 2 shows details about the phenotypic, usage, and country profiles of each cluster. The geographic-space plot of accessions (Figure 3d) shows phenotypic divergence of phenoclusters between countries. ITA accessions were predominant in phenoclusters C1, C2, C3, and C6, ESP accessions in C4, C7, and C8, and GRC and FRA ones in C5, C6, C7, and C8, while most GRC accessions belonged to C2. Furthermore, some varieties belonging to FM and PR phenoclusters (C2, C3, C5, C6, C7, and C8, Figure 3c) were collected in different countries (Figure 3c, d), revealing a steady flow of these varieties through Southern Europe. In contrast, LSL accessions, represented by phenoclusters C1 and C4, were restricted to specific areas in Italy (C1) and Spain (C4) (Figure 3c). To further characterize the observed phenotypic structure, we explored the genetic composition of phenoclusters based on the genetic classifications performed by ref. [12] in a subset of this tomato collection (Figure 3e and Supplementary Table 3). The comparison of phenotypic and genetic class rank1 classification, differentiating true vintage from modern and wild tomato [12] (Supplementary Table 3), indicated that approximately 30% of the accessions in phenoclusters C2 and C5 were not true vintage tomatoes (“traditionalized”). Furthermore, phenoclusters and ref. [12] genetic class rank2 classification (which included only the true vintage accessions), showed a good agreement. For 24 out of 26 genetic groups, more than 50% of the accessions were in the same phenocluster. Out of these, 18 genetic groups were represented in a proportion greater than 70% in a single phenocluster (Figure 3c).

### GWAS meta-analysis of 67 traits identified 211 loci

To study the genetic basis of TRADITOM diversity, and to identify variants involved in that diversity across populations, we analysed phenotypic (Dataset S3) and genotypic data (Dataset S6) using a meta-GWAS approach. The GBS results, linkage disequilibrium, and overall levels of genetic diversity, are available as Supplementary results, Dataset S6–7, Supplementary tables 4–5 and Supplementary figures 14–15. First, we performed a GWAS with a mixed linear model for eight individual phenotyping trials with more than 100 accessions, and 1303 to 2086 informative (MAF ≥ 0.05) SNPs (depending on the composition of the trait) (Supplementary Fig. 1). Since the traditional European tomato had a strong population structure [12] and relatively high LD (average LD decay at cut off *r*^2^ = 0.1349 was 1.74x10^6^ bp) (Supplementary Fig. 15 and Supplementary Table 4), population structure and family relationships were controlled in each panel by using a PCA and genomic kinship matrix. In total, we identified 776 significant associations in 144 loci using individual GWAS panels (p < 3.8x10^−5^, Supplementary Table 6 and Dataset S8).

**Figure 4:**
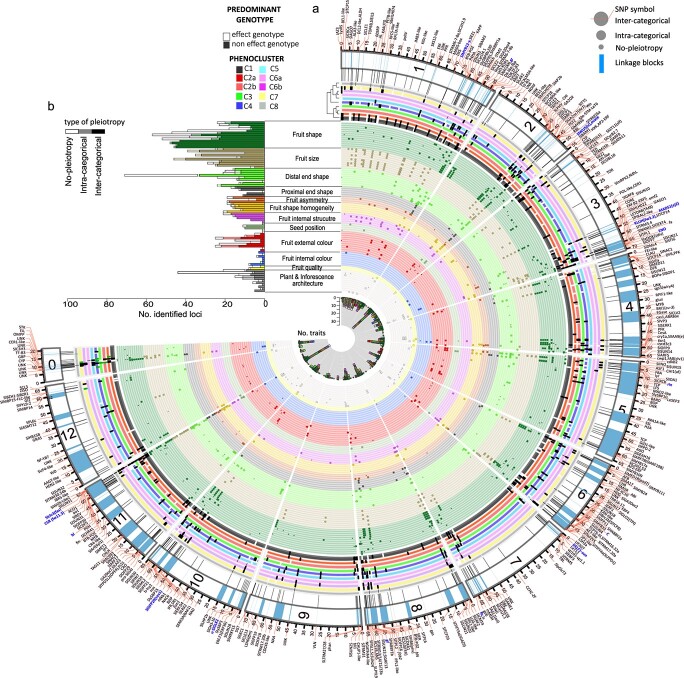
**Roadmap of European traditional tomato**. Fuji-plot representing the 1486 SNP-trait associations identified by meta-analysis in 581 SNPs located in 211 loci, their colocalization, the predominant genotype in each phenocluster and candidate genes. a) Loci associated to each trait (p-value<10–5), the colocalization and genotype. The inner-most ring (ring 1) represents the number of traits associated for each SNP. Rings 2–14 represent the genomic position of the SNP associated to trait, trait category and the type of colocalization: Larger dots inter-categorical colocalization, middle dots, intra-categorical and small dots, trait specific association. Rings 15–24 represent the phenocluster and the most frequent allele present in the accessions of each cluster. The dendrogram shows the hierarchical relationship of clusters. Only alleles present in more than 60% of accessions in each phenocluster are represented. Effect genotype in white and non-effect genotype in white. The outer ring represents chromosomes and linkage blocks around trait-associated SNPs. b) The number of identified trait-associated SNPs for each trait, grouped by type of colocalization. See appendix file for higher resolution images. The order of the traits in each trait category are (from the outer-most ring): **Fruit shape:***fps*_flat, *fps*_round, *fps*_ellipsoid, *fps*_ovoboid, *fps*_rectangular, *fps*_heart, *fps*_oxheart, *fps*_long, *fps*_bell_pepper, fse.curved, fse.I, fse.II, fsi; **Fruit size**: ar, H.curved, fw, H.mid, lcn, H.max, W.max, per, W.mid, W.w; **Distal fruit end shape:** rce, sps_dot, sps_irregular, sps_linear, sps_stellate, dan.macro, dan.micro, desi, dblk; **Proximal fruit end shape**: FShS, pan.macro, pblk, piar, psh; **Fruit asymmetry:** tri, osi, ver; Fruit shape homogeneity: fas, cir, ell, lob, rec; **Fruit internal structure**: puf, par, par.R, ptk, ptk.R; **Seed position:** dec, ecc, eai, pec; **Fruit external colour**: fec_yellow, fec_orange, fec_red, fec_pink, fec_purple, fec_brown, GSh, SkC, fec.a^*^, fec.b^*^; **Fruit internal colour**: fic.a, fic.C, fic.H, fic.L, fic.green, fic.lum, fic.red; **Fruit quality**: firm, SCC; **Plant & inflorescence_architecture**: GH, jp, LeB, LeS_potato, LeS_regular, LeS_double_feathered, ADI, Hu1i, HuLi, TH, Tni

Then, all trait associations in each of the eight GWAS panels were re-analysed using GWAS meta-analysis, penalized for within–data set residual genomic inflation to control for residual population structure, cryptic relatedness, polygenic inheritance, and genotyping errors [35, 36]. A total of 1315 accessions (mostly phenotyped for a given trait at least twice) and 3426 SNPs were used for this analysis. The meta-analysis identified 1486 significant associations (p < 10^−5^), detected in at least two individual GWAS panels, mapping to 581 SNPs located in 211 loci (Figure 4a and Dataset S9). Seventy-four loci included a single lead SNP, while 137 loci were composed by several SNPs in LD blocks. Only 16% of the identified SNPs were found at low frequencies (MAF <0.1) (Dataset S9). The direction of associations (positive or negative) were consistent across trials in more than 70% of the cases. Among the associations identified in the meta-analysis, 346 had moderate to high heterogeneity (251 associations had moderate heterogeneity (I^2^ > 50%), and 95 had >75% high heterogeneity) due to differences in the direction or magnitude of the allelic effect (Dataset S9). Differences in ancestry, LD, allele frequency, and genotype x environment interaction among different European subpopulations, may have contributed to this heterogeneity [37].

The comparison of the associations obtained in the meta-analysis with those obtained in individual GWAS panels indicated that 119 loci (306 associations) were common between both types of analyses, 92 loci (1180 associations) were specific to meta-analyses, and 25 loci (295 associations) were specific to individual GWAS panels (Dataset S10). Among the latter, 22.4% of them corresponded to fruit colour associations. Furthermore, 30% (174) of the SNPs identified by meta-analysis included variants found at low frequencies (MAF < 0.1) in individual GWAS panels (see min_freq in Dataset S9), highlighting that the increased population size obtained in the meta-analysis allowed us to identify associations to variants found at very low frequency or even absent, in the individual sub-populations.

A further inspection showed that the meta-analysis associations were not evenly distributed across the genome, with 76.24% (1133) of the associations concentrated in 39.24% (228) of the SNPs (Figure 4a, Supplementary Fig. 16 and Dataset S9). These regions would include genes with pleiotropic effects or multiple linked genes controlling different traits. Furthermore, we found several pleiotropic hotspots of SNP-trait associations, such as SL2.50ch11p55069352, where up to 35 traits, included in fruit morphology, size and colour categories, were associated with that SNP (Figure 4b and Dataset S9). In fact, this genomic region includes *fas*/SlCLV3 [38, 39], which has important pleiotropic effects in plants and reproductive organ architecture, and in altering the expression of genes involved in additional traits [40]. Multiple trait co-associations in the genomic regions on chromosomes 2 and 11 have been previously reported in several works (See references in Dataset S9).

The occurrence of association with multiple traits was classified into three classes: inter-categorical (traits of different trait categories associated with the same SNP), intra-categorical (traits of the same trait category associated with the same SNP) or non-pleiotropic (Figure 4c and Dataset S9). In total, 23.75% (353) of these associations were non-pleiotropic, 10.29% (in 57 SNPs) presented intra-categorical pleiotropy and 65.9% (in 171 SNPs) inter-categorical pleiotropy. We found inter-categorical pleiotropy mostly between different fruit morphology categories and/or fruit size, but also between fruit colour and/or fruit quality traits with fruit morphology, size and/or plant architecture (Figure 4). Therefore, many of the identified pleiotropies comprised phenotypic correlated traits (Fig 2c), biologically related traits, or the same trait phenotyped in different ways. At the loci level, the extent of the pleiotropies was extremely high (Dataset S9), with 89.37% inter-categorical, 3.36% intra-categorical pleiotropies and 7.27% non-pleiotropic, which may be partly explained by different linkage of casual SNPs in LD blocks.

### Identification of novel loci

Overall, for 903 associations in 102 loci, there was previous evidence of association for the same trait or related, within or close to the defined candidate gene region (see Dataset S9). Among the polymorphic loci previously reported for the same/related trait, the previously-identified causal genes were: colourless fruit epidermis (*y*/SlMYB12) [41], green ripe (*gr*) [42], locule number (*lc*/*SlWUS*) [9], *ovate* [43], *fw3.2* (*SlKLUH*) [44], excessive number of floral organs (*ENO*) [45], potato leaf (*C*) [46], ripening inhibitor (*rin)* [47], insertion place of sun (DELF1/*sun*) [48, 49], flacca (*flc*) [50, 51], green flesh (*gf*) [42], uniform ripening (*u*/SlGLK2) [52], suppressor of ovate (*sov1*/SlOFP20) [53], blind (*bl*) [54], fasciated (*fas*/SlCLV3) [39], and cell size regulator (*fw11.3*/CSR) [55] (Figure 4 and Dataset S9).

Notably, 583 trait associations located in 159 loci were novel. Candidate genes (defined in previous works by analysis of chemical, physical or biotechnological-induced mutations) were found in a large proportion in these loci. (Supplementary Table 15). As an example, genes involved in fruit size (SlEZ1 [56], *fab*2 [39], SlMYB33 [57] and SlIAA17 [58]), fruit morphology (SlTRM5 [53], SlGT11/*slf* [59], cdc2a1/ SICDKA1 [60], *div*/*fb*/Slα-DOX2 [61, 62], TAGL1/*arl* [63]^,^ and TM8 [64]), plant and inflorescence architecture (SlBAM1 [65], *div*/*fb*/Slα-DOX2 [61, 62], *fin* [39] and *fa* [66]), fruit colour (ZISO [67], SlbHLH022 [68], CHI1/*af* [69], TAGL1/*arl* [63], SlNAC1 [70], and CRY1a [71]), and fruit quality (mMDH [72] and SlNAC1 [70]) (Figure 4).

### Integration of phenotypic and GWAS meta-analysis

Finally, to identify key factors that may explain the origin and development of the phenoclusters observed within traditional European tomatoes (Figure 3), we repeated the MFA-HCPC analysis but simultaneously combining phenotypic data, region of origin, traditional use, and the genotypic matrix, for 581 trait associated SNPs from the meta-analysis. The genotypic, phenotypic, geographic, and usage profiles and accessions in each cluster are found in Dataset S11. The MFA-HCPC analysis accurately reconstructed the previously found phenoclusters (Fig. 3 and Figure 4a, Supplementary Fig. 17). Furthermore, accessions in phenoclusters C2 and C6 were subdivided by country of origin, with most of ITA retained in subclusters C2a and C6a (with 99% and 80%, respectively) and separated from accessions coming from other countries in subclusters C2b and C6b (Dataset S11 and Supplementary Fig. 17).

To obtain a genetic signature for the accessions belonging to each phenocluster, we selected those SNPs with a genotypic frequency > 60% within a phenocluster (Figure 4a and Dataset S12). Overall, we found genetic divergence among phenoclusters. A detailed inspection indicated that none of the SNP genotypes was specific to a single phenocluster (Dataset S11), but rather we identified overlapping and shared signatures across phenoclusters (Figure 4a). Furthermore, we also found evidence of phenotypic convergence between C2 and C6 ITA tomatoes and the rest of the accessions from other countries, where a different set of signatures in different loci caused similar phenotypes (Figure 4a).

## Discussion

Southern Europe is a secondary centre of tomato diversification. However, whilst phenotypic and genotypic variabilities associated to the primary crop domestication and diversification that occurred in Meso and South America have been extensively studied [5, 20–26], there is no comprehensive phenotypic and genotypic study covering the large extent of cultivated traditional Southern European tomatoes. Here, to unveil the molecular basis of Southern European tomato phenotypic diversity, we carried out the most comprehensive phenotypic and genomic variability analysis on traditional European tomato by using a collection of 1499 traditional European tomatoes. Our work is unique in several aspects.

First, the extent of phenotyping and modelling performed in the largest tomato collection thus far allowed us to identify the main agromorphological Southern European tomato types. The geographical and usage distribution of the varieties defining the phenoclusters reflect a constant steady flow over the years for the FM and PR varieties across countries, although not for LSL tomatoes. Second, with the GWAS meta-analysis, we identified and cross-validated 1486 associations for 70 traits in 211 loci, several of which with potential pleiotropic effects. Some of them would not have been identified by analysing individual GWAS panels due to the small population size or low polymorphism frequency in individual populations. Most importantly, 583 trait associations in 159 loci were novel findings. And third, we identified molecular signatures and loci combinations that revealed different genetic histories and the underpinnings of the phenotypic variation of different tomato types in Southern Europe. However, it remains unknown whether these loci act independently or if epistatic interactions between them influence the phenotypic outcome.

The results presented here revealed a broad range of phenotypic variability in traditional European tomato, especially for those traits related to fruit shape and size, mainly associated to the different culinary end use in each country and region. Moreover, we found a higher phenotypic diversity in the ESP and ITA accessions, which is consistent with those countries being the main diversification centres of tomato in Europe. However, while the ESP accessions were the most diverse in fruit colour and quality traits, ITA accessions were in shape, size and plant architecture traits, in congruence with previous studies involving ITA landraces [28]. In contrast to domestication and early improvement steps that occurred in America, where selection was mostly unidirectional (towards a lower number of inflorescences carrying larger fruits, with thicker pericarps, more locules, and lower citric acid, lower soluble solids, and lower beta-carotene contents [22, 24, 26]), the diversification in Europe was divergent, generating new phenotypes. Thus, among the traditional European varieties, we found a range of small fruited varieties (< 5 g) to large fruited varieties (> 900 g), with all of them cultivated in different regions. Most importantly, some accessions were selected with combinations of desirable traits, such *as* high fruit weight and high sugar content, that are usually not found in other collections, as they are negatively correlated in *Solanum lycopersicum [**25**]*. Similar unusual trends were found for most of the traits, described as domestication syndrome traits [22, 24, 26]. Furthermore, the phenotypic diversification of European tomato has been driven towards three tomato types (FM, PR, and the particular European traditional type, LSL), rather than two as previously reported [20].

Finally, our work also indicated that despite the low polymorphism reported for cultivated [73] and traditional tomato germplasm (here and ref [12]), as compared to the wild relatives, traditional European tomato is a rich repository of crop genetic diversity, as revealed by new, previously unreported loci. New specific uses and taste preferences of European inhabitants, gene flow, and patterns of natural and farmer-mediated selection, historical events, as well as ecological growing conditions and traditional management, could have led to the selection of new mutations, as described for *SUN* [9] or *alc* [10], or to the increase and maintenance of the frequency of alleles that could have been at low-frequency in the European tomato ancestors. The fact that 102 of the loci identified in this work were already involved in American domestication and early diversification, suggested that these loci were included in the initial diversity that arrived in Europe; although we cannot exclude a sporadic gene flow between American and Southern European tomatoes during the 500 years of cultivation of this crop in Europe or the “traditionalization” of obsolete commercial cultivars [12]. Besides, some previous identified genes such as *fw2.2*/*CNR* [74] and *GLOBE* [75] did not show an association with fruit traits in the current GWAS, similarly to Sacco et al. [76]. *fw2.2/CNR,* involved in the early domestication, is likely fixed in the traditional European varieties. It could be not variable already in the original imported American germplasm, or farmers fixed it quickly by intuitive selection. *GLOBE* [75], a recent mutation affecting fruit shape identified in modern North American varieties, would not be variable in the traditional European gene pool.

Thus, the tremendous phenotypic variation observed in the present work is the result of new combinations of alleles selected from the initial, or in some cases new, genetic variation, which generated the present-day European tomato diversity. Structural variants [77], transposable elements [78], new epistatic interactions, and the uncovered cryptic variation [79] may have also played a role in generating traditional European tomato diversity. This paper provides a roadmap for breeding superior genotypes using traditional varieties with a combination of traits that are often difficult to find in modern varieties.

## Materials and methods

### TRADITOM collection

The TRADITOM collection comprises 1499 accessions which includes the 1044 European accessions analysed by ref. [12], plus 455 additional traditional European accessions. The collection is composed of 658 accessions collected in Spain, 425 in Italy, 267 in Greece, and 116 in France (three of them were original from the Galapagos Islands). In addition, 4 accessions from the Ukraine, 4 from Israel, and 15 from EU collections but with unknown collecting site were also included. Passport information and culinary use is provided in Supplementary Fig.1, Supplemental table 1 and Supplemental methods. The map in Supplementary Fig. 1 was generated using ArcGIS® software (Esri).

### Phenotyping of the TRADITOM collection

Subsets of the TRADITOM collection were cultivated and evaluated in 10 locations in 5 countries (Supplementary Fig.1) in the experimental fields of COMAV-UPV, FMA-UPC (Spain), INRA (France), UNITUS, ARCA2010-CNR (Italy), and ACTYMPAKY-Aristotle University of Thessaloniki (Greece) during spring–summer 2015, and in HUJI-ARO (Israel) during autumn-winter 2015–2016, where the entire collection was grown, with each of them following their common cultivation practices (for details of each trial and cultivation practices see Dataset S2). There was one plot per accession with five to seven plants per plot (Supplementary Fig. 1). Phenotypic traits belonged to twelve trait categories (Supplementary Table 1): fruit external colour, fruit internal colour, fruit quality, fruit size, fruit shape, distal fruit end shape, proximal fruit end shape, fruit asymmetry, fruit shape homogeneity, fruit internal structure, seed position and plant and inflorescence architecture. These traits were analysed in all trials, except for HUJI-ARO, where only the qualitative traits, average distance between inflorescences, fruit weight, soluble solids content (SSC), and locule number were scored. Detailed information about standardized phenotyping procedure and scoring is provided in Supplementary methods.

### Trait pre-processing

Pre-processing of phenotypic data was performed to detect outliers and assess trait reproducibility. The Rstudio package [80] was used. Qualitative descriptors for each accession recorded in the different trials, which should have a constant phenotypic expression in all environments, were set to one value. If the phenotypic score for a descriptive trait was distinct among trials, images and raw data were checked and the data corrected. In the few cases where phenotypic call errors were impossible to correct, data were defined as missing. Accessions showing a clear variability for one qualitative descriptor were removed. In the case of quantitative traits, the reproducibility was evaluated by checking distributions and sampling fruits from each trial. Outliers were handled using inter-percentile range (IPR = P_0.99_-P_0.01_). Values falling outside the P_0.01_- (1.5^*^IPR) and P_0.99_+ (1.5^*^IPR) range were removed. Clean data was averaged. In the case of morphological quantitative data, where measured variables were interdependent, missing data was imputed by the regularized iterative Principal Component Analysis (PCA) algorithm using the missMDA R package [81]. Imputation was performed using a matrix composed of accessions with less than 30% of missing morphological data. The number of components for the PCA imputation were estimated by cross-validation. Three components leading to the smallest mean square error of prediction (MSEP) were chosen.

The deviation from normal distribution was checked by visual inspection of histograms and Q-Q plots. Fifty quantitative traits followed a roughly normal distribution. Fic.b^*^ and *Fic.C* had a bimodal distribution, and *fse.curved, dec, tri, pec and ptk.R* presented high skewness and kurtosis (Dataset S4). Traits exhibiting a significant deviation from normality were transformed using logarithmic, square-root, arcsine of square root, hyperbolic arcsine of square root transformations, respectively, and tested for normality. None of these transformations fitted the normal distribution, so we used the raw data for further testing.

A table with all qualitative and averaged quantitative traits per accession and trial was consolidated after trait pre-processing for further analyses (Dataset S3). Because of missing data for some traits, the total number of accessions varied by trait.

### Descriptive statistics

Mean, standard deviation, variance, and maximum and minimum values were calculated for each quantitative trait using summarytools 0.9.4 [82]. In the case of discrete random variables (ordinal and nominal) the mean (μ_x_) or expectancy [E (X)] was calculated as E (X) = μ_x_ = ∑ [x_i_·p (x_i_)], and the variance as [inline graphics]_x_^2^ = ∑ [x_i·_- μ_x_]^2^·p (x_i_)]; where x_i_ is the scoring number and p (x_i_) the proportion of accessions scored as x_i_. In the case of binary variables (recorded as 0 or 1), the mean (μ_x_) or expectancy [E (X)] was calculated as E (X) = μ_x_ = p (x_1_), and the variance as [inline graphics]_x_^2^ = p (x_1_) [1- p (x_1_)], where p (x_1_) is the proportion of accessions scored as 1. Quantitative trait variability was evaluated with the coefficient of variation (CV), and qualitative variation index (IQV) as the ratio of the total number of differences in the distribution to the maximum number of possible differences with the same distribution [83]. A heatmap comparing trait variation among countries of origin was obtained with clustvis [84] with centred and unit variance scaled trait variation indexes.

### Assessment of statistical differences between countries

Trait variation between countries for normal quantitative traits was assessed with an one-way ANOVA, and pairwise mean comparisons between countries were performed with Tukey’s honestly significant difference (HSD) test (p < 0.05) using the Rstudio package [80]. Non-normal quantitative traits were analysed using the Kruskal-Wallis rank sum test for differences between countries, followed by Mann–Whitney pairwise comparisons, and corrected for multiple comparisons with “dbplyr” from the tidyverse R package [85]. The distribution of quantitative variables per country were visualized as violin plots using the gg2plot R package [86]. For the qualitative variables, the distribution was evaluated with the Chi-square (χ^2^) test with Bonferroni false discovery rate (FDR) for pairwise nominal and ordinal comparisons of the proportions using Rstudio [80]. Standardized Pearson’s residuals (*d_ij_*) were calculated to analyse the departure of each category from the expected values. Residuals with |*d_ij_*| > 4 have an approximate P-value < 0.001, and |*d_ij_*| > 2 have an approximate P-value < 0.05 [87]. The results were presented using mosaic plots with the “vcd” R package [88]. For testing country of origin differences, countries with less than 15 accessions were removed. Differences between country means or proportions for each trait were considered statistically significant at p-value <0.001. Bubble plots were generated using the ggplot2 package [86].

### Correlation analysis

The overall correlation between all traits was calculated using the corrplot package [89] with Spearman’s rho correlation coefficient in a matrix containing accessions with less than 30% missing data. Correlations were considered significant at p-value<0.01. Traits were clustered using Average/UPGMA distance as agglomerative.

### Multifactorial analysis and hierarchical clustering on principal components

A multifactorial analysis (MFA) was performed to assess common factors that explained European tomato variability with all quantitative and qualitative groups of traits with less than 10% missing data together with passport data (Dataset S5), in regard to traditional use and country of origin, using the FactoMiner and Factoextra R packages [90, 91]. The dataset contained 21 groups of variables that were organized into nine groups of qualitative variables (*Fruit external colour, Distal fruit end shape, Fruit internal structure, Fruit shape, Fruit shape homogeneity, Proximal fruit end shape, Plant & inflorescence architecture, Use and country of origin*) and twelve groups of continuous quantitative variables (*Fruit external colour*, *Fruit internal colour, Fruit quality, Distal fruit end shape, Fruit asymmetry, Fruit internal structure, Fruit Shape, Fruit shape homogeneity, Fruit size, Proximal fruit end shape, Seed position, and Plant & inflorescence architecture).* Traits in each group are indicated in Dataset S2. Continuous variables were scaled and standard software settings were selected. All variables were set as active. Variables were plotted in the plane described by the MFA principal dimensions. The squared correlations between variables (or group of variables) and the dimensions were used as coordinates. To define the groups of variables or variables that were the most important for explaining the variability in the dataset, we calculated the total contribution of a given group of variables or a variable in explaining the variation retained by the *n*-dimensions (C*_j,n_*) [92]. The contribution was calculated as C_j,n_= $\frac{\sum_{n=i}^N({C}_{j,i}\ast {\lambda}_i)}{\sum_{n=i}^N{\lambda}_i}$, where C*_j,i_* is the contribution of the variable *j* to the dimension *i*, and λ*_i_* is the eigenvalue of the dimension *i*. The contribution cut-off for a variable *j* was calculated assuming a uniform variable contribution, so that the value of expected contribution for the variable *j* to the dimension *i* is 1/*Y*, where *Y* is the total number of variables.

The hierarchal clustering on principal components (HCPC) was performed using the Factominer R package [34] on the scores of the first 5 dimensions of the MFA, using Euclidean as distance metrics and Ward’s criterion as the agglomerative algorithm. The optimal number of clusters was selected by satisfying three criteria (i) the large number of clusters that (ii) maximize the relative loss of inertia [34] and (iii) maximize the number of accessions per varietal type (varietal type was not included in MFA and HCPC calculations in order to perform an objective classification). The most important traits considered towards the decision of the cluster formation were presented according to their v.test value, calculated by comparing the proportion of accessions in the cluster sharing a trait, in comparison to the overall proportion. Traits with a p-value ≤0.0001 were considered significant. The sign of the v.test indicates whether the trait or average in the cluster is enriched in that variable (in the case of qualitative variables) or larger than the average of that variable in the complete dataset (in the case of quantitative variables). A heatmap depicting trait, country and use enrichment of each phenocluster was created with clustvis [84] with percentage or mean values centred and vector scaled by variable.

Balloon plots comparing phenotypic and genetic clusters described in ref. [12] were constructed in gg2plot [86]. Phenotypic clusters were mapped on the basis of the collecting site coordinates of 1134 georeferenced accessions using gg2plot and Global Administrative Areas of Natural Earth Vector and Raster Map Data [93]. In the case of non-georeferenced accessions, country coordinates were assigned.

### Genotyping by sequencing and variant calling pipeline

The genotyping by sequencing (GBS) data used in this analysis is based on 1118 European accessions which were already genotyped [12], plus data from 302 additional accessions. Genotyping was performed as described in ref. [12]. All GBS data are available in NCBI (https://www.ncbi.nlm.nih.gov/sra) under accession numbers PRJNA722111 and PRJNA774172. Fastq files were evaluated for sequencing quality. Sequences were mapped to the tomato reference genome version 2.50 together with the same subset of SNPs selected from the European accessions genotyped by ref. [12], re-sequenced accessions from the 150 tomato genome consortium [20], 8 parents from a tomato MAGIC population [23], and 350 accessions from a third re-sequencing initiative [26] as described in ref. [12]. The SNP called matrix was filtered to contain only accessions from the traditional tomato, with a minimum read depth 3, less than 30% missing data per SNP, maximum heterozygosity per SNP 10%, and less than 30% missing data per accession (Dataset S6). Missing SNPs were imputed using the Linkage Disequilibrium K-number neighbour imputation (LDKNNi) [94] algorithm implemented in Tassel 5.0 [95] with default LD search for KNNi 10 Mb, High LD Sites (*l*) of 30 and Number of nearest neighbours (*k*) of 20 (Dataset S7). Optimal *k* and *l* values were determined by testing combinations that minimize the error imputation accuracy in a matrix of random missing cells of 0.15%. The final genotype imputation error rate was 0.0045. Imputed data was used for downstream analyses.

### Genotypic diversity and linkage disequilibrium

The genetic diversity analysis was performed with Tassel 5.0 [95]. Nucleotide diversity (π), segregating sites, Watterson’s estimator (θ), and Tajima’s D were calculated for each SNP, using a non-overlapping sliding window of 100 bp, and then averaged over the total number of sites to obtain an average nucleotide diversity per bp. LD was calculated in TASSEL v5 for each chromosome, by computing *r*^2^ values for intrachromosomal pairwise marker comparisons using a sliding window size of 50 markers. The baseline *r*^2^ value, as evidence of linkage, was calculated at the 95^th^ percentile of the null distribution of inter-chromosomal root transformed r^2^_,_ using it only on markers showing a MAF >0.01 according to [96]. Intrachromosomal LD decay was estimated as the intersection of the LOESS curve fit to baseline *r*^2^ value using ggplot2 [86]. Block length per chromosome was defined as the average and maximum marker distance with LD above baseline r^2^ value.

### Genome-wide association analysis (GWAS) meta-analysis

GWAS was performed using the R package Genomic association and prediction integrated tool (GAPIT) version 3.0 [97] using a compressed mixed linear model (MLM) including principal component analysis (PCA) components (PC) as fixed effect covariates and kinship matrix as random variance. For GWAS, each category in each nominal variable (*LeS, FPS, EFC* and *SPS*) was transformed to a binary variable. Ordinal variables were treated as quantitative variables. Only SNPs with MAF > 5% were used in the GWAS, PCA, and kinship calculations. The optimal PC needed to control for population structure of each trait in each trial was determined using a Bayesian information criterion (BIC) included in GAPIT. The kinship matrix was calculated using all SNPs in the panel by an additive efficient mixed model (EMMA) algorithm implemented in GAPIT. The direction of the effect was calculated with respect to the minor allele.

The meta-analysis was performed using a sample-size based approach, using corrected p-values and a fixed effect model in METAL [98]. p-values at individual loci for each trait in each GWAS panel were corrected for the genomic control inflation factor (λ), which is defined as the median of the observed χ^2^ test statistics divided by the expected median of the corresponding χ^2^ distribution at individual loci for each trait [35] in each GWAS panel (Dataset S12). SNPs associations examined in at least two GWAS panels achieving genome-wide statistical Bonferroni corrected P-value threshold *P* < 10^−5^ were considered to have significant evidence of association.

### Definition of candidate gene region and associated locus

A candidate region carrying lead SNP (meta-analysis significant SNP) was defined based on a window size length of intra-chromosomal LD decay (Supplementary Table 4) and centred around the lead SNP. Genes mapping within the boundaries defined by the intra-chromosomal LD were considered a potential candidate gene. Within the region, the closest gene to the lead SNP with known function related with the trait, or interacting within the STRING network [99] to a gene with known function related with the trait, was chosen as a candidate proximal gene. If no gene was found within the region, the gene closest to the lead SNP was selected. For some SNPs, a distal gene was annotated as candidate. Candidate distal genes were those genes within 1 Mb outside the candidate region with a known function related to the trait. Candidate genes were identified and annotated from the tomato reference genome version 2.5.

Loci were then defined by merging lead SNP candidate regions that physically overlapped and found in an associated LD block as follows: for each lead SNP, regional LD was calculated within the candidate gene region using the imputed SNP matrix. LD blocks were defined for SNPs with *r*^2^ higher than the baseline *r*^2^ value 0.1349. Overlapping LD blocks or close (within 100Kb) were combined to conform an associated loci using beddtools [100].

### Identification of previously reported loci

Genomic regions including each lead SNP ± 1 Mb were compared against positions of previously published QTLs, GWAS analyses, and genes cloned with known effects on natural variability (Dataset S13). Those trait associations previously reported for the same trait or a related trait inside or overlapping with the defined region were annotated in Dataset S9.

### Integration of phenotypic and genotypic data

Phenotypic and genotypic data integration was performed using MFA-HCPC analysis as described above but including the genotypic matrix of associated SNPs as tenth class of qualitative variables. The number of clusters was selected to maximize the number of accessions per cluster classified previously in each phenocluster. Those allele/SNP combinations with a frequency higher than 60% and with an enrichment p-value<0.0001 were considered representative of a cluster.

### Fuji-plot

GWAS Meta-analyses, linkage block data, candidate genes, and the most prevalent genotype in each phenocluster, were summarized using the Fuji-plot script developed by [101] but with some modifications in data track and ideogram to include allele prevalence and LD-blocks.

## Acknowledgments

We thank Universitat Illes Balears, the Greek Gene Bank (GGB-NAGREF), Università degli Studi Mediterranea Reggio Calabria, the CRB-Leg (INRA-GAFL)”, the Genebank of CNR-IBBR (Bari, Italy) and ARCA 2010 for seed sharing. CNR-IBBR also acknowledges the seed donors, the Leibniz Institute of Plant Genetics and Crop Plant Research, Maria Cristina Patané (CNR-IBE, Catania, Italy) and La Semiorto Sementi SRL, as well as Mrs. Roberta Nurcato for technical assistance. IBMCP-UPV acknowledges Maurizio Calduch (ALCALAX) for technical assistance and Mario Fon for English grammar editing. This work was supported by European Commission H2020 research and innovation program through TRADITOM grant agreement No.634561, G2P-SOL, grant agreement No. 677379, and HARNESSTOM grant agreement No. 101000716. Clara Pons and Mariola Plazas are grateful to Spanish Ministerio de Ciencia e Innovación for postdoctoral grants FJCI-2016-29118 and IJC2019-039091-I/AEI/10.13039/501100011033; Joan Casals to a Serra Húnter Fellow at Universistat Politècnica de Catalunya.

## Author Contributions

A.G and A. J. M conceived and coordinated the study. M.J.D, S.S, J.C and J.P designed the Phenotypic characterization kit. M.C, A.M, A.K, M.J.D, S.S, J.C, S.G and St.G selected and provided seeds from genebanks and seed collections. C.P, J.C, S.P, L.F, A.R, J.L.R, A.R, M.R.F, M.P, At.K, K.N, M.E.P, M.S, M.C, J.F, S.S, M.J.D, S.G, A.M, G.G, St.G and D.Z phenotyped the collection. C.P, J.C, S.G and A.M curated phenotypic data. C.P sampled plant material for DNA, performed DNA isolation for GBS assay. P.Z, J.B, J.Cñ, M.B, R.F processed GBS data and developed the GBS marker dataset. CP performed all analyses, prepared all figures and drafted the manuscript with A.G and A. J. M. All authors participated in the final manuscript revising and approved the version to be published.

## Data Availability

GBS data raw are available in SRA SRA (https://www.ncbi.nlm.nih.gov/sra)under accession numbers PRJNA722111 and PRJNA774172. Datasets 6 and 7 are available at Zenodo (https://zenodo.org/) under DOI: https://doi.org/10.5281/zenodo.5720772

## Competing Interest Statement

The authors declare that they have no conflicts of interest.

## Supplementary information

Supplementary data is available at *Horticulture Research* online.

## Supplementary Material

Web_Material_uhac112
