# Supplementary material for: Atlas of phenotypic, genotypic and geographical diversity present in the European traditional tomato
Source: Hortic Res. 2022 May 17;9:uhac112. doi: 10.1093/hr/uhac112 (PMC9252105; doi:10.1093/hr/uhac112)
Supplement: Web_Material_uhac112 [file web_material_uhac112.zip › HORTRES_SI_ClaraPons.docx]

# Supplementary Information for

# **Atlas of phenotypic, genotypic and geographical diversity present in the European traditional tomato**

Clara Pons^1,2^, Joan Casals^3^, Samuela Palombieri^4^, Lilian Fontanet^5^, Alessandro Riccini^6^, Jose Luis Rambla^2^, Alessandra Ruggiero^4^, Maria del Rosario Figás^1^, Mariola Plazas^1,2^, Athanasios Koukounaras^7^, Maurizio E. Picarella^6^, Maria Sulli^8^, Josef Fisher^9^, Peio Ziarsolo^1^, Jose Blanca^1^, Joaquin Cañizares^1^, Maria Cammareri^4^, Antonella Vitiello^4^, Giorgia Batelli^4^,Angelos Kanellis^10,^ Matthijs Brouwer^11^, Richard Finkers^11^, Konstantinos Nikoloudis^12^, Salvador Soler^1^, Giovanni Giuliano^8^, Stefania Grillo^4^, Silvana Grandillo^4^, Dani Zamir^9^ Andrea Mazzucato^6^, Mathilde Causse^5^, Maria José Díez^1^, Jaime Prohens^1^, Antonio Jose Monforte*^2^, Antonio Granell*^2^

* Antonio Jose Monforte.

Email: amonforte@ibmcp.upv.es

* Antonio Granell.

Email: agranell@ibmcp.upv.es

**This PDF file includes:**

Supplementary results and Supplementary methods

Figures S1 to S17

Tables S1 to S6

Legends for Datasets S1 to S13

SI References

**Other supplementary materials for this manuscript, and provided in separate files, include the following:**

Datasets S1 to S13

# Supplementary results

## Correlation analysis

Clustering of the traits based on correlation analysis (Figure 2c) defined four groups, with more than four traits each, showing moderate or strong correlations (R^2^=0.25-1, p-value<0.01). In most cases, the traits correlated within a trait category. Cluster I contained the largest number of traits, and it was mainly composed by fruit size and shape traits. Within Cluster I, the correlated traits included all shape homogeneity traits (*fas*, *lob, rec, ell,* and *cir*), most traits related to fruit size (*fw, lcn, ar, per, H.curved, H.max, H.mid, W.max, W.mid*) and proximal fruit end shape (*pan.macro, piar, psh, FShS and pblk*), and some related to fruit asymmetry (*ver and tri*), distal fruit end shape (*dan.macro*, *rce* and *sps*), internal fruit structure (*puf, par* and *ptk*), seed position (*dec*), fruit external colour (*GhS*) and fruit internal colour (*fic.b*). This indicates that in the traditional European tomato gene pool, bigger fruit were associated to uneven triangular shapes, with pronounced and dark green shoulders, irregular and intended calix end and thick pericarps. Furthermore, Cluster I contained the majority of traits describing plant architecture (*GH, ADI, Hu1I* and *TH*), reflecting that indeterminate and tall plants would most likely have big and uneven shaped fruits with green shoulders. Cluster II correlated most of the external (*fec.a*, fec.b** and *fec.L**) and internal fruit colour attributes (*fic. Luminosity, fic.Red, Fic. Green, Fic.L, Fic.H, fic. b* and, *fic.C*) with fruit quality (*SSC* and *firm*) and total number of inflorescences (*tni*). Thus, fruit of traditional European tomatoes with intense, dark and bright internal and external colours were more prone to be firm, with a higher *SSC*, and to derive from plants producing a large number of inflorescences. Within Cluster III, we found correlations between shape indexes (*fps*, *fse.I, fse.II, Fse.curved and fsi*), pericarp area ratio (*per*), distal shape area index (desi) and eccentricity (*ecc*), which indicates that most traditional European elongated fruits tended to have a large part of the fruit occupied by the pericarp and seeds and, with a high probability, a pointed fruit end. Within Cluster IV, correlations were found between obovoid-related traits [obovoid-ovoid index (*osi*), width widest position (*ww*), distal shape traits such as distal angle micro (*dan.micro*) and distal fruit blockiness (*dblk*)] and leaf border (*leB*), indicating that it was highly probable to find larger fruit widths in obovoid fruits with indented distal shape. Furthermore, it indicated that plants with strongly-serrated leaf borders were more likely to have obovoid fruits. Comparisons between clusters indicated that traits in Clusters III and IV (except for *leB*) presented moderately positive correlations with Cluster II, indicating that within the traditional European tomato, elongated and obovoid fruits were more likely to have intense, dark and bright colours both inside and outside, and were more prone to be firm and to have a higher SSC. Furthermore, most of the traits in Clusters II, III and IV, with the exception of *lob*, *dsblk* and *fic.Blue*, presented moderate to strong negative correlations with Cluster I.

## Phenotypic description of traditional tomato phenoclusters

Detailed data for variables with significant contribution in defining the typical accessions in each phenocluster were assessed at p-value <10^-4^ in v-test enrichment (Figure 3, Supplementary Table 2 and 3).

C**luster C1** included 66 accessions**,** with 97% of Italian origin coming from Southern Italy. C1 contained most of accessions classified genetically in ref. [1] as *lsl piennolo* (94% 1) and *lsl-heart* (71%). C1 accessions had predominantly heart-shaped fruit (~80% of the accessions in the C1) and very small biloculated fruits (*fw* =28 g and lc=2.23), used mainly for LSL (~80%). All the fruits in C1 were not fasciated (100%), ended with a pointed tip, and had a d*otted* shape of pistil scar (100%). In addition, most of the fruits had flat light green shoulders (*FShS* 77.3% and *GSh* 69.9%) and weak ribbing at the calyx end (78.8%). Typical fruits in C1 had an intense red colour (high *fec.a** = 24.4 and *fec.b*=24.63)*, good *SSC* content (6.5 °*Brix*) and medium firmness (*firm* = 53.6). Plant architecture also contributed to define accessions into C1: 76% of the accessions in C1 had leaves with undulate leaf borders and the typical plant had on average 8.9 inflorescences, with a distance of 18.6 cm between inflorescences.

**Cluster C2** comprised 254 accessions which were enriched in Italian accessions **(**67%) mainly from Southern and Central Italy, with a few accessions from Northern of Italy. Furthermore, C2 also included accessions from Greece (19%) and Spain (9%) in lesser proportions, while French accessions were not present in this cluster. C2 contained all the accessions genetically classified as *tondo_picolo*, *lemonia* and *baleraic_cherry (only one)*, most of *ita_ellipsoid* (89%) and *ita_small* (67%), as well as almost half of the *lsl_da_serbo* (52%) and San_Marzano (46%) ones. Other genetic groups with a representation of less than 30% were also included in C2 (Fig 3C). C2 accessions were characterized by ellipsoid (23.2%), obovoid (18%), rectangular (16%) and round fruits (35%) with a dotted-shape pistil scar (93.3%), no fasciation (94.5%) and used mostly for processing or fresh/processing (18.9% and 26.8% respectively). Typical fruits in C2 showed flat and uniform green shoulders (56.3% and 26%, respectively), and almost half of the C2 accessions had a very weak ribbing at the calyx. C2 fruits were bi or tri-loculated cocktail size (*fw*=48g and *lcn*=2.6), characterized by a bright red colour outside and inside the fruits (84% red and high *fic.a, fec.b, fec.L, fic. H, fic.b, fic.C and fic.L*) with fair SSC content (5.63 °*Brix)* and medium firmness (*firm*=50.6). Regarding plant architecture, C2 accessions were mostly indeterminate but with a relative rich presence of determinate or semi-determinate growth habit (13% and 16.5% respectively) and presented a total height of 155 cm, with 8.2 inflorescences and a distance of 17cm between inflorescences.

C**luster C3 (**69 accessions) contained mostly Italian accessions (60%), predominantly from Southern and Central Italy, and to a lesser degree, from Eastern and Southern Spain (28%), and France (3%). Only two genetic classes were represented in this cluster: *pimiento (*94%), and *san_marzano (*54% of this group is in C3). This group was essentially represented by long fruits (92.7% of accessions and *fse.I, fse.II, fsi* and *fse. Curved* ~2) with a dotted shape pistil scar (98.5%), used both for fresh market and processing (85%). Accessions in C3 were enriched in fruits with flat shoulders (46%) and intermediate puffiness (51%), and had mostly bi- and tri-loculated medium sized fruits (*fw*=105g and *lcn*=2.6) with a dark red external colour (high *fec.a** and low *fec.L**).

**Cluster C4** was composed by 197 accessions mostly from Spain (94%), coming from the Mediterranean area and mainly composed by accessions that were genetically classified as *lsl penjar cat* (82.35%), *lsl penjar vlc* (87%) and *lsl ramellet* (89%). C4 tomatoes, used principally for LSL (92%), were enriched in pink tomatoes (~78% having a pink colour and colourless skin) and round-shaped fruits (35%). Almost all fruits in C4 were not fasciated and had a dotted shape pistil scar. Furthermore, around 75% of the C4 accessions presented medium green shoulders and weak ribbing at the calyx end. Fruits in C4 were on average 59 g in weight, and had 3.5 locules, good SSC content (6° *Brix),* and a medium firmness (*firm*= 54.2).

**Cluster C5 (**210 accessions), was mainly composed by accessions from Greece and France, with these countries representing 44% and 26% of the accessions in this cluster, respectively, with the *grc* and *palosanto pometa* genetic groups the best represented, with 71% and 66% of accessions. Another 8 genetic groups with less than 25% accessions contributed to C5. This group was composed by tomatoes that were commonly consumed fresh (90%). C5 fruits were predominantly round (42%) or flat (47%), and not fasciated (88.6%). Furthermore, 46% of the C5 fruits presented slightly depressed shoulders, 17% dark green shoulders, and 31% and 23% stellate and dotted shape pistil scars. These fruits were mostly (83%) pale red-orange (high *fec.b* and *fec.L*) and bright green inside (*low fic.a* and high *fic.b, fic.L, fic.H, fic.Blue, fic.Green, fic.luminosity*), medium-sized (*fw*=175.5 g), with a thick pericarp (*ptk*=1.3 and *ptk.R*=13) and on average had fair SSC (4.7° Brix). The plants in this cluster were on average 177 cm in height with 8.3 inflorescences per plant, with the first flower found at 27cm on average.

C**luster C6** (194 accessions) included mostly Italian accessions (70% of the accessions from this cluster), and a representation of French, Spanish and Greek accessions. From the genetics point of view, C6 was the most diverse cluster with 18 genetic groups, with this cluster containing 100% of *scatolone_di_bolsena* and *spagnoletta* and the 66% of *marmande*, 85% of *bell pepper* and 54% of *liguria* types. This cluster was represented by fruits that were preferentially used for fresh market (61.8%), with a predominant flat shape (79%), but enriched in bell pepper-shaped fruits (3.6%). C6 accessions were enriched in fruits with low and intermediate fasciation (32% and 43%, respectively), moderately and strongly depressed shoulders (49% and 30%, respectively), intermediate and strong ribbing at the calyx end (44% and 23%, respectively). Furthermore, enrichment analysis indicated that 44% of the fruits had an irregular shaped pistil scar, 11% presented severe puffiness, and 26 uniform green shoulders. The fruits in C6 were medium in size (*fw* = 191g) on average, had 8.65 locules, and a large and thick pericarp (*par* = 16 and *ptk*= 1.5). Typical C6 plants were on average 158 cm tall with 6.6 inflorescences per plant, with the last inflorescence being at 143 cm.

C**luster C7** (77 accessions) was composed by accessions from Spain (75 %), France (16%), Italy (6%) and Greece (3%). The two most represented genetic groups were *cour de bou* (83%) and *valenciano* (78%). These oxheart shape tomatoes (93%) were basically used for fresh market (96%). In addition, 36.4% of the fruits presented low fasciation (36.4%), 57% intermediate green shoulders, and 64% and 12% had a stellate or line-shaped pistil scar, respectively. Typical fruits in C7 were large (*fw*=254g), highly loculated (*lcn* = 9.1), with fair SSC (4.2° *Brix*), and were soft (*firm*=32). C7 accessions had on average plants that were 215 cm in height with 6.4 inflorescences at a spacing of 26 cm.

C**luster C8** (132 accessions) was characterized almost completely by Spanish accessions (97%), widely distributed among all countries, with most accessions being genetically classified into three genetic groups (79% *monserrat*, 80% *muchamiel* and 67% *palosanto pometa 2*). C8 accessions were consumed fresh (99%), and had a predominant flat shape (63%), moderately to strongly depressed shoulders (51% and 36%, respectively), intermediate to severe fasciation (28% both), intermediate ribbing at the calyx end (42%) and a stellate-shaped pistil scar (46%). Cluster C8 had on average the biggest fruits of the collection (*fw*=252g and all fruit size morphological parameters with the highest values), with 8.65 locules, poor *SSC* (3.74° *Brix*), and were very soft (*firm* =26.23). Furthermore, 5% of the C8 tomatoes were brown. Typical C8 plants had a mostly indeterminate growth habit (97%) with an average total height of 214 cm, 5.8 inflorescences per plant and with the largest average distance between inflorescences (ADI 27.2 cm).

## GBS results, linkage disequilibrium and overall levels of genetic diversity

To unveil the genetic basis of the phenotypic diversity of traditional European tomato, 445 accessions were genotyped using GBS. The GBS data obtained here, along with the GBS data obtained by ref. [1] for 1,044 European accessions, were mapped together to the tomato reference genome. A total of 39 million SNPs were obtained. Approximately, 1.4 Gb of raw paired-end reads covering approximately 0.3-fold of whole genome (~ 290 Mb) was generated per accession. In total, 110,909 SNP markers in 1,316 accessions remained after filtering with overall missing rate 0.01427% (Dataset S7). To fill in gaps in some accessions, missing genotypes were imputed (genotype imputation error rate 0.0045) (Dataset S6). Out of the 110,909-high quality GBS SNPs identified in the TRADITOM collection, 106,019 SNPs were distributed across the 12 tomato chromosomes and 4,890 SNPs in scaffolds that could not be located into any of the 12 chromosomes (ch00) (Supplementary Fig.14, Dataset S6). There were, on average, 8,835 SNPs per chromosome, ranging from 6,553 SNPs (6% of total SNPs) in chromosome ch05 to 13,269 (12%) in chromosome ch01. The average distance between SNPs was 190Kb (Supplementary Table 4). However, the distance between SNPs was smaller than 350Kb for 75% of the SNP pairs, and lower than 50Kb for 25% of them (Supplementary Table 4 and Supplementary Fig.15), indicating the presence of regions with high SNP density.

The baseline r^2^ value calculated as the 95^th^ percentile of the inter-chromosomal LD distribution corresponded to r^2^ = 0.135. Intra-chromosomal r^2^ values higher than this baseline were considered as gametophytic linkage disequilibrium (LD). This analysis (Supplementary Table 4 and Supplementary Fig.15) indicated a high level of intra-chromosomal LD between markers on the same chromosomes (average LD decay=1.74x10^6^ pb), which was in the range described previously for cultivated tomato [2,3]. LD blocks ranged from 799Kb to 2280Kb lengths and involved 3% to 16% of SNPs per chromosome (Supplementary Table 4), suggesting a founder effect, strong selection, population structure, or the presence of introgressions due to traditionalization of old obsolete commercial cultivars [1].

The genome-wide nucleotide variation across traditional European tomatoes (Supplementary Table 5), indicated that the average nucleotide diversity (π) was 0.000697/kb, which is within the expected range for cultivated tomato [4,5]. The expected number of polymorphic sites per nucleotide (θ) [6] was 0.0005973/kb with 51,401 segregating sites. Tajima’s D value [7], measuring differences in the distribution of diversity relative to neutral expectations, was -2.5, indicative of abundance of rare alleles and high linkage, and is consistent with expansion after a recent bottleneck [8]. Comparisons between countries indicated similar genetic diversity parameters in all the countries (Supplementary Table 5), with France being the country where these parameters were slightly lower. A comparison of π across the genome in the overall collection and within accessions in each country (Supplementary Fig.15) indicated the presence of regions with high nucleotide diversity, indicating the suitability for genome wide association analysis

# Supplementary methods

## TRADITOM collection

Seeds of traditional European tomato accessions (Dataset S1 ), which composed the TRADITOM collection, were obtained from the GeneBanks of the Institute for the Conservation and Improvement of Valencian Agrodiversity of the Polytechnic University of Valencia (COMAV-UPV, Valencia, Spain), from the Balearic Island University (UIB, Mallorca, Spain), from the Station d`Amelioration des Plantes Maraicheres of the French National Institute for Agricultural Research, (INRA, Montfavet, France), from the Agrobiology Department Agricultural Faculty of the University of Tuscia (UNITUS, Viterbo, Italy), from the Institute of Biosciences and Bioresources of Italian National Council of Research (CNR-IBBR, Portici, Italy), from the Greek GeneBank of Agricultural Research Center of Macedonia and Thrace of the National Agricultural Research Foundation (GGB-NAGREF, Thessaloniki, Greece) and from the seed collections of the Miquel Agustí Foundation of the Polytechnic University of Catalunya (FMA-UPC, Casteldefels, Spain), from the ARCA Società Cooperativa A.r.l. (ARCA2010, Acerra, Italy), from the University of Reggio Calabria (UNIRC, Regio de Calabria, Italy), and from the Robert H. Smith Faculty of Agriculture, Food and Environment of the Hebrew University of Jerusalem (HUJI-ARO, Rehovot, Israel).

Passport descriptors were based on FAO/IPGRI/Biodiversity Multi-Crop Passport Descriptor Lists V2.1 [9] and the traditional culinary usage (Supplementary Fig.1 and Dataset S1): fresh market (FM), processing (PR), or stored and consumed after autumn storage or long shelf life (LSL). The Spanish collection was mainly composed of FM (53%) and LSL (37%) tomatoes. The French tomatoes were mainly used for fresh consumption (88%) and in less proportion for processing (8%), or both (3%). Forty-one percent of the Greek accessions were FM tomatoes, 9% PR and 13% were used for fresh consumption or/and processing. In Italy, the use was more diverse: 24% of the tomatoes were for FM, 27% tomatoes for LSL, 5% for PR, and the rest for PR or/and FR or LSL. For 30% of the Greek and Italian accessions, the traditional use was unknown.

All the seeds were sanitized with a double chemical treatment followed by a thermal treatment as follows: seeds were washed in 10% trisodium phosphate for 3 hours, followed by a washing step with 30% bleach for one hour, and then by a dry heat treatment at 79 °C for 24 hours.

## TRADITOM phenotyping kit

A standardized protocol, defining the traits and how traits should be recorded, was established for the entire consortium. Traits were phenotyped as follows:

**Qualitative traits, visually determined. Recorded by plot basis:**

1. Growth habit (*GH*). Continuous alternation between vegetative and reproductive phases: 1. determinate/ 2. semi-determinate/ 3. indeterminate.

2. Leaf shape (*LeS* ):1. regular leaf/ 2. potato leaf/ 3. double feathered

3. Leaf border (*LeB*): 1. entire/ 2. undulate/ 3. serrated/ 4. strong serrated

4. Jointless pedicel (*jp*) Presence of an abscission zone that forms as a swelling or joint on a flower pedicel: 0. absence/ 1. presence.

5. External fruit colour (*fec*) Predominant fruit epicarp colour: 1. yellow/ 2. orange/ 3. pink/ 4. red/ 5. purple/ 6. brown/ 7. green

6. Green shoulder (*GSh*): 0. uniform/ 1. light green/ 2. medium green/ 3. dark green

7. Skin colour (*SkC*). The colour of the fruit epidermis without pericarp: 1. colourless/ 2. yellow.

8. Fruit predominant shape (*fps*), using the fruit shape categories provided by Rodriguez et al. [10] : 1. flat/ 2. rectangular/ 3. ellipsoid/ 4. obovoid/ 5. round/ 6. oxheart/ 7. long/ 8. Heart/9. bell pepper

9. Fruit shoulder shape (*FShS*). The height of the depression (shoulders) around the calyx visually determined 1. Flat/ 2. Slightly depressed/ 3. Moderately depressed/ 4. Strongly depressed

10. Shape of pistil scar (*sps*). The shape of the abscission zone where the pistils detach 1. dot/ 2. stellate/ 3. linear/4. irregular

11. Fruit fasciation (*fas*). Degree of cresting: 1. not present/ 2. low/ 3. intermediate/ 4. severe

12. Puffiness appearance (*puf*) Presence of cavity between pericarp and seeds: 1. not present/ 2. low/ 3. intermediate/ 4. severe

13. Ribbing at calyx end (*rce*). Presence of lobes at the distal part of fruit: 1. very weak/ 2. weak/ 3. Intermediate/ 4. strong

**Plant structure quantitative traits.**

These traits were recorded for all plants in the plot, approximately when fruits from the third truss started to ripen.

14. Height until the first inflorescence (cm) (*Hu1I*)

15. Height until the last inflorescence (cm) (*HuLI*)

16. Total number of inflorescences (n) (*tni*)

17. Total plant height (cm) (*TH*)

18. Average distance between inflorescences (*ADI*) was calculated in cm according to:

$$ADI=\frac{HuLI-Hu1I}{Tni-1}$$

**Fruit quantitative traits**

Eight fruits from each accession were sampled from the 2nd to 4th trusses. Fruits were selected visually to represent mean fruit shape of each accession (abnormal fruits were discarded for the analysis). Fruits were harvested at the red ripe stage. The following traits were characterized using the entire fruit.

19. Fruit weight (g) (*fw*) of the fruit. Fruit weight was categorized as 0-10g = Wild, 10-30g = Cherry, 30-50g = Cocktail, 50-100g = Small, 100-200g = Medium, 200-300g = Large, 300-400g = Very large, >400 giant

20-24. External fruit colour CIELAB parameters. The CIELAB colour coordinates of fruit epicarp were recorded in an area close to the equatorial part of the fruit with a Chroma Meter CR-400/410 (Konica Minolta, Japan). Two determinations per fruit were recorded: 20. a* coordinate measuring the green-red colour intensity (*fec.a**); 21. b* coordinate, measuring the blue-yellow colour intensity (*fec.b**); 22. L, measuring colour luminosity (*fec.L**);

23. Fruit firmness (*firm*) measured in a point close to the equatorial part of the fruit with a Durofel-AGROSTA®100 USB durometer (Agro-Technologie). Two measurements per fruit were taken. Firmness was categorized as firm <30 = Very soft, 30-40 = Soft, 40-60 = Medium, 60-70 = Firm; >70 = Very firm).

For the morphometric parameters, internal fruit colour, locule number, and soluble solid content, four fruits were sliced longitudinally and the remaining four transversally to record individual fruits, based on the following variables:

24. Locule number (from the transversal section) (*lcn*)

25. Soluble solid content (*SSC*), °Brix measured using a hand-held ERMA refractometer (ERMA Instruments). Two minimum measurements per accession (mean value of the pool of 4 fruits from each section (transversal/longitudinal) or maximum of 8 measurements (individual fruit measurement) were recorded. Soluble solid content was categorized as: *SCC*<4° Brix, Poor, 4-6, Fair; 6-8, Good; > 8, Excellent

Fruit shape, size and internal fruit colour parameters were recorded using the Tomato Analyzer 4.0® software [11] according to the Tomato Analyzer user’s manual (<http://www.oardc.ohio-state.edu/vanderknaap/files/Tomato_Analyzer_3.0_Manual.pdf>) in longitudinal (minimum of 4 fruits) and transversal (minimum 4 fruits) sections. The Tomato Analyzer settings were: scanner dpi (“dots per inch”) was calibrated at 300 pixels and units in cm, upper blockiness position 0.1 and blockiness lower position 0.9, proximal and distal angle macro distance to 20% and micro distance at 3% and default concentric ellipse size at 90%. The following parameters are recorded:

Transversal section parameters were:

26. Lobedness Degree (*lob*); degree of uneven shape of the fruit in the transversal section

27. Tomato Pericarp Area (*par*); degree of uneven shape of the fruit in the transversal section

28. Tomato Pericarp Area Ratio (*par.R*); area within the pericarp inner and outer boundary measured in the transversal section

29. Tomato Pericarp Thickness (*ptk*): pericarp thickness was categorized as *ptk* <0.5 mm= very thin, 0.5-0.75 mm = Thin, 0.75-1.5 mm = Medium, 1.5-2 mm = Thick, ptk>2 = Very thick

30. Tomato Pericarp Thickness Ratio (*ptk.R*): average of four equally distributed measurements that calculate the distance between the outer and the inner pericarp boundary.

Longitudinal section parameters:

31. Maximum Height (*H.max*): The maximum vertical distance of the fruit.

32. Maximum Width (*W.max*): The maximum horizontal distance of the fruit.

33. Perimeter (*per*): Outer boundary of the fruit measured in the longitudinal section

34. Circular (*cir*): similarity of the longitudinal tomato section to a circle.

35. Curved Fruit Shape Index (*fse.curved*): The ratio of Curved Height to the width of the fruit at mid-curved-height, as measured perpendicular to the curved height line. It is a measure of how curved the fruit is.

36. Curved Height (*H.curved*): The height measured along a curved line through the fruit.

37. Width Mid-height (*W.mid*): The width measured at ½ of the fruit’s height

38. Width Widest Position (*ww*): the position of the maximum width of the fruit along the longitudinal axis

39. Distal Angle Macro (*dan.macro*): Distal fruit end shape angle at position 3% above the tip from the fruit

40. Distal Angle Micro (*dan.micro*): Distal fruit end shape angle at position 20% above the tip from the fruit

41. Distal Eccentricity (*dec*): degree of internal eccentricity at the distal end of the fruit.

42. Distal end shape index (*desi*): a measurement of the depression degree at the bottom of the fruit defined as the difference between distal end protrusion and distal indentation area. Distal end protrusion= the area of the protruded end over the total fruit area. distal indentation area= the ratio of indentation area at distal zone to total fruit area

43. Distal Fruit Blockiness (*dblk*): Distal fruit end width/mid width ratio at 90% of fruit height. A measurement of the tightening of the fruit at the distal part.

44. Eccentricity (*ecc*): the degree of internal eccentricity of the seed position in the fruit.

45. Eccentricity Area Index (*eai*): The ratio of the area of the fruit outside the ellipse to the total area of the fruit.

46. Ellipsoid (*ell*): similarity of the longitudinal tomato section to an ellipse.

47. Fruit Shape Index External I (*fse.I*): The ratio of maximum height to maximum width.

48. Fruit Shape Index External II (*fse.II*): The ratio of the height at mid-width to the width at mid-height.

49. Fruit Shape Index Internal (*fsi*): pericarp elongation index, calculated as the ratio of vertical and horizontal axes of the ellipse or circle used for eccentricity

50. Fruit Shape Triangle (*tri*): The ratio of the proximal end width to the distal end width

51. Height Mid-width (*H.mid*): The height measured at ½ of the fruit’s width.

52. Obovoid-ovoid symmetry index (*osi*): defined as the difference between *H.Asymmetry.Ob* - *H.Asymmetry.Ov*. *H.Asymmetry.Ob*=Horizontal asymmetry. Obovoid describes how asymmetric a fruit is when divided along a horizontal axis if there is more area below the horizontal axis than below*. H.Asymmetry.Ov*.= Horizontal asymmetry ovoid describes how asymmetric a fruit is when divided along a horizontal axis if there is more area above the horizontal axis than below it.

53. Proximal Angle Macro (*pan.macro*): Angle from shoulders to pedicels at position 20%.

54. Proximal Angle Micro (*pan.micro*): Angle from shoulders to pedicels at position 3%.

55. Proximal Eccentricity (*pec*): degree of internal eccentricity at the proximal end of the fruit.

56. Proximal Fruit Blockiness (*pblk*): Proximal fruit end width/mid width ratio at 10% of fruit height. A measurement of the tightening of the fruit at the proximal part.

57. Proximal Indentation Area (*piar*): a measurement of the depression degree at the top of the fruit. Expressed as the ratio of indentation area at the proximal zone to total fruit area.

58. Rectangular (*rec*): similarity of the longitudinal tomato section to a rectangle.

59. Shoulder Height (*psh*): Shoulder height relative to total fruit height.

60. V Asymmetry (*ver*): Vertical asymmetry, describes how asymmetric a fruit is when divided along a vertical axis.

61. Area (*ar*): Area defined by the outer boundary of the fruit measured in the longitudinal section

62-66. Average internal colour of fruits translated from RGB to CIELAB colour space: 62. Average a Value (*fic.a*), 63. Average b Value (*fic.b*); 64. Average Chroma (*fic.C*); 65. Average L Value (*fic.L*); 66. Average Hue (*fic.H*)

67 -70. Average internal colour measured as average pixel colour in RGB space. 67. Average Red (*fic.red*); 68. Average Blue (*fic.blue*); 69. Average Green (*fic.green*); 70. Average Luminosity (*fic.luminosity*)

#
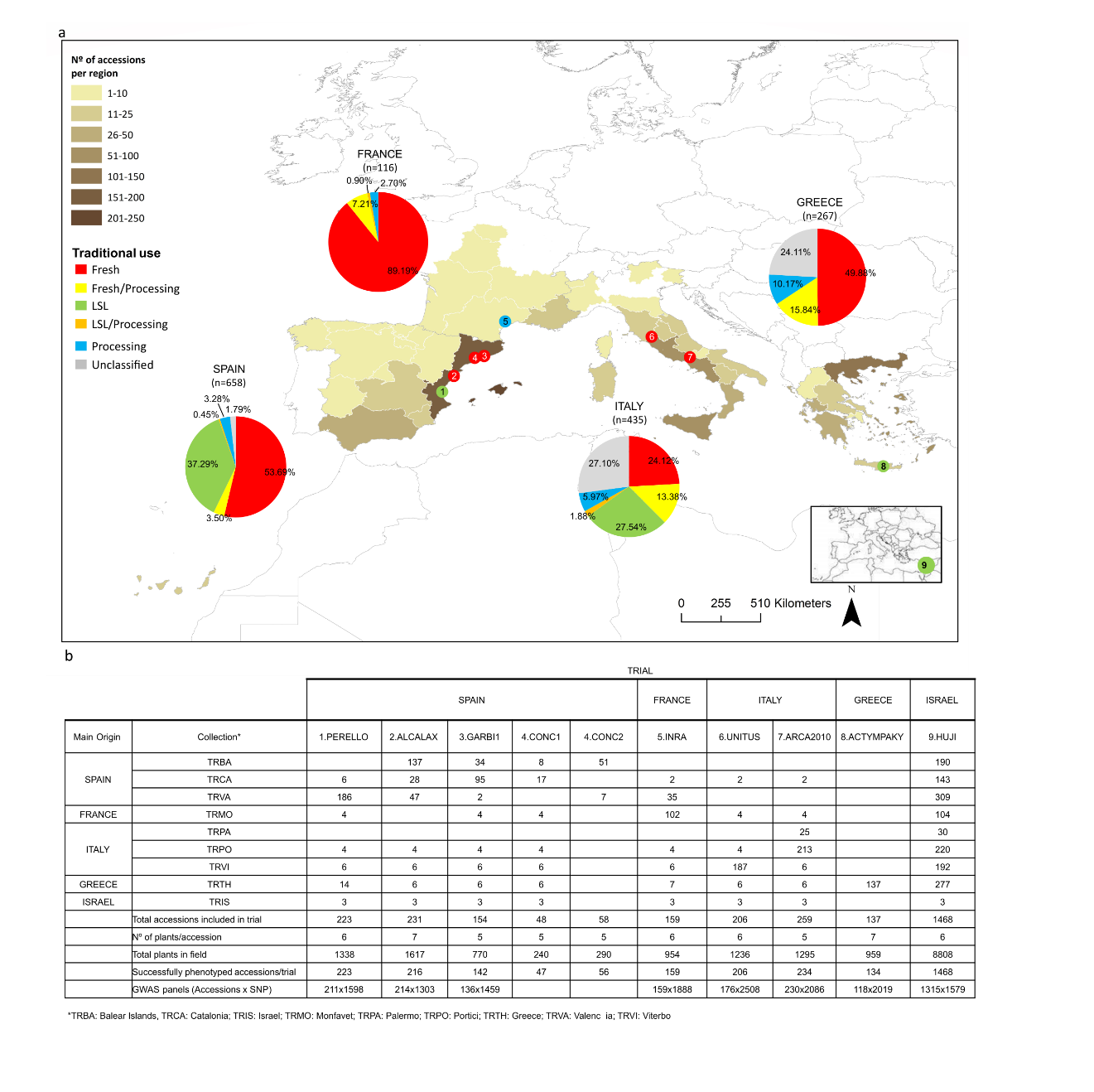
Supplementary figures

**Fig. S1. Summary of the TRADITOM collection and trials**. a) Geographical distribution of traditional tomato accessions per region and traditional use. The colour of each region in the map indicate the number of accessions coming from this region. The number of accessions from each county is also indicated. The dot number indicates the location of the trials. The colour of the dot indicates the method of cultivation: red, open field; green greenhouse and blue, mesh tunnel. The pie charts represent the proportion of different traditional uses within each country. b) Trial summary. Collections (codes are defined at the bottom), the main country of origin of accessions in each collection is indicated. For each trial is indicated the country, the location within country, the number of accessions for each collection, number of accessions and plants grown per accession and trial, number of successfully phenotyped accessions and the number of SNPs and accessions composing each GWAS panel for each trial.

**
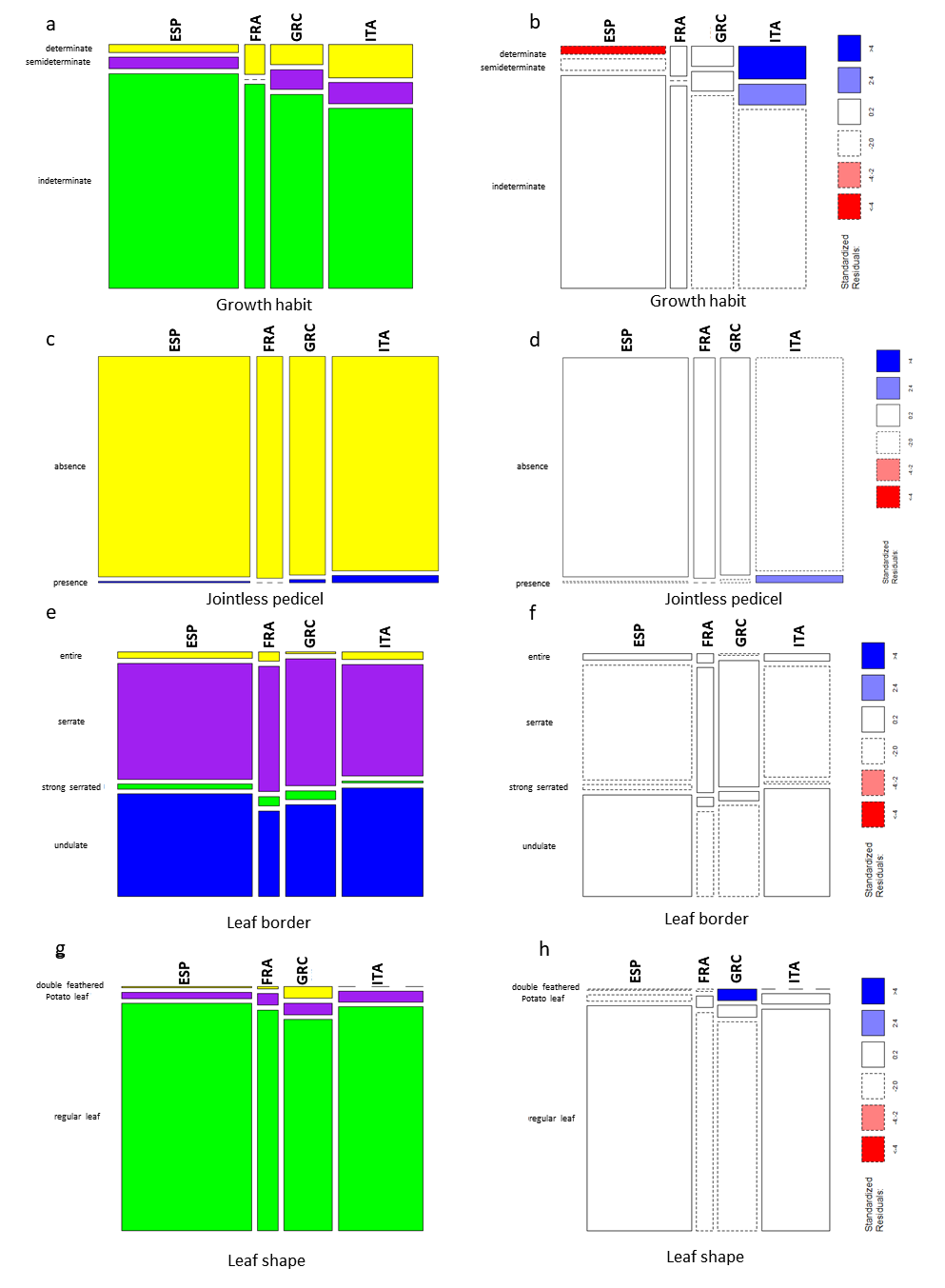
**

**
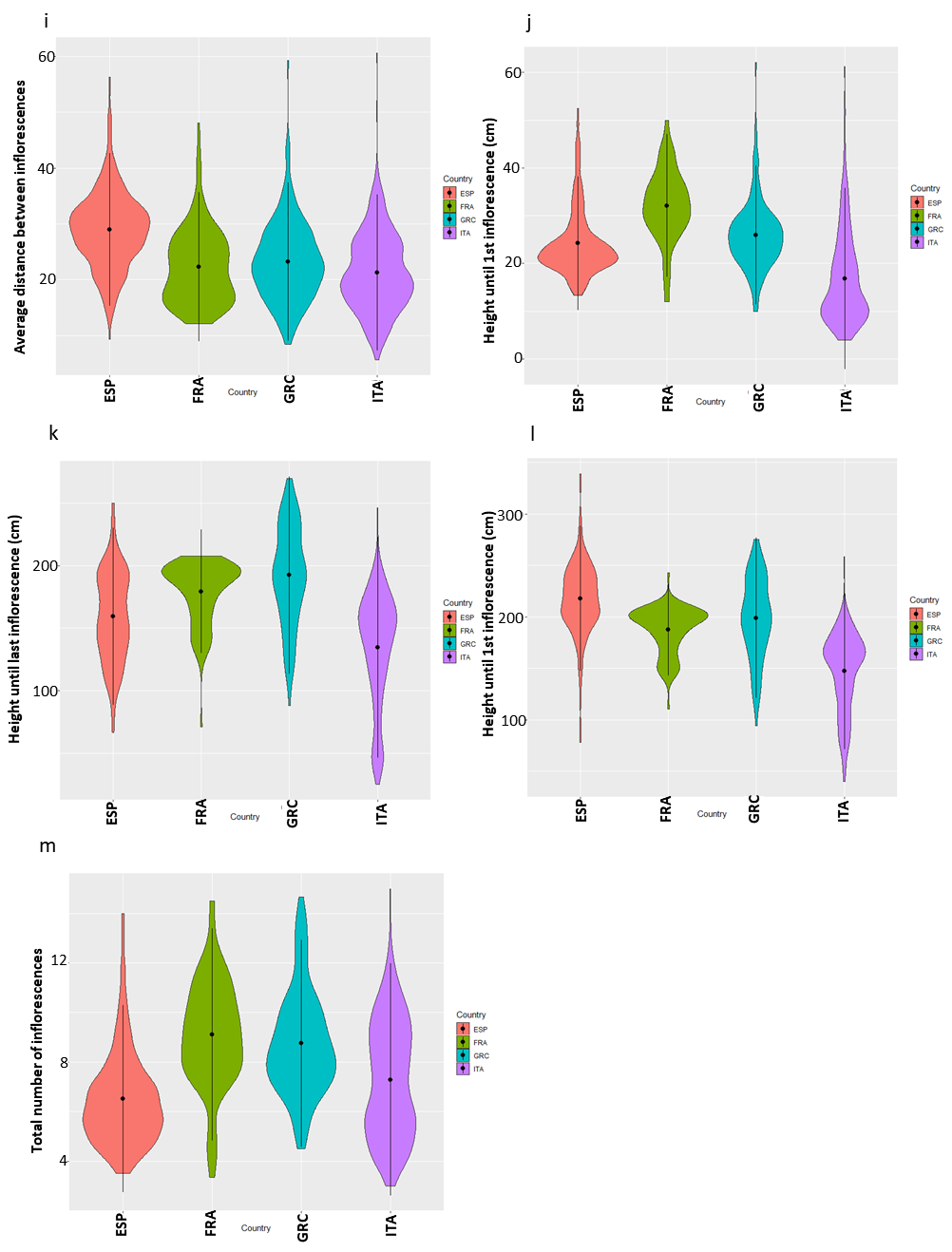
**

**Fig. S2. Distribution and enrichment of plant and inflorescence architecture traits in the different countries** of origin. a to f) Mosaic plots showing the distribution in each country of origin (a,c and e) and the enrichment (b,d and f) for growth habit (a,b), jointless pedicel (c,d), leaf border (e, f) and leaf shape (g, h). Enrichment was evaluated by departure of residuals from the expected value. Residuals with |dij|> 4 have an approximate P-value < 0.001 and |dij|> 2 have an approximate P-value < 0.05. i to m) Violin plots showing the distribution of Average distance between inflorescences (i), Height until 1st inflorescence (cm) (j), Height until last inflorescence (cm) (k), Total height (cm) (l) and Total number of inflorescences (m). The average value in violin plots is indicated by a dot.


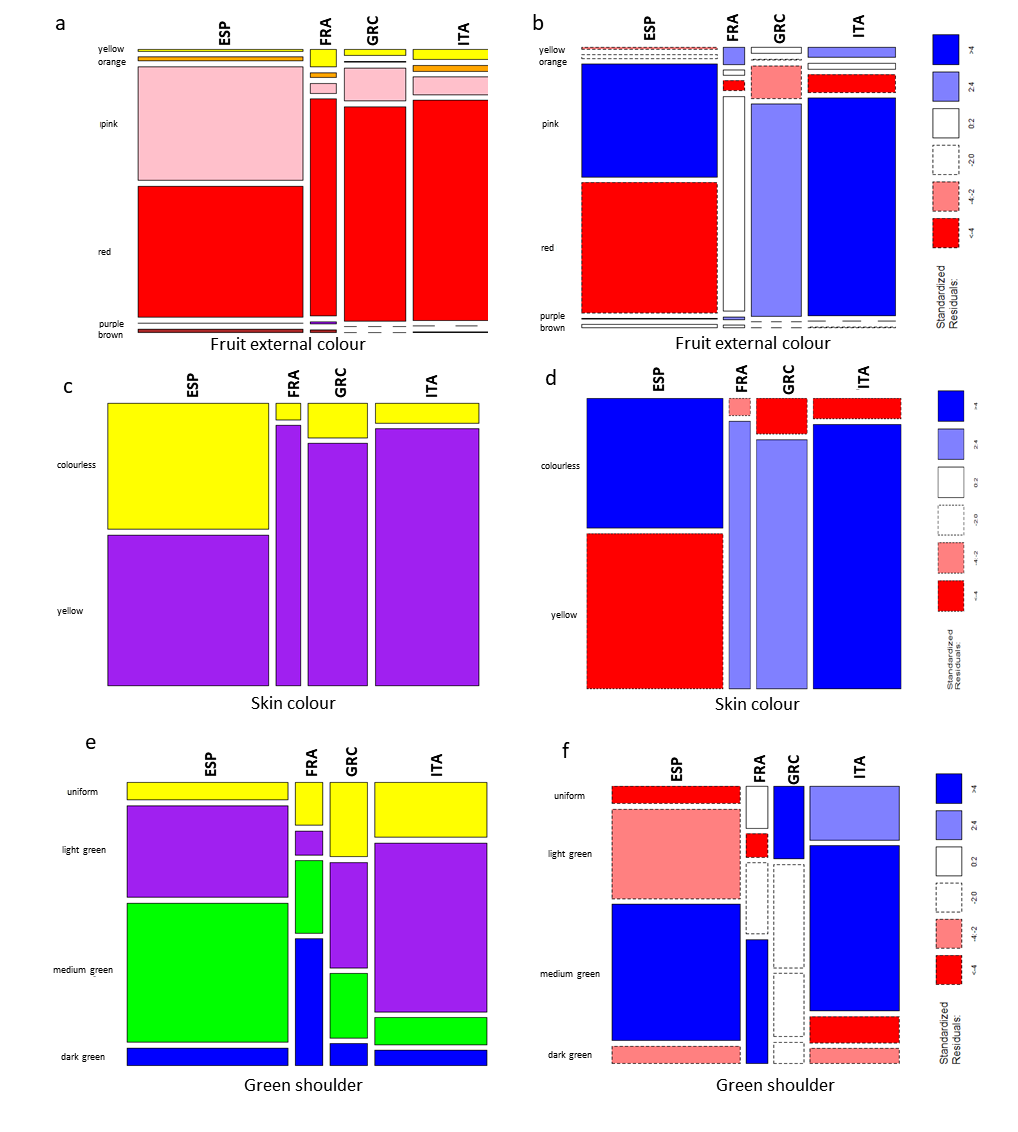


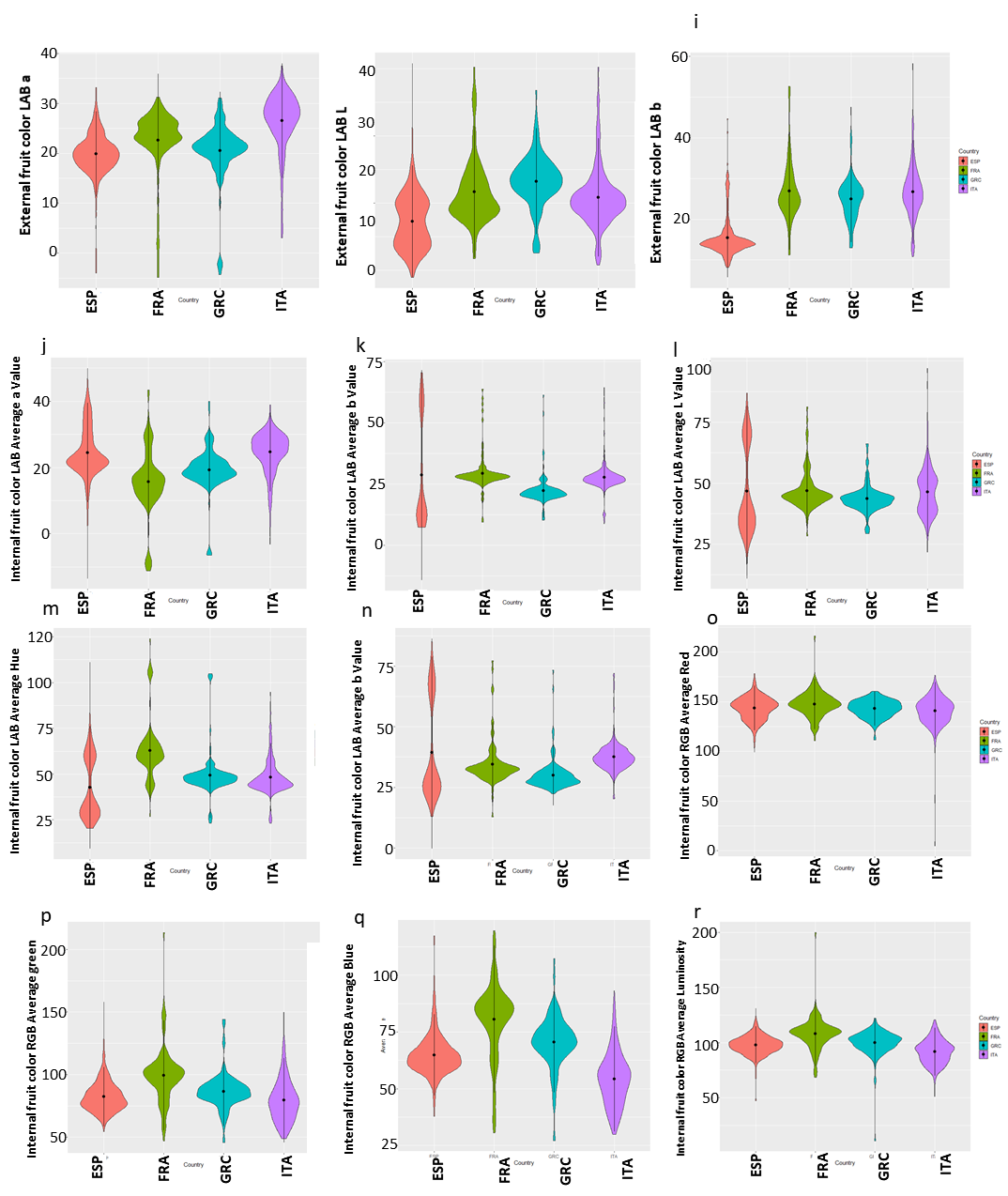


**Fig. S3.** Distribution and enrichment of fruit colour traits in the different countries of origin. a to f) Mosaic plots showing the distribution of in each country of origin (a,c and e) and the enrichment (b,d and f) for external fruit colour (a,b), skin colour (c,d) and green shoulder colour (e and f). Enrichment was evaluated by departure of residuals from the expected value. Residuals with |dij|> 4 have an approximate P-value < 0.001 and |dij|> 2 have an approximate P-value < 0.05. g to i) Violin plots showing the distribution of external fruit a*, b* and L* CIELAB coordinates. j to n) Violin plots showing the distribution of internal fruit a*, b* and L* CIELAB coordinates and Chroma and Hue angle. o to r) Violin plots showing the distribution of internal fruit RGB colour parameters (Green, red, blue and luminosity). The average value in violin plots is indicated by a dot

**
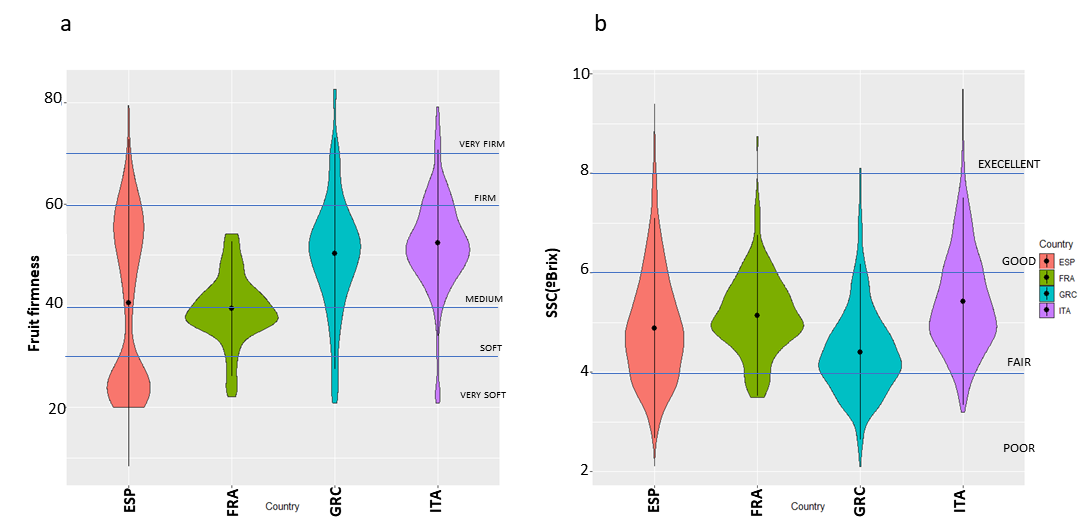
**

**Fig. S4. Distribution and enrichment of fruit quality traits in the different countries of origin.** Violin plots showing the distribution of a) Firmness and b) SSC (ºBrix) The average value is indicated by a dot. (SCC<4 ºBrix = Poor, 4-6 ºBrix = Fair; 6-8 ºBrix = Good; SSC> 8 =Excellent). Firmness category (Firm <30 = Very soft, 30-40 = Soft, 40-60 = Medium, 60-70 = Firm >70 = Very firm)

**
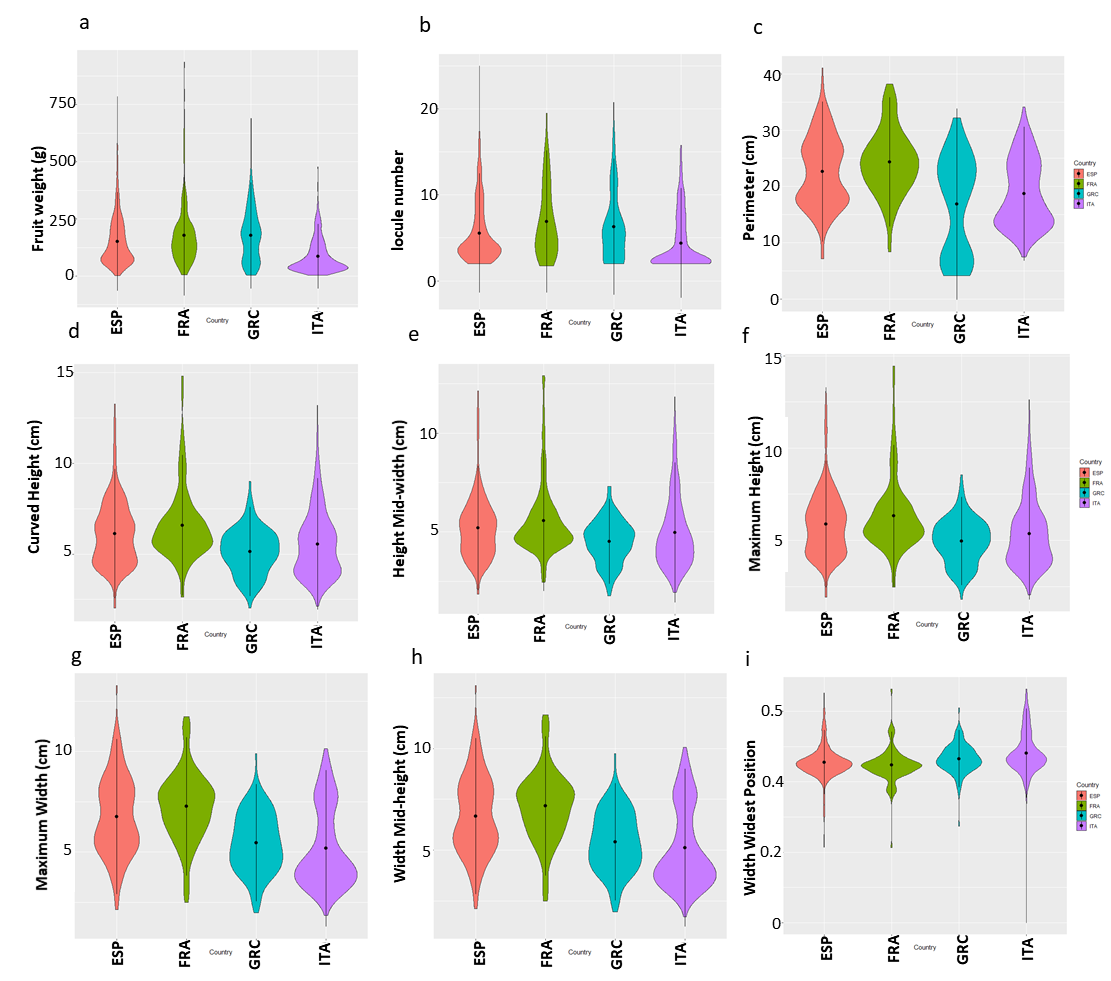
**

**Fig. S5. Distribution of fruit size traits in the different countries of origin.** Violin plots showing the distribution of a) Fruit weight, b) locule number, c) Perimeter, d) Curved height, e) Height at mid-width, f) Maximum height, g) Maximum width, h) Width at mid-height, i) Width Widest Position. The average value is indicated by a dot.


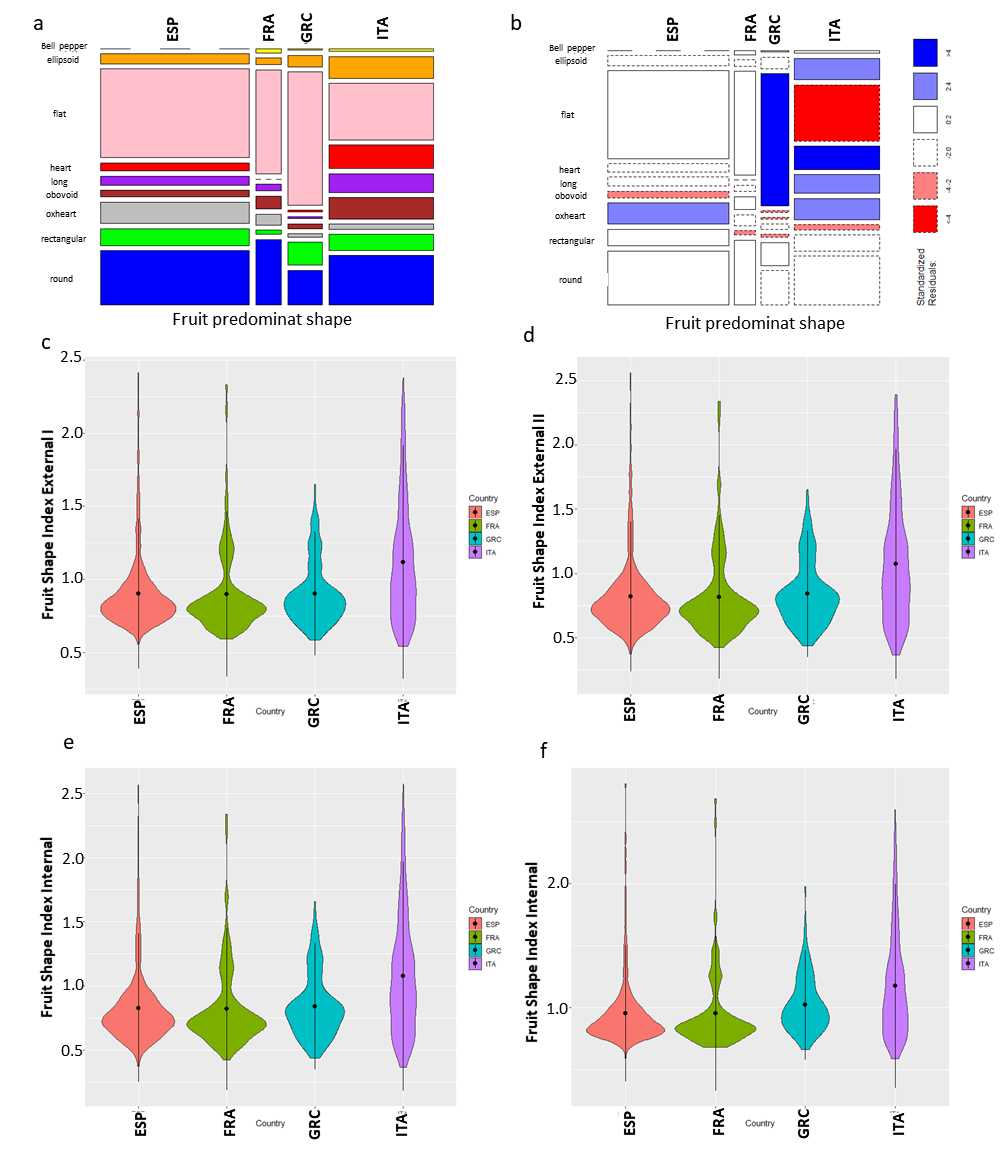


**Fig. S6. Distribution and enrichment of fruit shape traits in the different countries of origin.** Mosaic plots showing a) the distribution of fruit predominant shape in each country of origin and b) the enrichment. Enrichment was evaluated by departure of residuals from the expected value. Residuals with |dij|> 4 have an approximate P-value < 0.001 and |dij|> 2 have an approximate P-value < 0.05. c to f) Violin plots showing the distribution of fruit shape indexes. The average value in violin plots is indicated by a dot.


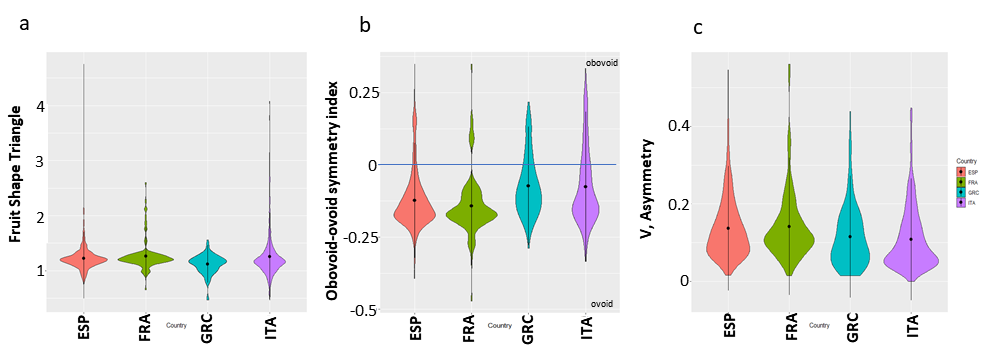


**Fig. S7. Distribution of fruit shape asymmetry traits in the different countries of origin.** Violin plots showing the distribution of a) Fruit shape triangle, b) Obovoid-ovoid index and c) vertical asymmetry. The average value is indicated by a dot.


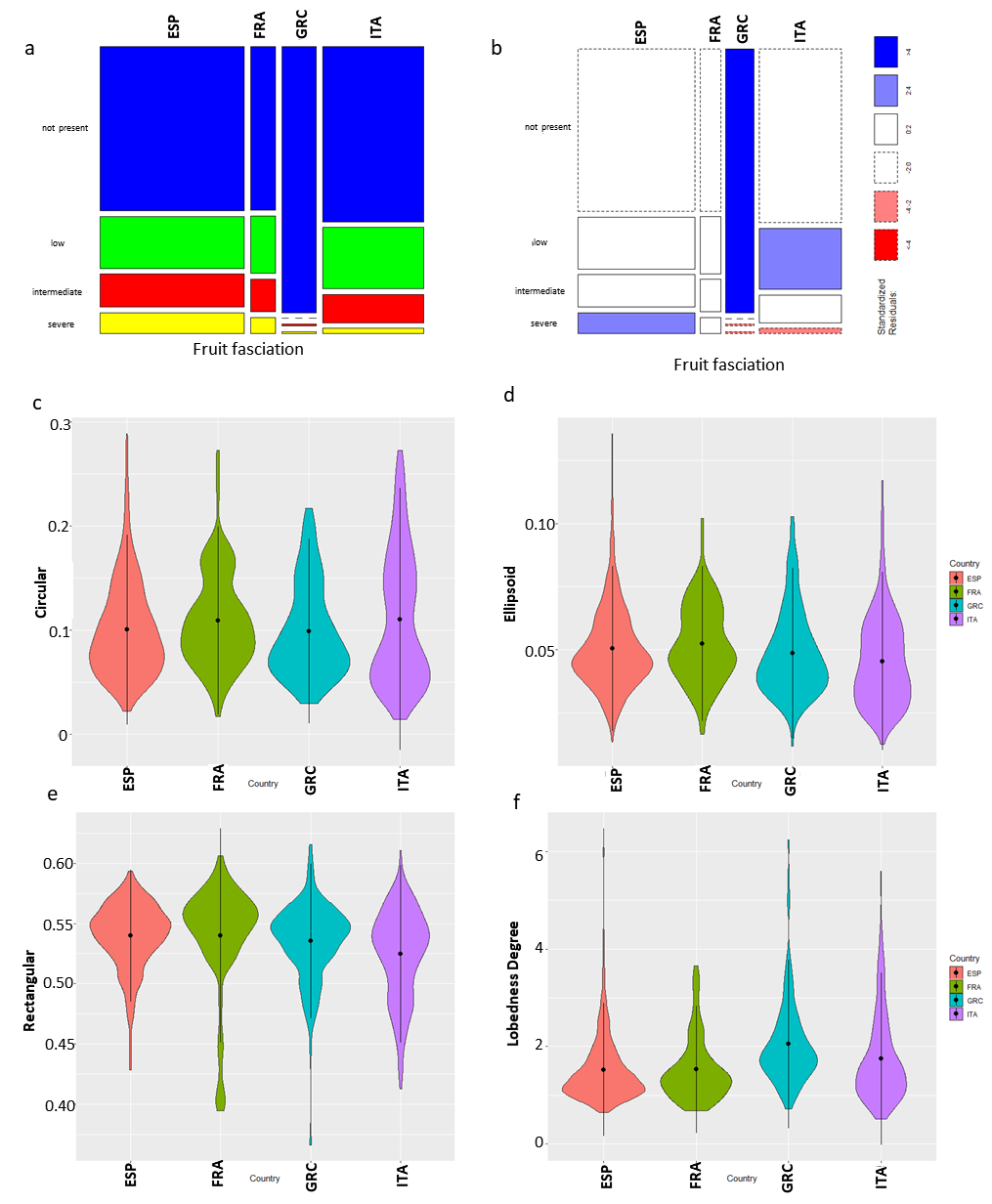


**Fig. S8. Distribution and enrichment of fruit shape homogeneity traits in the different countries of origin.** Mosaic plots showing the a) distribution of fruit fasciation in each country of origin and b) the enrichment. Enrichment was evaluated by departure of residuals from the expected value. Residuals with |dij|> 4 have an approximate P-value < 0.001 and |dij|> 2 have an approximate P-value < 0.05 c to f) Violin plots showing the distribution of departure from circular(c), ellipsoid (d) and rectangular shapes(e) and lobedness degree (f). The average value in violin plots is indicated by a dot.


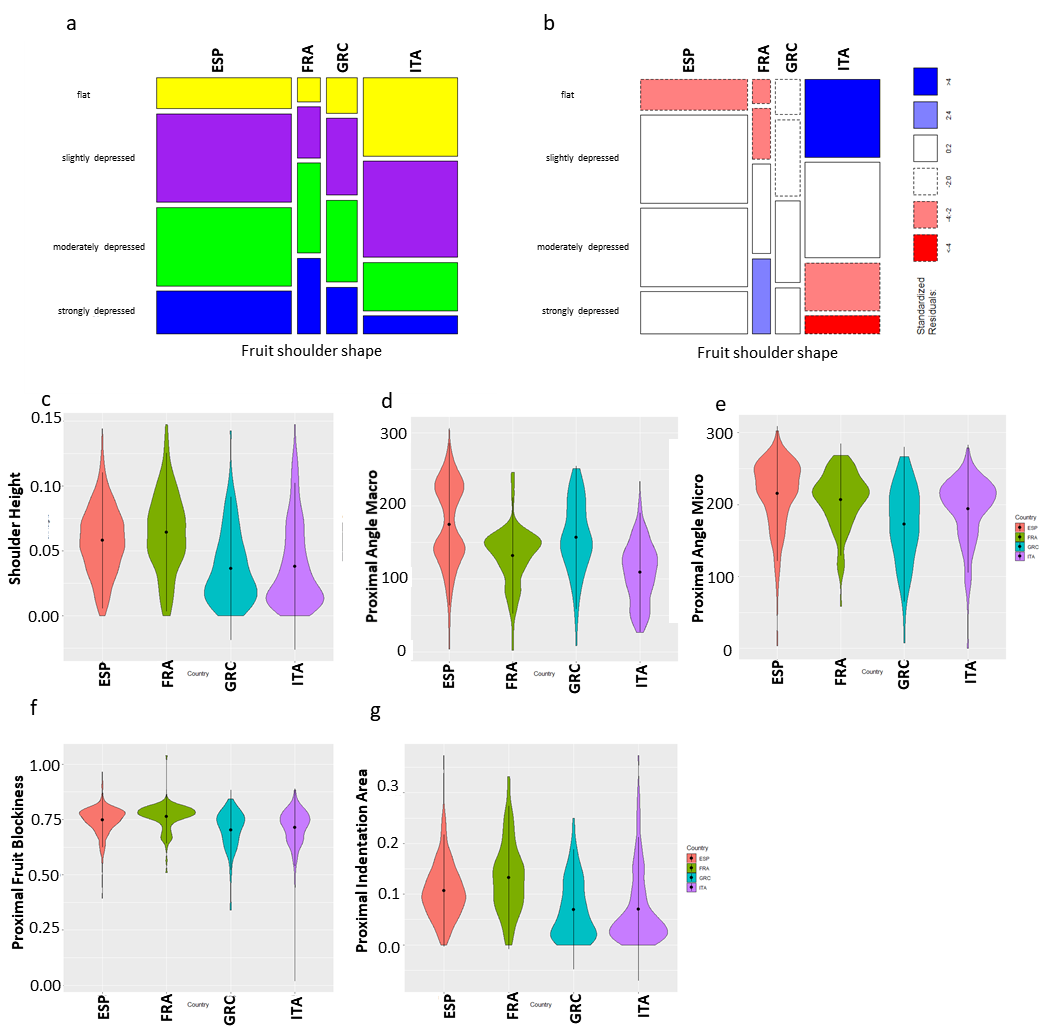


**Fig. S9. Distribution and enrichment of proximal fruit end shape traits in the different countries of origin.** Mosaic plots showing the a) distribution of fruit shoulder shape in each country of origin and b) the enrichment. Enrichment was evaluated by departure of residuals from the expected value. Residuals with |dij|> 4 have an approximate P-value < 0.001 and |dij|> 2 have an approximate P-value < 0.05. c to g) Violin plots showing the distribution of shoulder height (c), proximal angle macro at 20% (d), proximal angle micro at 3% (e), proximal blockiness (f) and (g) proximal indentation area. The average value in violin plots is indicated by a dot


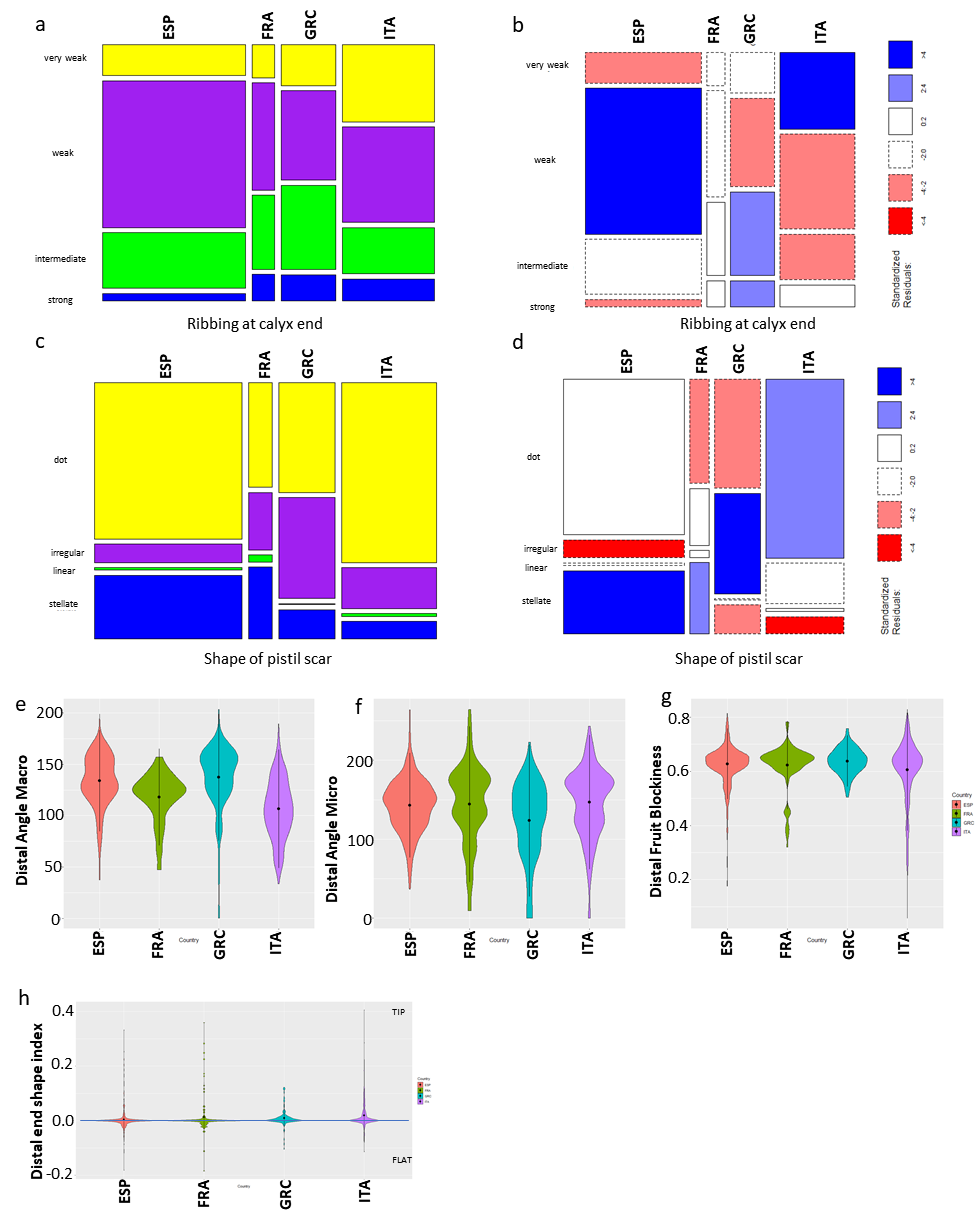


**Fig. S10. Distribution and enrichment of distal fruit end shape traits in the different countries of origin.** Mosaic plots showing the distribution in each country of origin (a, c) and the enrichment (b, d) of ribbing at calyx end (a, b) and shape of pistil scar (c, d). Enrichment was evaluated by departure of residuals from the expected value. Residuals with |dij|> 4 have an approximate P-value < 0.001 and |dij|> 2 have an approximate P-value < 0.05. e to h) Violin plots showing the distribution of distal angle macro at 20% (e), distal angle micro at 3% (f), distal fruit blockiness (g) and Distal end shape index (h). The average value in violin plots is indicated by a dot.


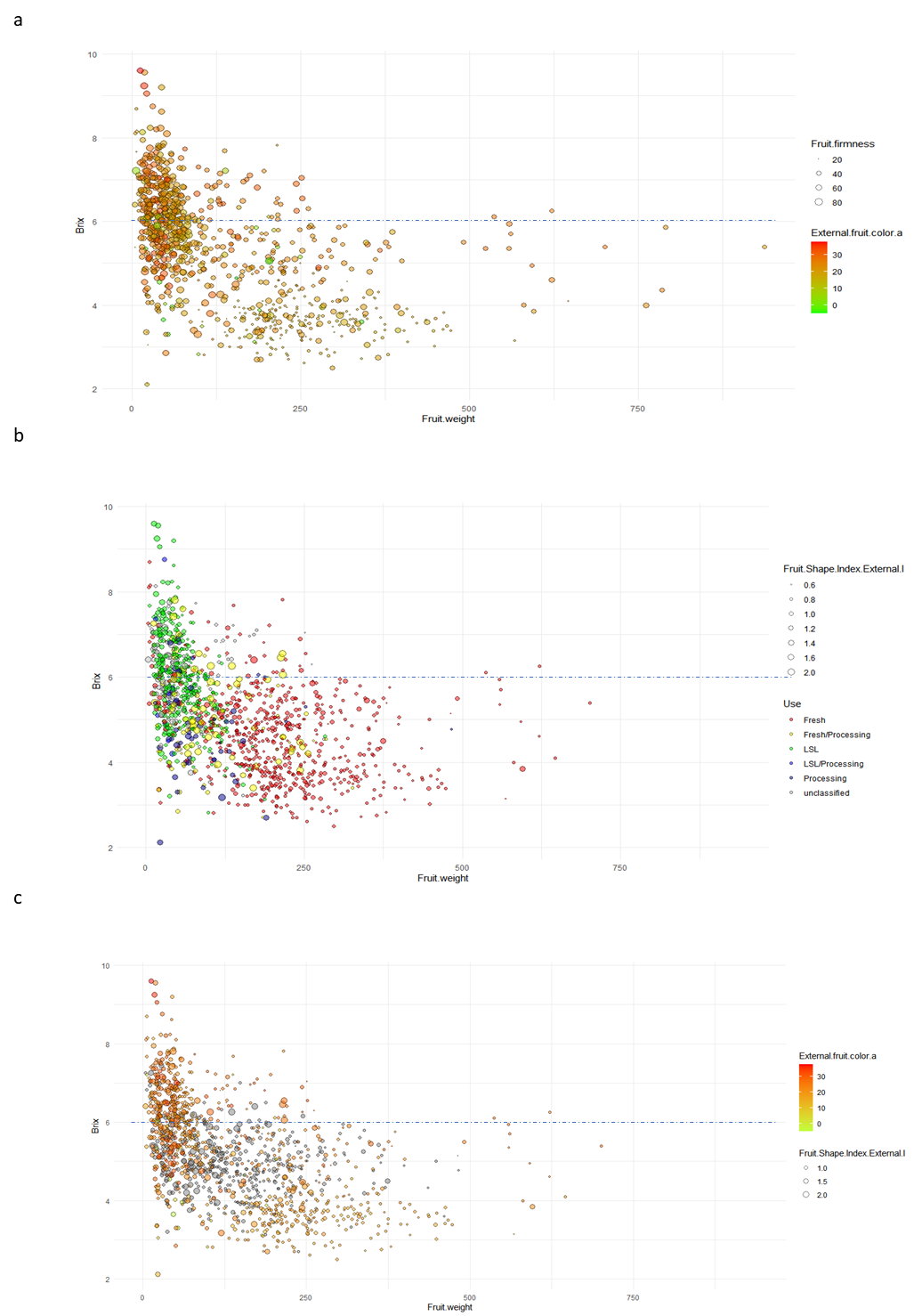


**Fig. S11. 1. 4-D bubble plot showing the relationship between fruit weight, SSC, fruit firmness, fruit colour, fruit shape index and use.** X- axis represents fruit weight in g and Y- axis represents SSC in ºBrix. a) bubble size represents firmness and bubble colour represents the external fruit colour measured as CIELAB a* coordinate. b) bubble size represents fruit shape index and bubble colour the traditional use. c) bubble size represents fruit shape index and bubble colour the external fruit colour measured as CIELAB a* coordinate.

**
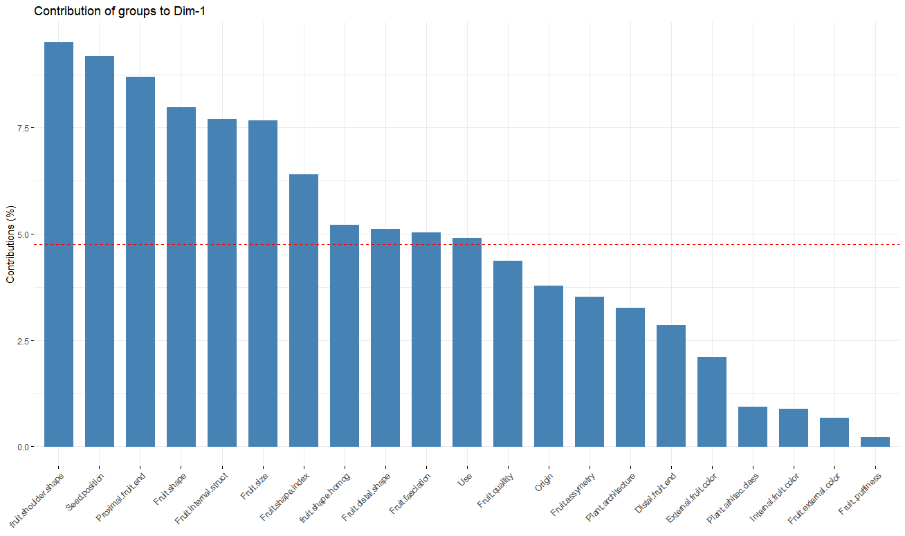
a**

**b**

**
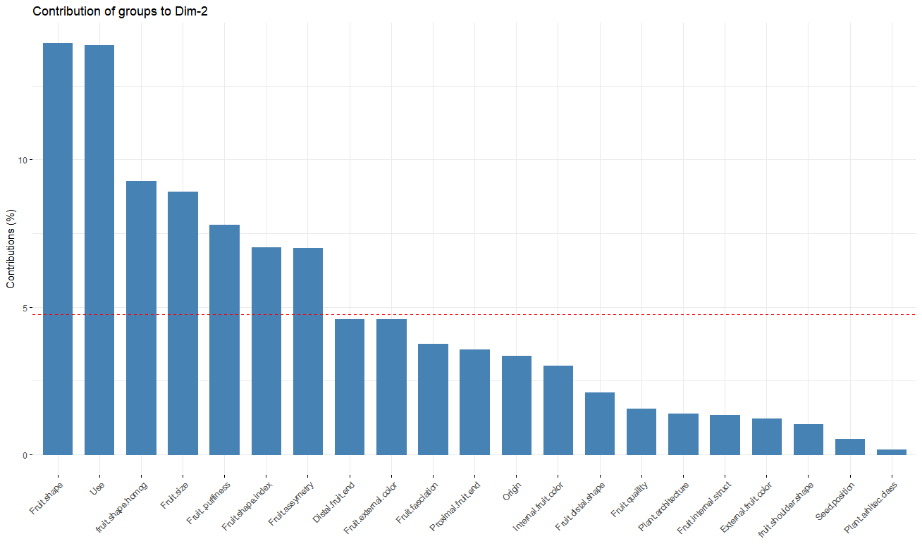

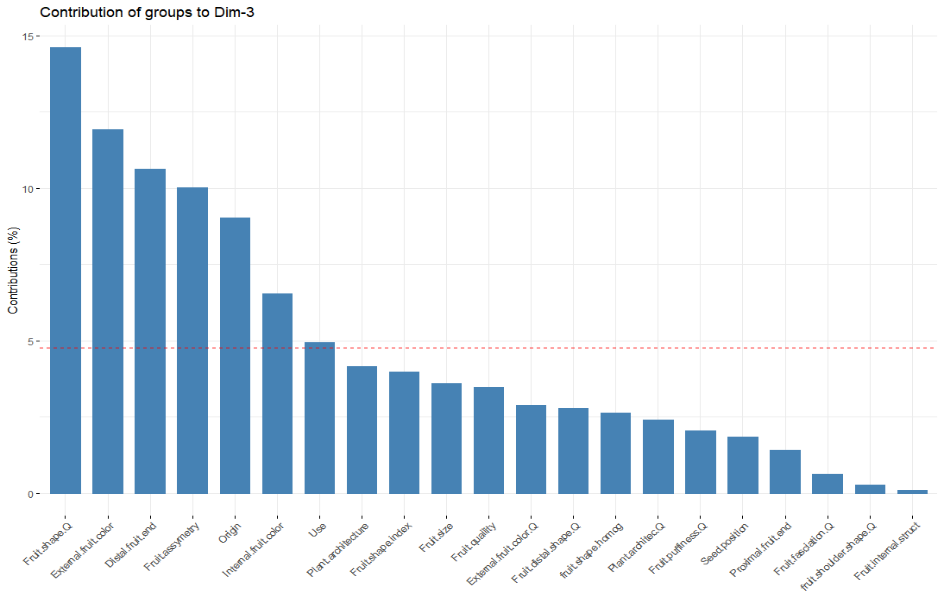
c**

**
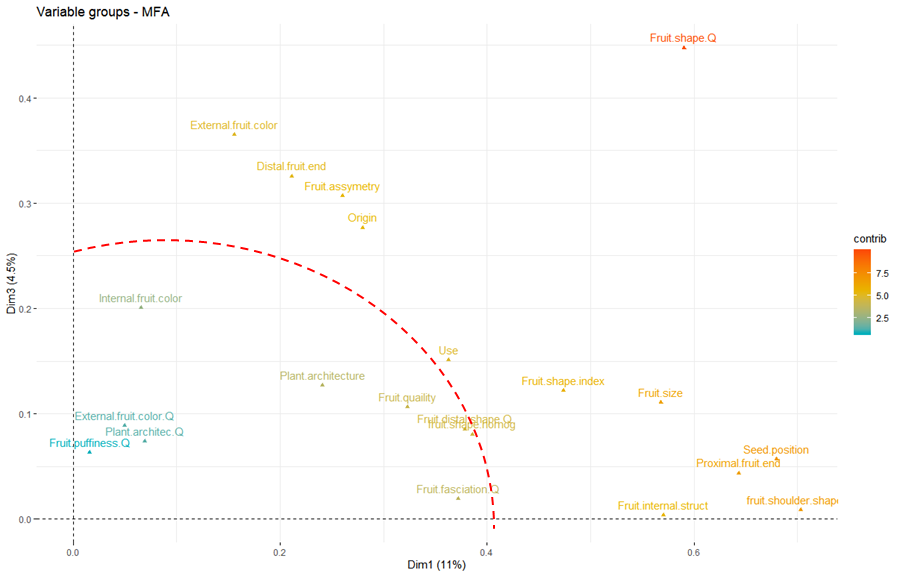

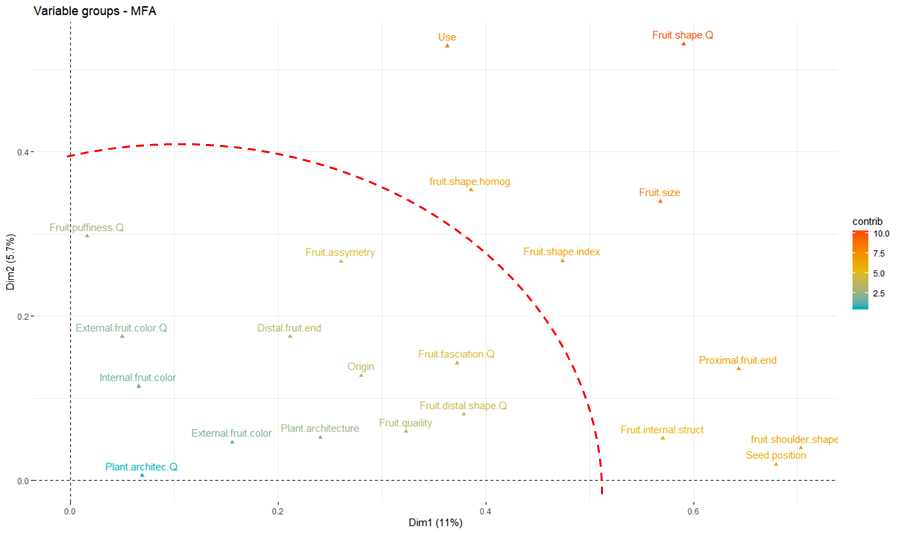
d**

**
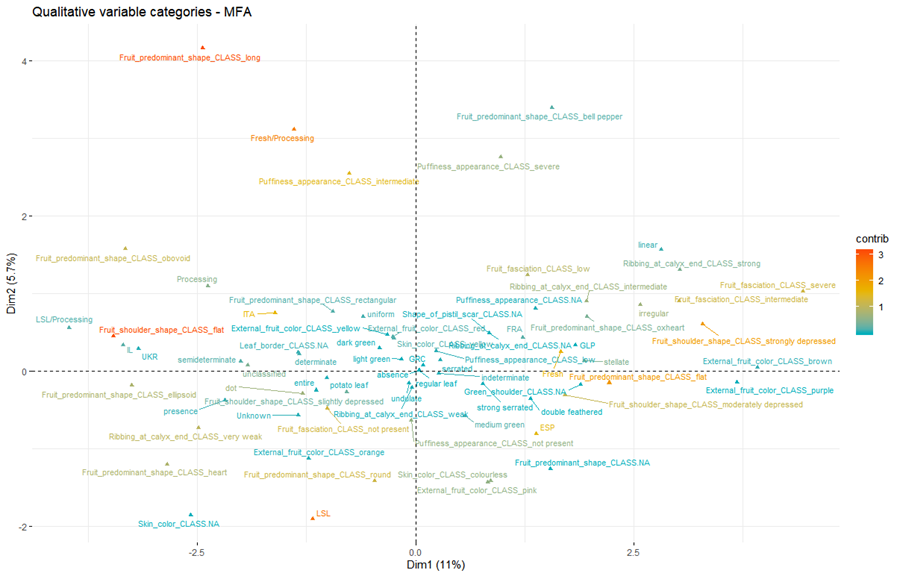
f**

**g**

**
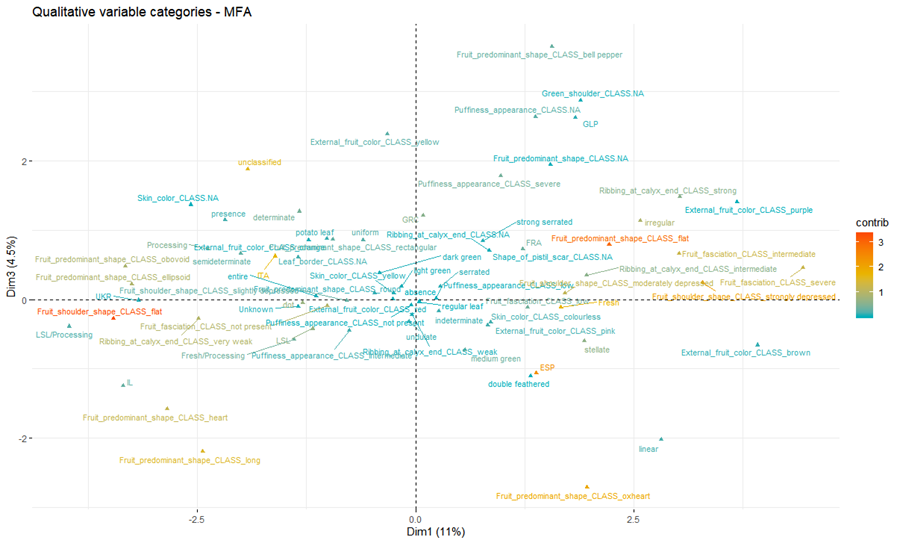
**

**
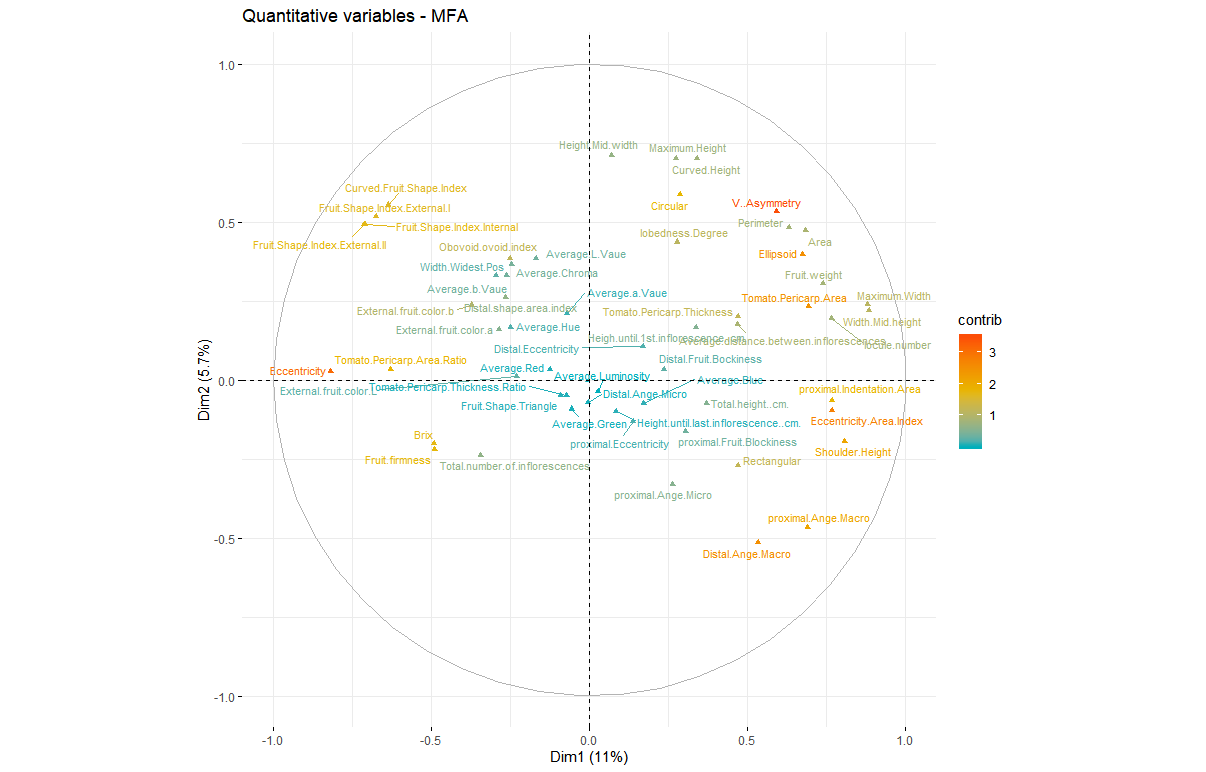
h**

**
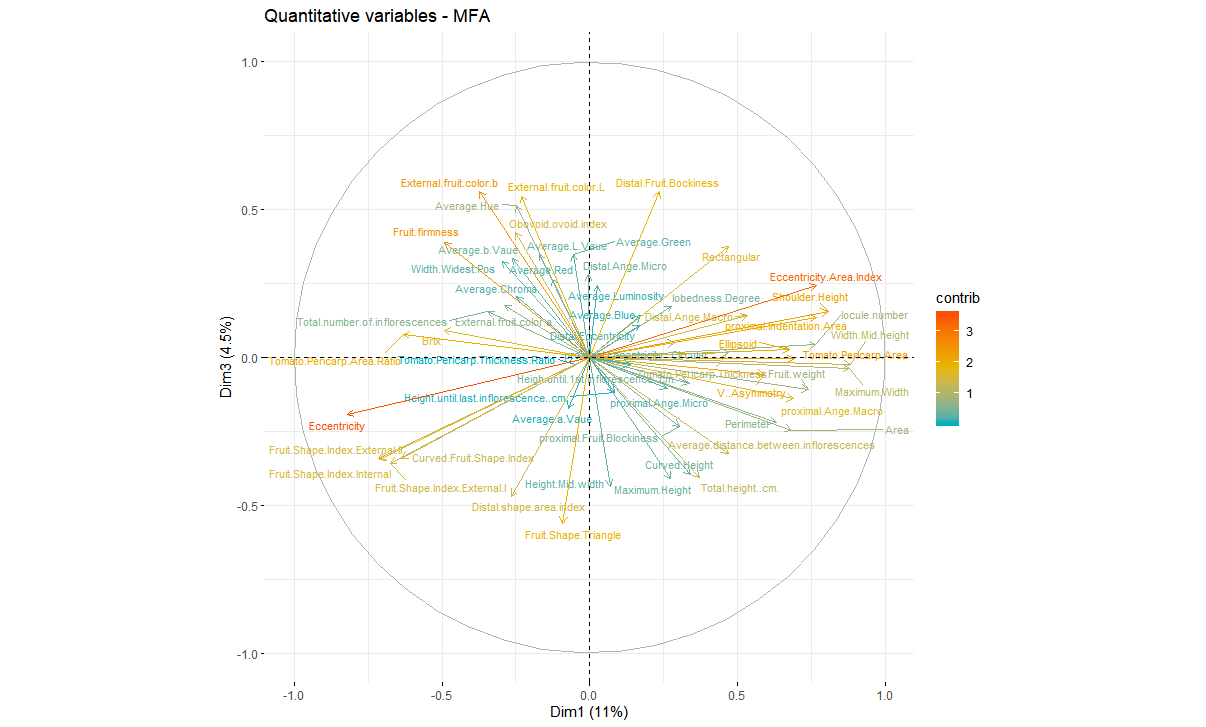
i**

**Fig. S12. MFA results.** a to c) Bar plot of the contributions of the groups of traits to the first dimensions of MFA a) to the dimension 1 (dim 1). b) to the dimension 2 (dim 2) and c) to the dimension 3 (dim 3). d to i) Variable factor maps illustrating the contribution and the relationship of each group of variables (a, b) or individual qualitative (f, g) and quantitative variables (h, i) to dimensions 1 and 2 (d, f, h) and dimensions 1 and 3 (e, g, i). The red dashed line on the graph above indicates the expected average uniform contribution (cut-off).


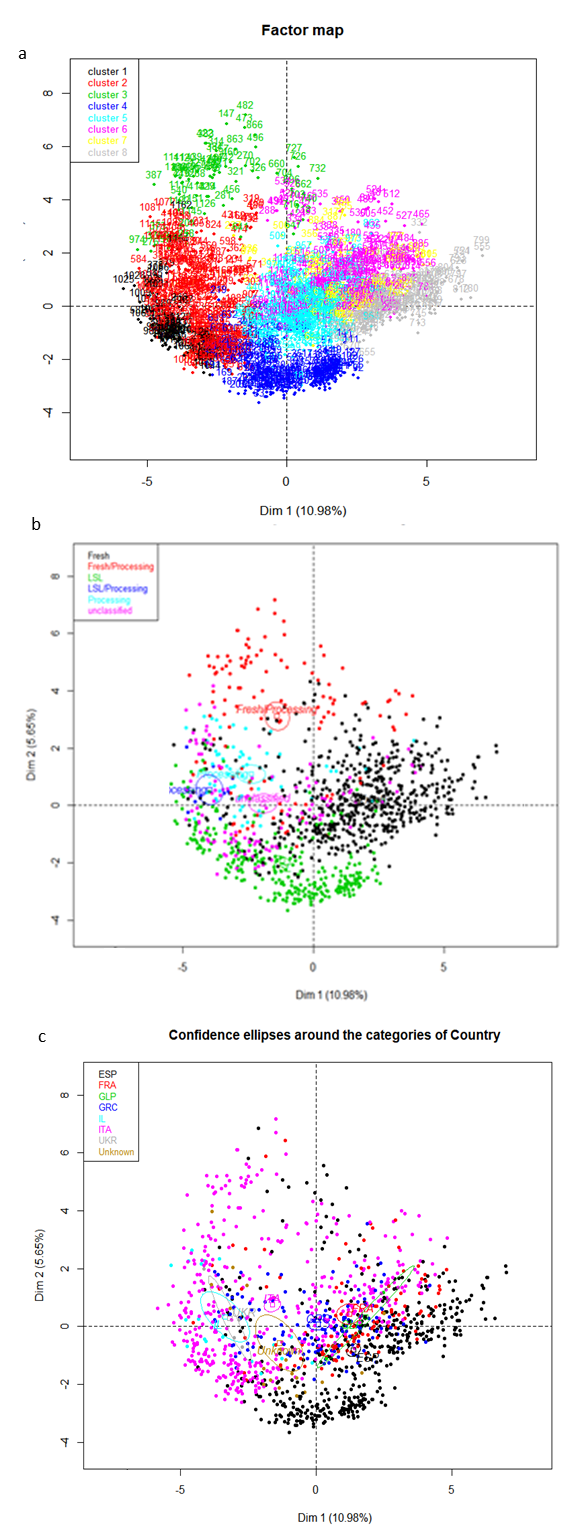


**Fig. S13. MFA individual factor maps showing the position of 1,999 tomato samples in the MFA space and the ability to discriminate clusters** (a) by b) traditional end use and c) country of origin


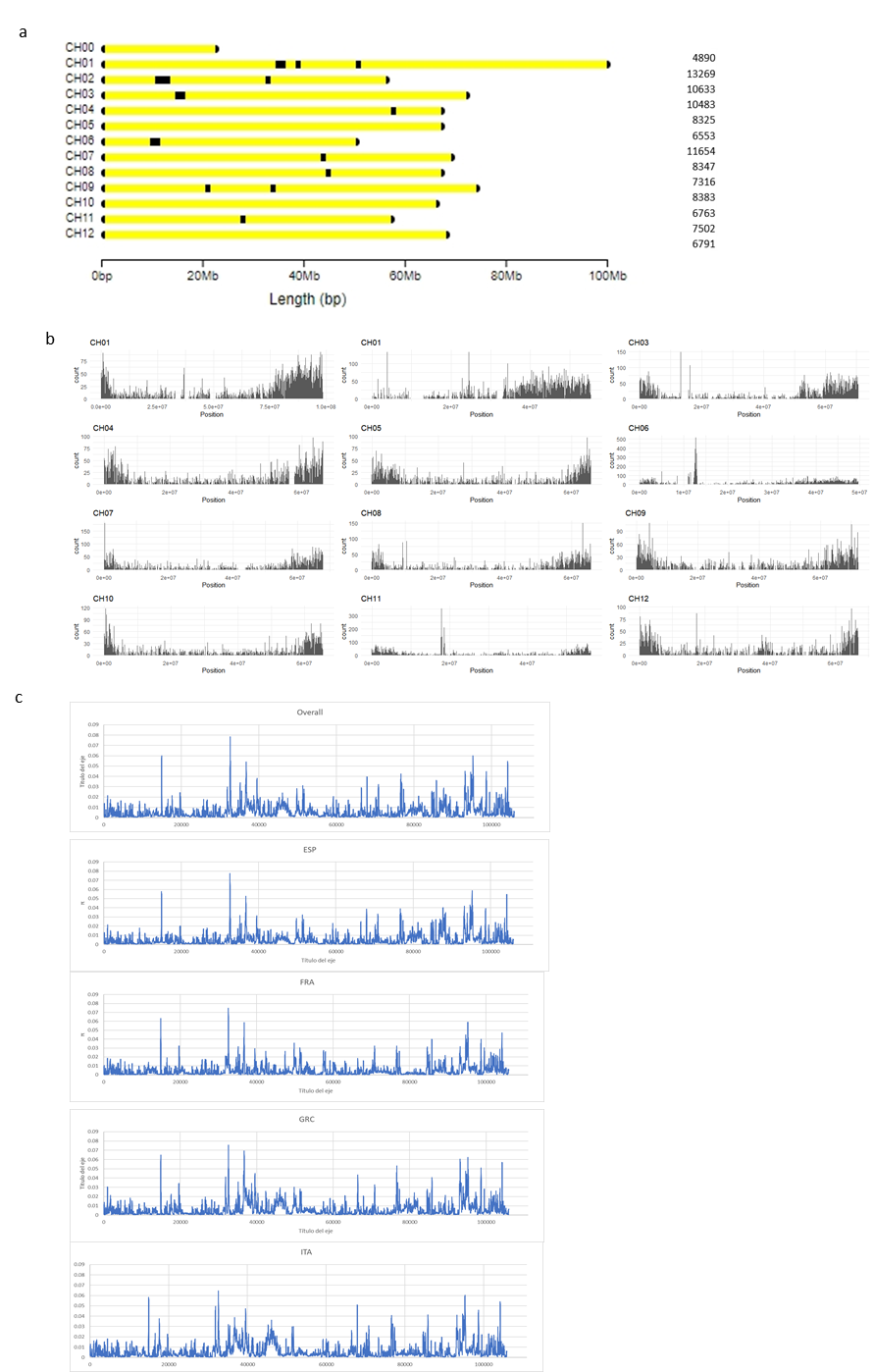


**Fig. S14. Distribution of the 110909 SNPs on chromosomes and nucleotide diversity index (π)**. a) distribution across the 12 chromosomes; b) SNPS density, bars represent the number of SNPs per 100Kb, c) Nucleotide diversity (π) per SNP across genome for the overall collection and the country subsets


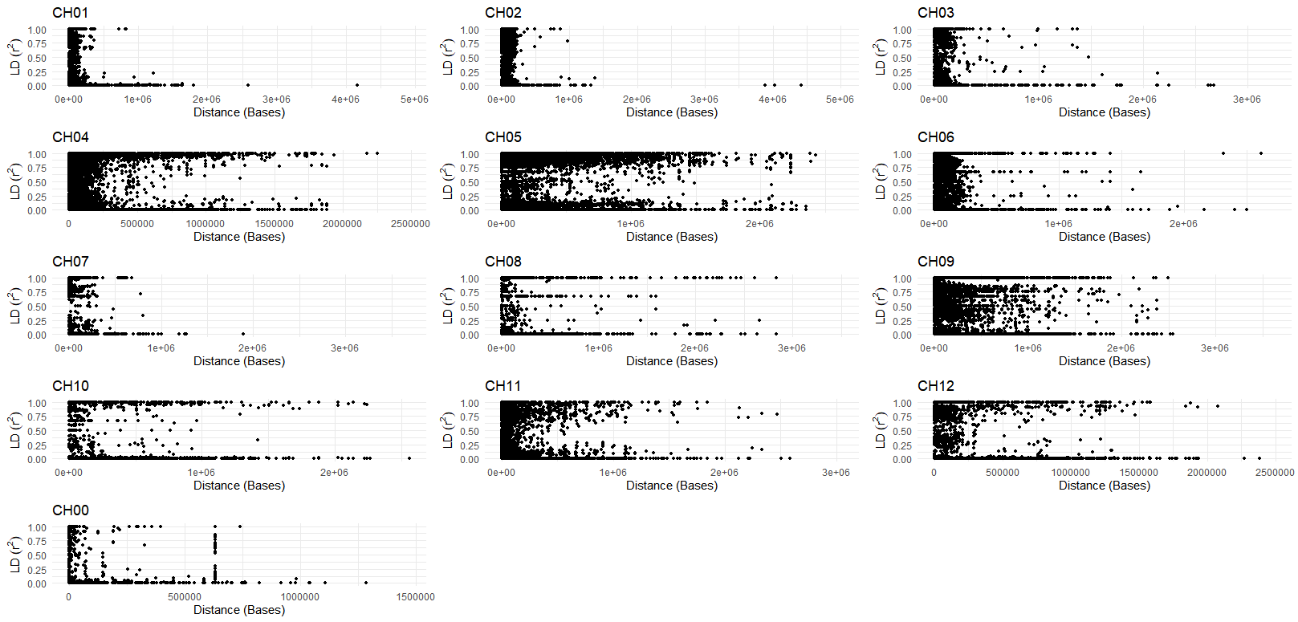

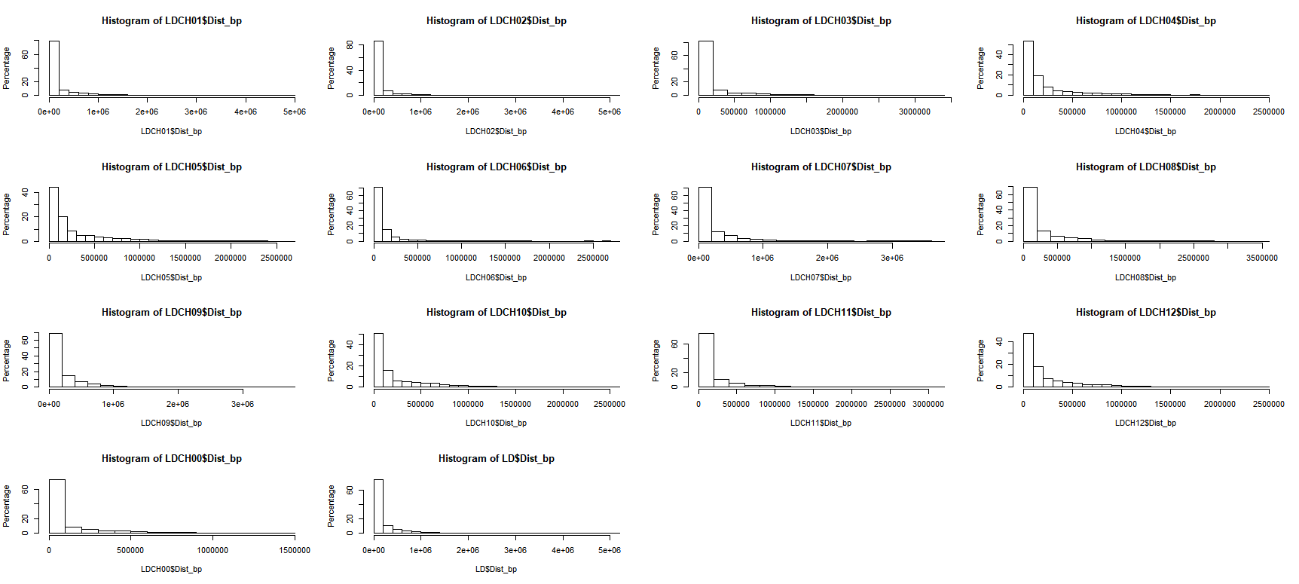


a

b

**Fig. S15. Linkage disequilibrium (LD) in Traditional European tomato.** a) Histogram for LD, R2 is represented, and b) LD in response to distance between SNPs.


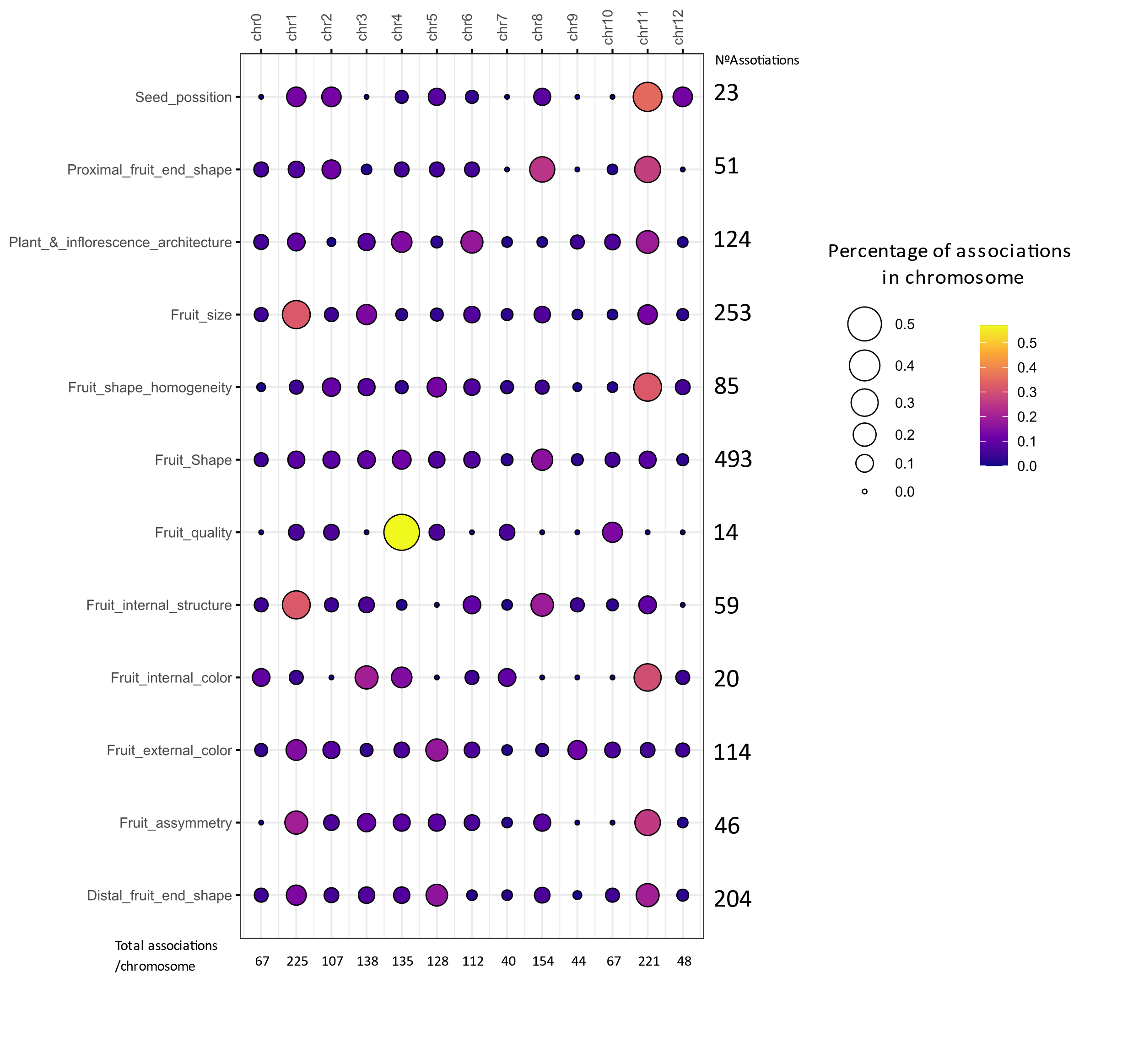
**Fig. S16. Distribution of trait associations per chromosome.** The size of the circle and colour scale indicate the percentage of associations in each chromosome per trait category.


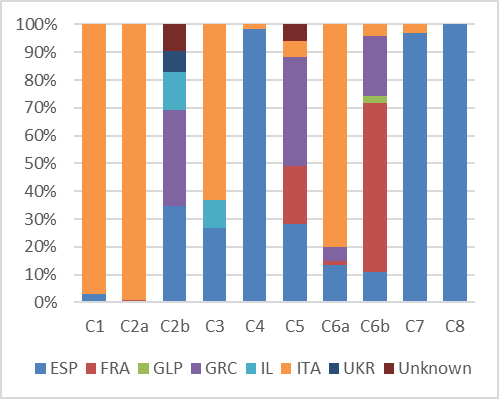

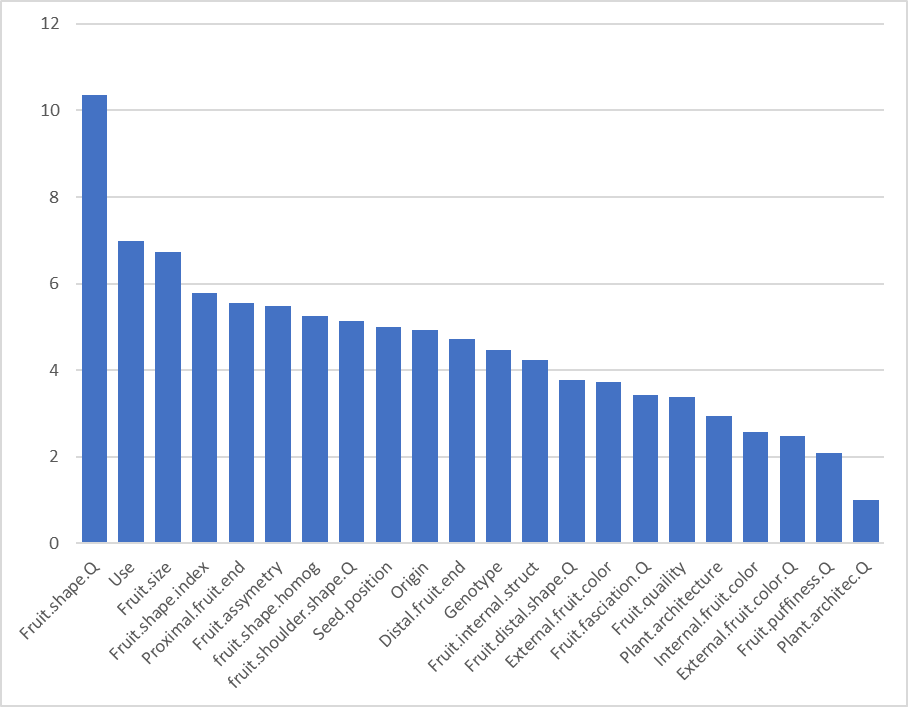


a

b

**Fig. S17. Integration analysis.** a) Contribution of each variable group to separate traditional tomato samples in MFA analysis using phenotype, genotype, use and country of origin. The red line represented the contribution cut-off. b) Origin of the accessions in the different clusters generated by MFA-HCPC when genotyping data is included.

Supplementary tables

**Table S1. Phenotyped traits and abbreviations.** The groups of traits and the quantitative and qualitative nature of the traits is indicated.

| **Trait** | **Abbreviation** | **Trait** | **Abbreviation** |
| --- | --- | --- | --- |
| ***FRUIT COLOUR*** |  | **Fruit shape** |  |
| **Fruit external colour** |  | **Qualitative** |  |
| **Qualitative** |  | Fruit predominant shape (nominal) | *fps* |
| External fruit colour (nominal) | *fec* | **Quantitative** |  |
| Green shoulder (ordinal) | *GSh* | Curved Fruit Shape Index | *fse.curved* |
| Skin colour (ordinal) | *SkC* | Fruit Shape Index External I | *fse.I* |
| **Quantitative** |  | Fruit Shape Index External II | *fse.II* |
| External fruit colour LAB a | *fec.a** | Fruit Shape Index Internal | *fsi* |
| External fruit colour LAB b | *fec.b** | **Fruit shape homogeneity** |  |
| External fruit colour LAB L | *fec.L** | **Qualitative** |  |
| **Fruit internal colour** |  | Fruit fasciation (ordinal) | *fas* |
| **Quantitative** |  | **Quantitative** |  |
| Internal fruit colour LAB Average a Value | *fic.a* | Circular | *cir* |
| Internal fruit colour LAB Average b Value | *fic.b* | Ellipsoid | *ell* |
| Internal fruit colour LAB Average Chroma | *fic.C* | lobedness Degree | *lob* |
| Internal fruit colour LAB Average Hue | *fic.H* | Rectangular | *rec* |
| Internal fruit colour LAB Average L Value | *fic.L* | **Fruit size** |  |
| Internal fruit colour RGB Average Blue | *fic.blue* | **Quantitative** |  |
| Internal fruit colour RGB Average Green | *fic.green* | Area | *ar* |
| Internal fruit colour RGB Average Luminosity | *fic.luminosity* | Curved Height | *H.curved* |
| Internal fruit colour RGB Average Red | *fic.red* | Fruit weight (g) | *fw* |
| ***FRUIT QUALITY*** |  | Height Mid-width | *H.mid* |
| **Quantitative** |  | locule number | *lcn* |
| Fruit firmness | *firm* | Maximum Height | *H.max* |
| SSC (ºBrix) | *SSC* | Maximum Width | *W.max* |
| ***FRUIT MORPHOLOGY & SIZE*** |  | Perimeter | *per* |
| **Distal fruit end shape** |  | Width Mid-height | *W.mid* |
| **Qualitative** |  | Width Widest Position | *Ww* |
| Ribbing at calyx end (ordinal) | *rce* | **Proximal fruit end shape** |  |
| Shape of pistil scar (nominal) | *sps* | **Qualitative** |  |
| **Quantitative** |  | Fruit shoulder shape (ordinal) | *FShS* |
| Distal Angle Macro | *dan.macro* | **Quantitative** |  |
| Distal Angle Micro | *dan.micro* | Proximal Angle Macro | *pan.macro* |
| Distal end shape index | *desi* | Proximal Angle Micro | *pan.micro* |
| Distal Fruit Blockiness | *dblk* | Proximal Fruit Blockiness | *pblk* |
| **Fruit asymmetry** |  | Proximal Indentation Area | *piar* |
| **Quantitative** |  | Shoulder Height | *psh* |
| Fruit Shape Triangle | *tri* | **PLANT & INFLORESCENCE ARCHITECTURE** |  |
| Obovoid-ovoid symmetry index | *osi* | **Qualitative** |  |
| V, Asymmetry | *ver* | Growth habit (ordinal) | *GH* |
| **Fruit internal structure** |  | Jointless pedicel (binary) | *jp* |
| **Qualitative** |  | leaf border (ordinal) | *LeB* |
| Puffiness appearance (ordinal) | *puf* | Leaf shape (nominal) | *LeS* |
| **Quantitative** |  | **Quantitative** |  |
| Tomato Pericarp Area | *par* | Average distance between inflorescences | *ADI* |
| Tomato Pericarp Area Ratio | *par.R* | Height until 1st inflorescence (cm) | *Hu1I* |
| Tomato Pericarp Thickness | *ptk* | Height until last inflorescence (cm) | *HuLI* |
| Tomato Pericarp Thickness Ratio | *ptk.R* | Total plant height (cm) | *TH* |
| **Seed position** |  | Total number of inflorescences | *Tni* |
| **Quantitative** |  |  |  |
| Distal Eccentricity | *dec* |  |  |
| Eccentricity | *ecc* |  |  |
| Eccentricity Area Index | *eai* |  |  |
| Proximal Eccentricity | *pec* |  |  |

**Table S2. Phenotypic, usage and country of origin differentiation among traditional European tomato phenoclusters**. Numbers in red indicate values that are over-represented (expressed in %) or greater than the overall means. SD for cluster means is shown. Numbers in black indicate down-represented or lower than the overall means. ns: p > 0.05, not significantly describing the cluster. Values with asterisk *, **, ***, **** are statistically different at probability values of p ≤ 0.05, ≤ 0.01, ≤ 0.001 and ≤ 0.0001 respectively.

|  | **C1** | **C2** | **C3** | **C4** | **C5** | **C6** | **C7** | **C8** |
| --- | --- | --- | --- | --- | --- | --- | --- | --- |
| **Use** |  |  |  |  |  |  |  | **Use** |
| Fresh | 1.52%^****^ | 16.54%^****^ | 8.70%^****^ | 6.599%^****^ | **89.52%^****^** | **61.86%^****^** | **96.10%^****^** | **99.24%^****^** |
| Fresh/Processing | 1.5152%^**^ | ns | **85.51%^****^** | 0%^****^ | ns | ns | 0%^***^ | 0%^****^ |
| LSL | **78.79%^****^** | ns | 0%^****^ | **92.39%^****^** | 1.90%^****^ | 7.2165%^****^ | 1.30%^****^ | 0.76%^****^ |
| LSL/Processing | ns | **3.937%^****^** | ns | ns | ns | ns | ns | ns |
| Processing | 0%^*^ | **18.90%^****^** | ns | 1.02%^***^ | 0.48%^****^ | ns | ns | 0%^***^ |
| Country of origin |  |  |  |  |  |  |  |  |
| ESP | 3.03%^****^ | 8.66%^****^ | 27.54%^*^ | **93.91%^****^** | 17.14%^****^ | 10.31%^****^ | **75.32%^****^** | **97.73%^****^** |
| FRA | 0%^**^ | 3.94%^***^ | 2.90%^*^ | 0%^****^ | **26.19%^****^** | **15.46%^**^** | ns | 0%^****^ |
| GRC | 0%^***^ | ns | 0%^***^ | 0%^****^ | **44.76%^****^** | 3.09%^****^ | 2.60%^**^ | 2.27%^****^ |
| ITA | **96.97%^****^** | **66.93%^****^** | **59.42%^****^** | 2.54%^****^ | 6.67%^****^ | **70.62%^****^** | 6.49%^****^ | 0%^****^ |
| IL | ns | ns | **8.70%^***^** | ns | ns | 0%^*^ | ns | ns |
| UKR | ns | **1.58%^**^** | ns | ns | ns | ns | ns | ns |
| GLP | ns | ns | ns | ns | **1.43%**** | ns | ns | ns |
| **Distal fruit end shape** |  |  |  |  |  |  |  |  |
| Distal Angle Macro | 96.23±11.99^****^ | 104.34±23.407^****^ | 63.40±17.54^****^ | **149.01±16.39^****^** | **130.61±26.78^***^** | **135.36±17.60^****^** | 115.31±24.54^*^ | **144.70±24.54^****^** |
| Distal Angle Micro | 106.37±29.54^****^ | **156.76±29.97^****^** | 125.21±35.12^***^ | ns | ns | ns | ns | ns |
| Distal Fruit Blockiness | 0.41±0.11^****^ | **0.64±0.06^****^** | ns | **0.64±0.04^**^** | **0.64±0.04^***^** | **0.65±0.06^****^** | 0.52±0.07^****^ | **0.65±0.04^****^** |
| Distal shape area index | **0.08±0.07^****^** | ns | **0.07±0.08^****^** | 0.0005±0.0146^****^ | 0.0043±0.025^**^ | -0.0059±0.0258^****^ | **0.039±0.078^****^** | -0.0031±0.0189^****^ |
| Ribbing at calyx end intermediate | 0%^****^ | 4.33%^****^ | ns | 6.09%^****^ | ns | **43.81%^****^** | ns | **42.42%^****^** |
| Ribbing at calyx end strong | 0%^*^ | 0.79%^****^ | 0%^*^ | 0%^****^ | ns | **23.71%^****^** | 0%^**^ | ns |
| Ribbing at calyx end very weak | **78.79%^****^** | **47.24%^****^** | ns | ns | ns | 1.55%^****^ | 7.79%^***^ | 2.27%^****^ |
| Ribbing at calyx end weak | 21.21%^****^ | ns | **68.12%^***^** | **74.62%^****^** | ns | 27.84%^****^ | **67.53%^***^** | ns |
| Shape of pistil scar dot | 100%^****^ | **93.31%^****^** | **98.55%^****^** | **91.37%^****^** | 42.8571%^****^ | 30.41%^****^ | 15.58%^****^ | 27.279%^****^ |
| Shape of pistil scar irregular | 0%^****^ | 0.79%^****^ | 0%^****^ | 0%^****^ | **23.33%^****^** | **44.33%^****^** | ns | **21.22%^*^** |
| Shape of pistil scar linear | ns | 0%^*^ | ns | ns | ns | ns | **11.69%^****^** | ns |
| Shape of pistil scar stellate | 0%^****^ | 2.36%^****^ | 1.45%^****^ | 8.13%^****^ | **31.43%^****^** | ns | **63.64%^****^** | **46.21%^****^** |
| **External fruit colour** |  |  |  |  |  |  |  |  |
| External fruit colour a | **26.40±4.40^****^** | **23.58±6.95^****^** | ns | 20.05±3.04^****^ | 21.46±5.28^*^ | **24.30±4.19^****^** | 19.77±3.597^****^ | 19.43±3.01^****^ |
| External fruit colour b | **24.63±6.07^****^** | **25.57±7.06^****^** | ns | 14.44±2.54^****^ | **23.84±5.51^****^** | **23.42±3.95^****^** | 17.98±5.92^****^ | 13.87±2.88^****^ |
| External fruit colour L | ns | **41.01±4.63^****^** | 37.38±3.45^****^ | ns | **41.44±4.35^****^** | **40.14±2.54^**^** | 37.00±4.16^****^ | 34.73±2.52^****^ |
| External fruit colour brown | ns | ns | ns | ns | ns | ns | ns | **5.30%^****^** |
| External fruit colour orange | ns | **3.54%^**^** | ns | ns | ns | ns | ns | ns |
| External fruit colour pink | 4.55%^****^ | 5.91%^****^ | 0%^****^ | **77.67%^****^** | 10.95%^****^ | 9.28%^****^ | ns | ns |
| External fruit colour red | **92.42%^****^** | **84.25%^****^** | **100%^****^** | 21.32%^****^ | **83.81%^****^** | **82.99%^***^** | ns | ns |
| External fruit colour yellow | ns | **5.91%^**^** | ns | 0%^**^ | ns | **5.6701%^*^** | ns | 0%^*^ |
| Green shoulder dark green | **18.18%^*^** | ns | ns | 2.03%^****^ | **17.14%^****^** | ns | ns | 0%^****^ |
| Green shoulder light green | **69.70%^****^** | **46.85%^**^** | ns | 16.24%^****^ | 31.43%^**^ | ns | ns | **54.55%^***^** |
| Green shoulder medium green | 12.12%^****^ | 15.748%^****^ | ns | **76.65%^****^** | 27.14%^**^ | 18.0412%^****^ | **57.14%^****^** | ns |
| Green shoulder uniform | 0%^****^ | **25.98%^****^** | ns | 5.08%^****^ | **23.81%^**^** | **26.29%^****^** | 3.90%^***^ | 3.79%^****^ |
| Skin colour: colourless | 4.55%^****^ | 6.2992%^****^ | 0%^****^ | **78.68%^****^** | 11.4286%^****^ | 10.82%^****^ | ns | ns |
| Skin colour: yellow | **95.45%^****^** | **93.31%^****^** | **100%^****^** | 21.32%^****^ | **88.5714%^****^** | **89.18%^****^** | ns | ns |
| **Fruit internal colour** |  |  |  |  |  |  |  |  |
| Average a Value | ns | ns | **26.31±6.46^****^** | ns | 18.21±7.77^****^ | **24.87±6.92^****^** | ns | 20.90±2.94^*^ |
| Average b Value | **27.27±9.10^***^** | **26.50±7.33^****^** | **25.84±14.09^*^** | 14.32±2.80^****^ | **25.58±9.44^****^** | **28.90±6.32^****^** | ns | 12.85±7.69^****^ |
| Average Blue | 57.68±8.19^**^ | 60.06±14.84^****^ | 59.50±13.93^*^ | ns | **73.57±15.02^****^** | 57.6729±16.6333^****^ | ns | ns |
| Average Chroma | **35.63±8.36^**^** | **35.30±7.20^****^** | **37.73±13.51^****^** | 26.81±3.18^****^ | ns | **38.61±7.1734^****^** | ns | 25.037±6.72^****^ |
| Average Green | ns | ns | 76.74±14.09^**^ | ns | **90.72±17.30^****^** | 79.9494±19.1356^**^ | 78.18±13.37^**^ | 76.20±7.73^****^ |
| Average Hue | **50.19±10.57^**^** | **51.214±14.9504^****^** | ns | 32.77±4.88^****^ | **53.90±14.19^****^** | **49.66±9.47^****^** | 35.77±13.77^****^ | 30.03±8.55^****^ |
| Average L Value | ns | **44.69±8.23^****^** | **46.69±13.79^***^** | 37.09±4.54^****^ | **44.98±8.27^****^** | **48.4448±9.5485^****^** | 39.04±14.59^**^ | 33.21±6.85^****^ |
| Average Luminosity | ns | ns | ns | **97.76±8.94^*^** | **102.26±11.21^****^** | 93.001±13.04^****^ | ns | 92.29±5.21^****^ |
| Average Red | ns | ns | ns | **144.16±10.65^**^** | **143.88±10.66^**^** | ns | 138.40±13.93^*^ | 133.76±6.78^****^ |
| **Fruit quality** |  |  |  |  |  |  |  |  |
| Brix | **6.50±1.18^****^** | **5.631.142^****^** | **5.54±1.34^*^** | **6.04±0.97^****^** | 4.72±1.058^****^ | ns | 4.17±0.88^****^ | 3.75±0.80^****^ |
| Fruit firmness | **53.63±8.87^****^** | **50.65±9.96^****^** | 40.61±10.66^**^ | **54.20±10.26^****^** | ns | ns | 32.13±10.60^****^ | 26.23±6.97^****^ |
| **Fruit shape** |  |  |  |  |  |  |  |  |
| Fruit predominant shape bell pepper | ns | ns | ns | ns | ns | **3.61%^****^** | ns | ns |
| Fruit predominant shape ellipsoid | 0%^*^ | **23.23%^****^** | 0%^**^ | ns | 1.43%^***^ | 0%^****^ | 1.30%^*^ | 0.76%^***^ |
| Fruit predominant shape flat | 0%^****^ | 0.79%^****^ | 0%^****^ | **43.15%^*^** | **47.62%^****^** | **78.87%^****^** | 0%^****^ | **62.89%^****^** |
| Fruit predominant shape heart | **80.30%^****^** | 1.57%^****^ | 0%^**^ | ns | 0.48%^****^ | 0%^****^ | 1.30%^*^ | ns |
| Fruit predominant shape long | ns | 3.54%^*^ | **92.75%^****^** | 0.51%^****^ | 0%^****^ | 0%^****^ | ns | 0%^****^ |
| Fruit predominant shape obovoid | 16.67%^**^ | 18.50%^****^ | ns | 0.51%^****^ | 0.48%^****^ | ns | 0%^**^ | 0%^***^ |
| Fruit predominant shape oxheart | 0%^**^ | 1.18%^****^ | 0%^**^ | 0.51%^****^ | 0.95%^****^ | 0.52%^****^ | **93.51%^****^** | 3.03%^*^ |
| Fruit predominant shape rectangular | 0%^**^ | **16.14%^****^** | ns | ns | ns | 3.61%^*^ | 0%^**^ | ns |
| Fruit predominant shape round | 0%^****^ | **35.04%^****^** | 0%^****^ | **35.53%^****^** | **41.90%^****^** | 9.28%^****^ | 1.30%^****^ | ns |
| Fruit Shape Index External I | **1.25±0.18^****^** | **1.20±0.27^****^** | **1.97±0.27^****^** | 0.88±0.16^****^ | 0.84±0.10^****^ | 0.74±0.12^****^ | ns | 0.75±0.08^****^ |
| Fruit Shape Index External II | **1.22±0.18^****^** | **1.18±0.29^****^** | **1.99±0.29^****^** | 0.80±0.18^****^ | 0.77±0.12^****^ | 0.63±0.15^****^ | ns | 0.63±0.10^****^ |
| Fruit Shape Index Internal | **1.22±0.18^****^** | **1.18±0.29^****^** | **1.99±0.29^****^** | 0.80±0.18^****^ | 0.77±0.12^****^ | 0.63±0.15^****^ | ns | 0.64±0.10^****^ |
| Curved Fruit Shape Index | **1.29±0.18^****^** | **1.26±0.28^****^** | **2.10±0.31^****^** | 0.92±0.15^****^ | 0.93±0.14^****^ | 0.80±0.12^****^ | ns | 0.81±0.06^****^ |
| **Fruit asymmetry** |  |  |  |  |  |  |  |  |
| Fruit Shape Triangle | **1.93±0.72^****^** | 1.10±0.15^****^ | **1.39±0.35^****^** | ns | 1.17±0.14^***^ | 1.15±0.18^****^ | **1.50±0.27^****^** | ns |
| Obovoid ovoid index | -0.20±0.07^****^ | -**0.03±0.14^****^** | **-0.08±0.18^*^** | -0.16±0.05^****^ | ns | **-0.09±0.10^*^** | -0.20±0.06^****^ | ns |
| V.Asymmetry | 0.05±0.02^****^ | 0.06±0.03^****^ | **0.18±0.12^****^** | 0.08±0.02^****^ | ns | **0.18±0.08^****^** | **0.20±0.08^****^** | **0.17±0.07^****^** |
| **Fruit internal structure** |  |  |  |  |  |  |  |  |
| Puffiness appearance intermediate | 0%^**^ | ns | **50.72%^****^** | 3.55%^**^ | 2.86%^****^ | ns | ns | ns |
| Puffiness appearance low | ns | ns | ns | **32.99%^**^** | ns | **30.93%^*^** | ns | ns |
| Puffiness appearance NA | ns | ns | ns | ns | **4.29%^****^** | ns | ns | ns |
| Puffiness appearance not present | **80.30%^**^** | ns | 18.84%^****^ | ns | **72.86%^***^** | 46.39^****^ | **74.03%^*^** | ns |
| Puffiness appearance severe | ns | ns | ns | 0%^***^ | 0%^***^ | **11.34%^****^** | ns | ns |
| Tomato Pericarp Area | 4.10±0.98^****^ | 5.63±3.36^****^ | 7.47±3.62^****^ | 8.56±2.36^****^ | **13.04±6.39^****^** | **16.43±7.04^****^** | **14.60±3.67^****^** | **15.86±4.05^****^** |
| Tomato Pericarp Area Ratio | **0.43±0.033^**^** | **0.45±0.041^****^** | **0.46±0.052^****^** | **0.44±0.03^****^** | ns | 0.37±0.07^****^ | 0.32±0.07^****^ | 0.30±0.08^****^ |
| Tomato Pericarp Thickness | 0.83±0.11^****^ | 0.96±0.32^****^ | 1.14±0.29^*^ | ns | **1.38±0.44^****^** | **1.52±0.47^****^** | ns | **1.32±0.32^*^** |
| Tomato Pericarp Thickness Ratio | ns | ns | ns | ns | ns | ns | ns | 0.20±0.01^**^ |
| **Fruit shape homogeneity** |  |  |  |  |  |  |  |  |
| Fruit fasciation intermediate | 0%^**^ | 0.39%^****^ | 1.45%^**^ | 0%^****^ | 2.38%^****^ | **31.96%^****^** | ns | **28.0303%^****^** |
| Fruit fasciation low | 0%^****^ | 5.12%^****^ | **30.45%^**^** | 2.5381%^****^ | 8.57%^***^ | **43.30%^****^** | **36.36%^****^** | ns |
| Fruit fasciation not present | **100%^****^** | **94.49%^****^** | ns | **97.46%^****^** | **88.57%^****^** | 17.53%^****^ | 50.654%^***^ | 21.21%^****^ |
| Fruit fasciation severe | 0%^*^ | 0%^****^ | 0%^*^ | 0%^****^ | 0.48%^***^ | ns | ns | **28.03%^****^** |
| Ellipsoid | 0.03±0.01^****^ | 0.04±0.01^****^ | **0.07±0.01^****^** | 0.04±0.01^****^ | 0.05±0.01^*^ | **0.062±0.02^****^** | **0.058±0.017^****^** | **0.06±0.02^****^** |
| Circular | 0.07±0.03^****^ | 0.08±0.05^****^ | **0.22±0.04^****^** | 0.08±0.04^****^ | 0.09±0.04^****^ | **0.14±0.05^****^** | 0.09±0.03^**^ | **0.13±0.04^****^** |
| Lobedness Degree | ns | 1.32±0.52^****^ | **2.08±0.82^****^** | 1.29±0.59^****^ | ns | 2.22±0.93^****^ | ns | **1.81±0.65^*^** |
| Rectangular | 0.48±0.03^****^ | 0.52±0.03^****^ | 0.52±0.04^****^ | **0.55±0.02^****^** | **0.54±0.02^****^** | **0.55±0.03^****^** | 0.49±0.04^****^ | **0.56±0.02^****^** |
| **Proximal fruit end shape** |  |  |  |  |  |  |  |  |
| Fruit shoulder shape flat | **77.27%^****^** | **56.30%^****^** | **46.38%^****^** | 10.66%^****^ | 1.43%^****^ | 0.52%^****^ | 0%^****^ | 0%^****^ |
| Fruit shoulder shape moderately depressed | 0%^****^ | 0.79%^****^ | 10.14%^****^ | ns | **38.10%^**^** | **48.10%^****^** | **44.16%^**^** | **50.76%^****^** |
| Fruit shoulder shapes lightly depressed | 22.73%^*^ | **42.919%^**^** | ns | **43.65%^**^** | **46.19%^****^** | 20.10%^****^ | ns | 12.8788%^****^ |
| Fruit shoulder shape strongly depressed | 0%^****^ | 0%^****^ | 2.90%^***^ | ns | ns | **30.41%^****^** | **25.97%^**^** | **36.3636%^****^** |
| Proximal Ange Macro | 100.75±27.15^****^ | 102.55±34.73^****^ | 67.85±40.48^****^ | **187.53±47.05^****^** | **159.53±36.76^**^** | 138.51±38.25^**^ | **177.68±57.42^****^** | **231.33±33.34^****^** |
| Proximal Ange Micro | ns | 184.46±50.62^****^ | 190.53±53.04 ^*^ | **239.49±37.09^****^** | 195.99±46.06^**^ | 195.06±44.72^**^ | ns | **230.28±34.44^****^** |
| Proximal Eccentricity | ns | 0.89±0.01^*^ | 0.89±0.01^**^ | **0.90±0.01^***^** | 0.89±0.06^**^ | ns | ns | ns |
| Proximal Fruit Blockiness | 0.70±0.11^****^ | 0.69±0.07^****^ | **0.79±0.09^****^** | **0.77±0.04^****^** | ns | ns | ns | **0.76±0.04^****^** |
| Proximal Indentation Area | 0.026±0.02^****^ | 0.03±0.03^****^ | 0.04±0.03^****^ | 0.08±0.03^*^ | ns | **0.16±0.08^****^** | **0.11±0.04^**^** | **0.16±0.05^****^** |
| Shoulder Height | 0.016±0.01^****^ | 0.02±0.01^****^ | 0.02±0.01^****^ | **0.06±0.02^****^** | ns | **0.08±0.03^****^** | ns | **0.082±0.02^****^** |
| **Seed position** |  |  |  |  |  |  |  |  |
| Distal Eccentricity | 0.88±0.02^****^ | ns | ns | ns | ns | ns | 0.88±0.01^*^ | **0.89±0.01^****^** |
| Eccentricity | **0.76±0.03^****^** | **0.77±0.02^****^** | **0.77±0.03^****^** | ns | ns | 0.67±0.05^****^ | ns | 0.67±0.05^****^ |
| Eccentricity Area Index | 0.39±0.05^****^ | 0.41±0.02^****^ | 0.42±0.03^****^ | **0.45±0.03^***^** | ns | **0.48±0.04^****^** | 0.43±0.03^*^ | **0.48±0.03^****^** |
| **Fruit size** |  |  |  |  |  |  |  |  |
| Area | 11.88±4.04^****^ | 15.77±8.00^****^ | **35.43±16.68^****^** | 17.70±4.27^****^ | ns | **36.23±13.90^****^** | **49.01±11.75^****^** | **44.94±10.93^****^** |
| Curved Height | 4.46±0.88^****^ | 4.85±1.40^****^ | **9.12±2.42^****^** | 4.48±0.60^****^ | ns | **6.05±1.43^*^** | **8.30±1.28^****^** | **6.84±1.07^****^** |
| Fruit weight | 27.07±11.65^****^ | 48.00±34.71^****^ | 105.00±71.12^*^ | 58.94±20.59^****^ | **175.56±81.72^****^** | **191.30±119.49^****^** | **254.55±89.04^****^** | **252.56±93.83^****^** |
| Height Mid width | 4.22±0.86^****^ | 4.58±1.39^****^ | 8.61±2.13^****^ | 3.85±0.66^****^ | ns | 4.78±1.38^*^ | **7.100±1.20^****^** | **5.29±0.87^*^** |
| locule number | 2.23±0.45^****^ | 2.57±0.89^****^ | 2.59±0.779^****^ | 3.45±1.05^****^ | **5.99±3.26^**^** | **8.66±3.11^****^** | **9.20±2.46^****^** | **8.65±3.62^****^** |
| Maximum Height | 4.37±0.88^****^ | 4.74±1.41^****^ | **9.01±2.40^****^** | 4.30±0.62^****^ | ns | ns | **7.99±1.21^****^** | **6.41±0.94^****^** |
| Maximum Width | 3.52±0.46^****^ | 3.98±0.94^****^ | 4.71±1.38^****^ | 5.01±0.80^****^ | **6.43±1.22^***^** | **7.74±1.56^****^** | **7.91±1.28^****^** | **8.62±1.23^****^** |
| Perimeter | 13.34±2.17^****^ | 15.33±4.56^****^ | **24.76±6.74^****^** | 16.47±2.19^****^ | ns | **24.35±5.30^****^** | **28.37±4.09^****^** | **27.37±3.99^****^** |
| Width Mid height | 3.46±0.44^****^ | 3.93±0.93^****^ | 4.49±1.29^****^ | 4.96±0.80^****^ | **6.37±1.20^***^** | **7.67±1.54^****^** | **7.75±1.28^****^** | **8.56±1.21^****^** |
| Width Widest Pos | 0.44±0.04^***^ | **0.50±0.06^****^** | **0.49±0.11^****^** | 0.44±0.02^****^ | ns | ns | 0.42±0.03^****^ | ns |
| **Plant and inflorescence architecture** |  |  |  |  |  |  |  |  |
| Growth habit determinate | ns | **16.54%^****^** | ns | 3.046%^**^ | ns | ns | 2.60%^*^ | 1.515%^***^ |
| Growth habit indeterminate | ns | 69.69%^****^ | ns | ns | ns | ns | **97.40%^***^** | **96.97%^****^** |
| Growth habit semideterminate | ns | **13.78%^****^** | ns | ns | ns | 2.062%^**^ | 0%^**^ | 1.52%^*^ |
| Jointless pedicel absence | ns | 95.28%^**^ | ns | ns | ns | ns | ns | ns |
| Jointless pedicel presence | ns | **4.72%^**^** | ns | ns | ns | ns | ns | ns |
| Leaf border entire | ns | ns | ns | **5.08%^*^** | ns | ns | ns | 0%^*^ |
| Leaf border NA | ns | **11.81%^****^** | ns | 2.5381%^*^ | ns | ns | ns | 1.52%^*^ |
| Leaf border serrated | 9.09±^****^ | ns | **60.87±^**^** | ns | **54.29%^**^** | ns | 31.17%^*^ | ns |
| Leaf border undulate | **75.76±^****^** | ns | ns | ns | 36.67%^*^ | ns | **63.64%^***^** | ns |
| Leaf shape potato leaf | ns | ns | ns | ns | ns | ns | ns | 0.76%^*^ |
| Leaf shape regular leaf | ns | ns | ns | ns | ns | ns | ns | **99.24%^*^** |
| Total height (cm) | 167.62±38.94^*^ | 155.67±45.94^****^ | ns | ns | **189.83±31.40^****^** | 158.22±35.55^****^ | **215.46±27.94^****^** | **214.96±36.15^****^** |
| Average distance between inflorescences (cm) | 18.62±3.43^****^ | 17.86±5.29^****^ | ns | ns | ns | **22.95±7.53^*^** | **26.89±5.09^****^** | **27.19±5.91^****^** |
| Height until 1st inflorescence (cm) | 14.63±8.17^****^ | 16.78±9.13^****^ | ns | ns | **27.64±7.56^****^** | ns | ns | ns |
| Height until last inflorescence (cm) | ns | 142.51±51.50^****^ | ns | ns | **177.68±34.13^****^** | 143.25±39.08^****^ | ns | ns |
| Total number of inflorescences | **8.88±2.09^****^** | **8.25±2.53^****^** | ns | ns | **8.27±1.87^****^** | 6.67±1.92^****^ | 6.36±1.41^****^ | 5.82±1.19^****^ |

**Table S3. Proportion of true vintage and traditionalized tomatoes in each phenocluster according to Blanca 2021** [1].

|  | **Phenocluster** | | | | | | | |  |
| --- | --- | --- | --- | --- | --- | --- | --- | --- | --- |
| **Rank 1** | **C1** | **C2** | **C3** | **C4** | **C5** | **C6** | **C7** | **C8** | **Total**  **TRADITOM** |
| **modern_fresh** | 0.00% | 3.14% | 1.89% | 3.19% | 28.42% | 8.59% | 1.39% | 12.98% | 10.20% |
| **modern_lsl_and_processing** | 2.13% | 11.52% | 3.77% | 15.96% | 2.63% | 1.84% | 2.78% | 0.76% | 5.42% |
| **modern_processing** | 0.00% | 11.52% | 7.55% | 0.00% | 0.53% | 0.00% | 0.00% | 0.00% | 2.87% |
| **SLC_Peru_MA** | 0.00% | 5.24% | 0.00% | 4.26% | 0.00% | 0.00% | 0.00% | 0.00% | 1.49% |
| **SPxSL** | 0.00% | 5.24% | 13.21% | 2.13% | 0.53% | 0.00% | 0.00% | 0.00% | 2.13% |
| **vintage** | 97.87% | 63.35% | 73.58% | 74.47% | 67.89% | 89.57% | 95.83% | 86.26% | 77.90% |
| **Total general** | 100.00% | 100.00% | 100.00% | 100.00% | 100.00% | 100.00% | 100.00% | 100.00% | 100.00% |

**Table S4. Linkage disequilibrium analysis and comparison with previous published data for landraces, breeding material and wild related species.** LD decay and block length per chromosome (ch) were above baseline r^2^ value > 0.135. SLL, *S.lycopersicum* ; Modern Admix, modern admixture; Old Admix, Old admixture; PIMP, *S.pimpinellifolium*. LD and distance are expressed in pb. For ch04, ch05, chr08-ch12 no decay was observed until the maximum block length.

|  | **TRADITOM** | | | | | | | | **Sacco et al. 2015** | **Bauchet et al. 2017** | | | | |
| --- | --- | --- | --- | --- | --- | --- | --- | --- | --- | --- | --- | --- | --- | --- |
| **CHR** | **Nº SNP** | **% SNPs** | **ch**  **length** | **LD decay** | **Max block length** | **Mean block size** | **SNPs in LD** | **%SNPs in LD** | **Landraces** | **SLL** | **Modern**  **Admix** | **Old**  **Admix** | **PIMP** | **Whole set** |
| **ch01** | 13269 | 0.120 | 9.85x10^7^ | 5.95x10^5^ | 1.22x10^6^ | 5.48x10^4^ | 666 | 0.050 | 6.85x10^5^ | 8.12x10^6^ | 4.24x10^6^ | 5.54x10^6^ | 1.80x10^5^ | 6.97x10^6^ |
| **ch02** | 10633 | 0.096 | 5.53x10^7^ | 3.69x10^5^ | 9.76x10^5^ | 6.38x10^4^ | 705 | 0.066 | 7.23x10^5^ | 4.01x10^6^ | 5.15x10^6^ | 7.39x10^6^ | 4.87x10^5^ | 7.13x10^6^ |
| **ch03** | 10483 | 0.095 | 7.08x10^7^ | 6.46x10^5^ | 2.14x10^6^ | 8.02x10^4^ | 562 | 0.054 | 1.04x10^6^ | 5.63x10^6^ | 1.75x10^6^ | 1.85x10^6^ | 1.49x10^6^ | 2.35x10^6^ |
| **ch04** | 8325 | 0.075 | 6.65x10^7^ | 2.25x10^6^ | 2.25x10^6^ | 3.76x10^5^ | 1014 | 0.122 | 1.79x10^6^ | 4.01x10^6^ | 4.61x10^6^ | 3.60x10^6^ | 2.42x10^5^ | 1.03x10^7^ |
| **ch05** | 6553 | 0.059 | 6.59x10^7^ | 2.43x10^6^ | 2.43x10^6^ | 3.71x10^5^ | 876 | 0.134 | 9.06x10^5^ | 6.55x10^6^ | 5.54x10^6^ | 4.72x10^7^ | 2.04x10^6^ | 9.01x10^6^ |
| **ch06** | 11654 | 0.105 | 4.98x10^7^ | 1.74x10^6^ | 2.62x10^6^ | 8.75x10^4^ | 1120 | 0.096 | 1.09x10^6^ | 1.56x10^7^ | 1.14x10^7^ | 5.87x10^6^ | 2.51x10^5^ | 6.03x10^6^ |
| **ch07** | 8347 | 0.075 | 6.80x10^7^ | 7.64x10^5^ | 8.00x10^5^ | 7.09x10^4^ | 305 | 0.037 | 5.46x10^5^ | 4.10x10^6^ | 2.97x10^6^ | 1.71x10^6^ | 4.72x10^5^ | 3.48x10^6^ |
| **ch08** | 7316 | 0.066 | 6.59x10^7^ | 2.83x10^6^ | 2.83x10^6^ | 3.00x10^5^ | 368 | 0.050 | 5.99x10^5^ | 8.69x10^6^ | 3.43x10^6^ | 2.06x10^6^ | 1.04x10^6^ | 3.56x10^6^ |
| **ch09** | 8383 | 0.076 | 7.25x10^7^ | 2.49x10^6^ | 2.49x10^6^ | 2.78x10^5^ | 1405 | 0.168 | 5.61x10^5^ | 4.50x10^6^ | 4.45x10^6^ | 1.75x10^6^ | 1.08x10^5^ | 5.56x10^6^ |
| **ch10** | 6763 | 0.061 | 6.55x10^7^ | 2.24x10^6^ | 2.24x10^6^ | 2.19x10^5^ | 333 | 0.049 | 5.81x10^5^ | 3.18x10^6^ | 2.97x10^7^ | 4.13x10^6^ | 7.30x10^4^ | 1.84x10^7^ |
| **ch11** | 7502 | 0.068 | 5.63x10^7^ | 2.47x10^6^ | 2.47x10^6^ | 1.57x10^5^ | 656 | 0.087 | 1.21x10^6^ | 4.15x10^6^ | 2.93x10^6^ | 6.37x10^6^ | 1.13x10^5^ | 5.55x10^6^ |
| **ch12** | 6791 | 0.061 | 6.71x10^7^ | 2.08x10^6^ | 2.08x10^6^ | 1.96x10^5^ | 480 | 0.071 | 1.00x10^6^ | 8.31x10^6^ | 5.52x10^6^ | 7.32x10^6^ | 6.31x10^6^ | 6.05x10^6^ |

**Table S5. Genetic diversity index and selection signatures per 1Kb window.** Nucleotide diversity (π), Watterson's estimator (θ), tajima D’.

| Collection | SNP Count | Average SNP Count | Segregating  Sites | π | θ | Tajima D' | Number accessions |
| --- | --- | --- | --- | --- | --- | --- | --- |
| TRADITOM | 110909 | 110884.8686 | 51401 | 0.000697 | 0.005973 | -2.57551 | 1316 |
| ESP | 110909 | 110890.8699 | 34968 | 0.000633 | 0.004498 | -2.59533 | 622 |
| FRA | 110909 | 110871.9038 | 10589 | 0.000584 | 0.001841 | -2.33037 | 101 |
| ITA | 110909 | 110882.6023 | 28257 | 0.000709 | 0.003929 | -2.54832 | 368 |
| GREECE | 110909 | 110877.0012 | 23750 | 0.000742 | 0.003631 | -2.56974 | 205 |

**Table S6. Summary of SNPs found significantly associated to a trait in each GWAS panel.** Nph, not phenotyped trait

|  | **TRIAL** | | | | | | | |  |
| --- | --- | --- | --- | --- | --- | --- | --- | --- | --- |
| **Trait** | **ACKTYMPAKY** | **ALCALAX** | **ARCA** | **GARBI** | **HUJI** | **INRA** | **PERELLO** | **UNITUS** | **Total SNPs** |
| Area |  | 1 | 1 |  | Nph | 3 | 3 |  | 8 |
| Average.a.Value | 1 |  |  |  | Nph | 3 |  |  | 4 |
| Average.b.Value |  |  |  |  | Nph | 1 |  |  | 1 |
| Average.Green | 3 |  |  |  | Nph |  |  |  | 3 |
| Average.Hue | 1 |  |  |  | Nph | 3 |  |  | 4 |
| Average.L.Value | 1 |  |  |  | Nph |  |  |  | 1 |
| Average.Luminosity | 1 |  |  |  | Nph |  |  |  | 1 |
| Brix |  |  |  |  | 2 | 1 |  |  | 3 |
| Circular |  | 1 |  |  | Nph |  |  | 1 | 2 |
| Curved.Fruit.Shape.Index |  | 2 | 1 | 3 | Nph | 5 | 3 | 5 | 19 |
| Curved.Height |  | 1 |  |  | Nph | 2 | 1 | 1 | 5 |
| Distal.Angle.Macro |  |  | 1 | 1 | Nph | 3 | 2 | 1 | 8 |
| Distal.Eccentricity |  | 1 | 1 |  | Nph |  |  | 2 | 4 |
| Distal.Fruit.Bockiness |  | 2 | 2 |  | Nph | 1 | 3 | 1 | 9 |
| Distal.shape.area.index |  | 2 | 1 |  | Nph |  | 4 | 1 | 8 |
| Eccentricity |  |  | 3 |  | Nph |  | 4 | 4 | 11 |
| Eccentricity.Area.Index |  | 1 | 2 |  | Nph |  | 2 | 1 | 6 |
| EFC_brown |  |  |  |  | 4 | 2 | 2 |  | 8 |
| EFC_orange |  | 1 | 1 |  |  |  | 4 | 8 | 14 |
| EFC_pink | 1 | 2 | 1 |  | 1 | 6 |  | 1 | 12 |
| EFC_purple |  |  |  |  |  | 3 |  |  | 3 |
| EFC_red | 1 | 5 | 1 |  | 2 | 1 | 1 |  | 11 |
| EFC_yellow | 8 |  |  |  | 2 | 5 | 3 |  | 18 |
| Ellipsoid | 1 |  |  |  | Nph | 1 | 2 | 2 | 6 |
| External.fruit.colour.a | 1 |  |  | Nph | Nph | 4 |  |  | 5 |
| External.fruit.colour.b |  | 1 |  | Nph | Nph |  |  |  | 1 |
| External.fruit.colour.L |  |  |  | Nph | Nph | 1 |  |  | 1 |
| FPS_bell_pepper |  |  |  |  | 12 | 7 |  | 9 | 28 |
| FPS_ellipsoid |  |  | 1 |  |  |  | 3 | 1 | 5 |
| FPS_flat |  |  | 1 |  | 3 | 2 |  | 3 | 9 |
| FPS_heart | 3 |  | 1 | 6 | 5 |  |  | 5 | 20 |
| FPS_long |  | 3 | 3 | 5 | 3 | 2 | 9 | 3 | 28 |
| FPS_ovoboid | 4 | 1 | 1 | 1 | 2 | 1 | 2 | 5 | 17 |
| FPS_oxheart |  | 3 | 4 |  | 6 | 3 | 1 | 7 | 24 |
| FPS_rectangular | 1 |  | 2 |  | 2 |  | 3 |  | 8 |
| FPS_round | 1 |  |  |  | 1 | 1 |  |  | 3 |
| Fruit.Shape.Index.External.I | 1 | 2 | 1 | 4 | Nph | 4 | 4 | 2 | 18 |
| Fruit.Shape.Index.External.II | 1 | 1 | 1 | 4 | Nph | 4 | 2 | 4 | 17 |
| Fruit.Shape.Index.Internal | 1 | 1 | 1 | 4 | Nph | 4 | 2 | 4 | 17 |
| Fruit.Shape.Triangle |  | 3 |  |  | Nph |  | 4 | 1 | 8 |
| Fruit.weight | 1 | 2 | 5 |  | 3 | 3 | 4 | 1 | 19 |
| Fruit_fasciation |  | 2 | 4 |  | 4 |  | 1 | 3 | 14 |
| Fruit_shoulder_shape | 1 |  | 3 |  | 4 | 4 | 2 |  | 14 |
| Green_shoulder |  |  |  |  |  |  | 1 |  | 1 |
| Growth_habit | 1 |  |  |  | 1 |  | 12 | 3 | 17 |
| Height.until.1st.inflorescence | 1 |  |  | Nph |  |  |  |  | 1 |
| Height.Mid.width |  |  |  |  | Nph | 2 | 1 | 1 | 4 |
| Jointless_pedicel | 11 | 3 |  | 1 | 5 | 17 | 33 |  | 70 |
| Leaf_shape |  |  |  |  | 1 |  |  |  | 1 |
| Lobedness.Degree |  |  | 1 |  | Nph |  |  |  | 1 |
| Locule.number | 2 | 3 | 4 |  | Nph | 4 | 6 | 3 | 22 |
| LSh_potato_leaf |  | 1 |  |  | 1 | 14 | 2 | 9 | 27 |
| LSh_regular_leaf |  | 1 |  |  |  | 1 | 2 | 9 | 13 |
| LShdouble_feathered |  |  |  | 2 | 1 |  |  |  | 3 |
| Maximum.Height |  | 1 |  |  | Nph | 2 | 1 | 1 | 5 |
| Maximum.Width |  |  | 3 |  | Nph | 3 | 6 |  | 12 |
| Obovoid.ovoid.index |  |  |  |  | Nph | 3 |  | 1 | 4 |
| Perimeter |  | 1 | 2 |  | Nph | 5 | 4 |  | 12 |
| Proximal.Angle.Macro |  |  | 1 |  | Nph |  | 2 | 1 | 4 |
| Proximal.Angle.Micro |  | 1 | 1 |  | Nph |  | 1 |  | 3 |
| Proximal.Eccentricity |  |  | 1 |  | Nph |  |  |  | 1 |
| Proximal.Fruit.Blockiness |  | 1 |  |  | Nph |  |  | 1 | 2 |
| Proximal.Indentation.Area |  |  | 3 |  | Nph |  | 1 |  | 4 |
| Puffiness_appearance |  |  |  |  | Nph | 4 |  |  | 4 |
| Rectangular |  | 4 |  |  | Nph | 2 | 1 | 1 | 8 |
| Ribbing_at_calyx_end | 1 |  |  |  | 4 | 3 |  | 1 | 9 |
| Shoulder.Height |  |  | 3 |  | Nph | 1 |  | 2 | 6 |
| Skin_colour | 1 | 2 | 1 |  | 2 | 4 |  | 1 | 11 |
| SPS_dot |  | 1 |  |  | 3 | 2 |  | 2 | 8 |
| SPS_irregular |  |  | 1 |  | 3 | 1 |  |  | 5 |
| SPS_linear |  |  | 35 | 1 | 1 | 1 |  | 4 | 42 |
| SPS_stellate |  | 1 | 1 |  | 1 |  |  | 1 | 4 |
| Tomato.Pericarp.Area |  |  |  |  | Nph |  |  | 1 | 1 |
| Tomato.Pericarp.Area.Ratio |  | 1 | 4 |  | Nph | 3 | 3 | 1 | 12 |
| Tomato.Pericarp.Thickness |  |  |  | 1 | Nph |  | 2 | 1 | 4 |
| Tomato.Pericarp.Thickness.Ratio |  | 4 | 2 |  | Nph |  |  |  | 6 |
| Total.number.of.inflorescences |  |  |  |  | Nph |  | 1 |  | 1 |
| V..Asymmetry |  | 2 | 4 |  | Nph |  | 2 |  | 8 |
| Width.Mid.height |  |  | 3 |  | Nph | 3 | 6 |  | 12 |
| Width.Widest.Pos | 1 |  |  |  | Nph |  | 1 | 1 | 3 |
| **Total SNPs** | **51** | **65** | **113** | **33** | **79** | **155** | **159** | **121** | **776** |

Legends for Datasets provided in separate files

**Dataset S1. Accessions and passport data for accessions included in the TRADITOM collection and analysed in this work.**

**Dataset S2. Field cultivation conditions for each trial.**

**Dataset S3. Curated phenotypic data.**

**Dataset S4. Basic statistic descriptors of the traits phenotyped.** Means denoted by a different letter indicate significant differences between country of origin (p < 0.001). Trait variation expressed as the index of qualitative variation (IQV) and the coefficient of variation (CV) for qualitative and quantitative variables, respectively. b) Heatmap comparing trait variation among countries.

**Dataset S5. Dataset used for MFA analysis containing less than 10% missing values.**

**Dataset S6. Complete set of imputed SNPs located on chromosomes and accessions.** Available at https://doi.org/10.5281/zenodo.5720772

**Dataset S7. Complete set of SNPs located on chromosomes and accessions that have passed all filters** (minimum read depth 3, less than 30% missing data per SNP, maximum heterozygosity per SNP 10% and less than 30% missing data per accession). Available at https://doi.org/10.5281/zenodo.5720772

**Dataset S8. SNPs found significantly associated to a trait by individual GWAS.**

**Dataset S9. GWAS meta-analysis associations and gene annotations.** Columns depict: the LD-block ID, the Trait category, the name of the trait, the lead SNP position (chromosome and physical position in bp, the maker ID, the start and end of candidate gene region, the start and end of lead SNP LD, the candidate gene ITAG ID, the gene description, the gene symbol, notes and references about candidate gene, other interesting genes in the region, distal gene(s) of interest, notes and references, Meta-analysis results (Effect allele (Allele1), Allele2, Frequency of allele 1 (Freq1), Frequency of allele 1 (FreqSE), Minimum and maximum allele frequency in the different GWAS panels (MinFreq, MaxFreq), Number of accessions tested (Weight), Z score, P-value, Direction effect allele 1 in different trials (+,-, ? unassayed), Heterogeneity estimate and statistics (HetISq, HetChiSq, HetDf, HetPVal), all genes in candidate region (in the case of no genes in candidate region, the nearest), previously QTL/association in SNP region, references and type (the same trait or related), Pleiotropy by LD-Block, and Pleiotropy by SNP, SL4.0 chromosome and SL4.0 position. Links for references are in a separated tab.

**Dataset S10. Comparison of associations found in meta-analysis with those obtained in individual GWAS panels.** Associations-SNPs are grouped according if they were identified in individual GWAS panels, meta-analysis or both. In the case of associations found only in individual GWAS panels the trial where the association was found is indicated.

**Dataset S11. HCPC results integrating genetic data with phenotypic, geographic and usage data.** Data are divided in 3 tabs: qualitative traits, quantitative traits and genotype. In qualitative trait tab is indicated the percentage of accessions in each cluster appertaining to the category, the p-value associated to the enrichment v-test and the cluster. In quantitative trait tab, is indicated the mean and the standard deviation of the trait in the cluster, the p-value associated to the enrichment v-test and the cluster. In genotype tab is indicated the cluster, the marker name, the genotype, the effect allele and alternative allele found in GWAS meta-analysis, the percentage of accessions in the cluster with the genotype, the p-value associated to the enrichment v-test and if the SNP is selected for a cluster signature. The sign of the v.test indicates whether trait or average in the cluster is enriched in that variable (in the case of qualitative variables) or greater than the overall means (in the case of quantitative variables). Positive values, over-represented; Negative values, down-represented. Traits or SNPs with p-value ≤ 0.0001, were considered significative.

**Dataset S12. Genomic inflation factor for each trait in each trial.**

**Dataset S13. All previously published QTL, GWAS analyses and genes cloned with known natural variability inside or overlapping with the candidate gene region of each lead SNP ± 1Mb.**

# SI References

1. Blanca J, Pons C, Montero-Pau J *et al.* European Vintage tomatoes galore: a result of farmers combinatorial assorting/swapping of a few diversity rich loci. *bioRxiv* 2021:2021.10.26.465840.

2. Sacco A, Ruggieri V, Parisi M *et al.* Exploring a tomato landraces collection for fruit-related traits by the aid of a high-throughput genomic platform. *PLoS One* 2015;**10**:1–20.

3. Bauchet G, Grenier S, Samson N *et al.* Use of modern tomato breeding germplasm for deciphering the genetic control of agronomical traits by Genome Wide Association study. *Theor Appl Genet* 2017;**130**:875–89.

4. Lin T, Zhu G, Zhang J *et al.* Genomic analyses provide insights into the history of tomato breeding. *Nat Genet* 2014;**46**:1220–6.

5. Razifard H, Ramos A, Della Valle AL *et al.* Genomic Evidence for Complex Domestication History of the Cultivated Tomato in Latin America. *Mol Biol Evol* 2020;**37**:1118–32.

6. Watterson GA. On the number of segregating sites in genetical models without recombination. *Theor Popul Biol* 1975;**7**:256–76.

7. Tajima F. Statistical method for testing the neutral mutation hypothesis by DNA polymorphism. *Genetics* 1989;**123**:585–95.

8. Simonsen KL, Churchill GA, Aquadro CF. Properties of statistical tests of neutrality for DNA polymorphism data. *Genetics* 1995;**141**:413–29.

9. Alercia A, International B, Nations F and AO of the U *et al.* FAO/Bioversity Multi-Crop Passport Descriptors V.2.1 [MCPD V.2.1] - December 2015. 2015.

10. Rodríguez GR, Muños S, Anderson C *et al.* Distribution of SUN, OVATE, LC, and FAS in the tomato germplasm and the relationship to fruit shape diversity. *Plant Physiol* 2011;**156**:275–85.

11. Rodríguez GR, Moyseenko JB, Robbins MD *et al.* Tomato Analyzer: a useful software application to collect accurate and detailed morphological and colorimetric data from two-dimensional objects. *J Vis Exp* 2010:1856.
