# Supplementary figures and images for: Atlas of phenotypic, genotypic and geographical diversity present in the European traditional tomato
Source: Hortic Res. 2022 May 17;9:uhac112. doi: 10.1093/hr/uhac112 (PMC9252105; doi:10.1093/hr/uhac112)

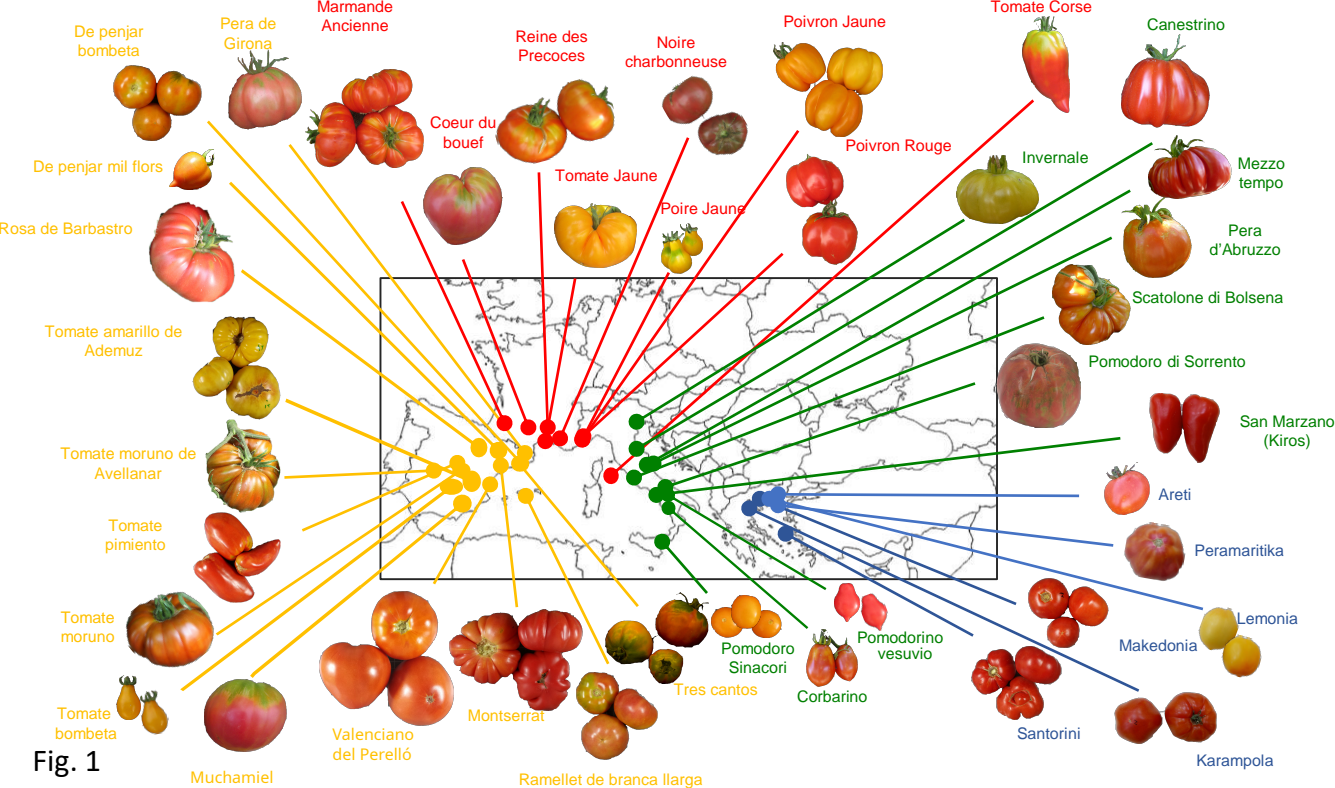

Fig. 1

a

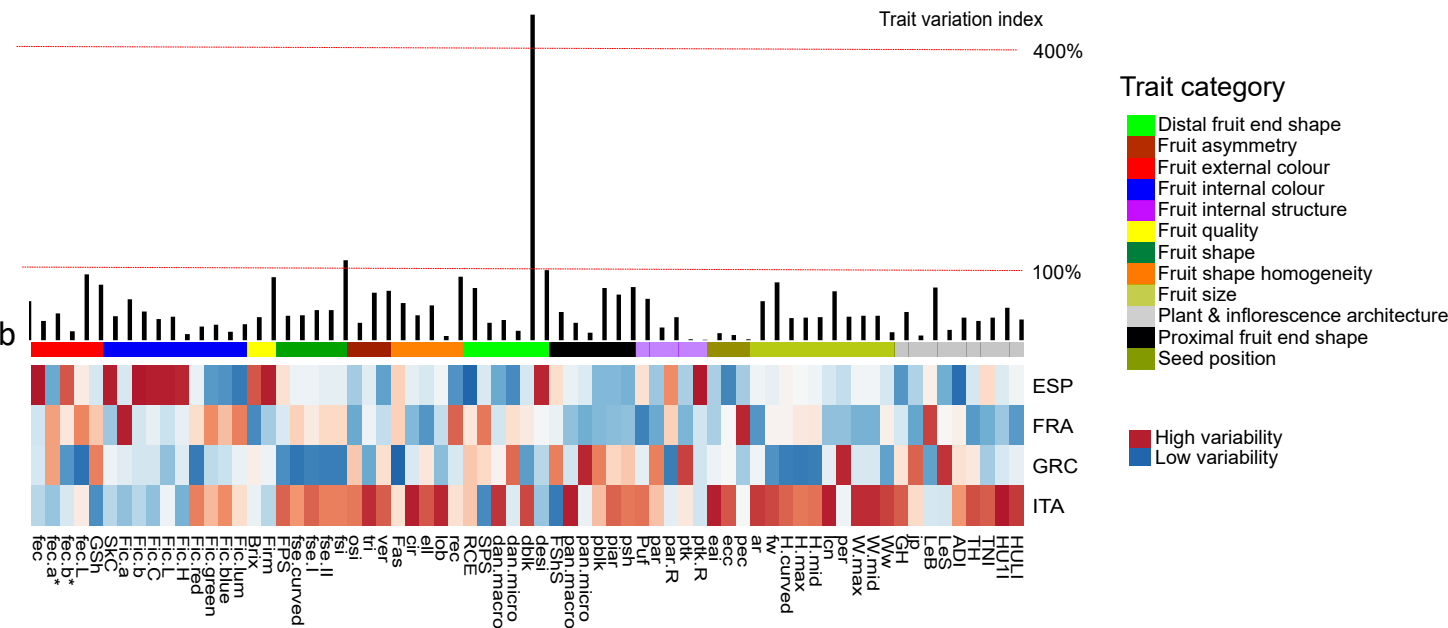

Fig. 2a and 2b

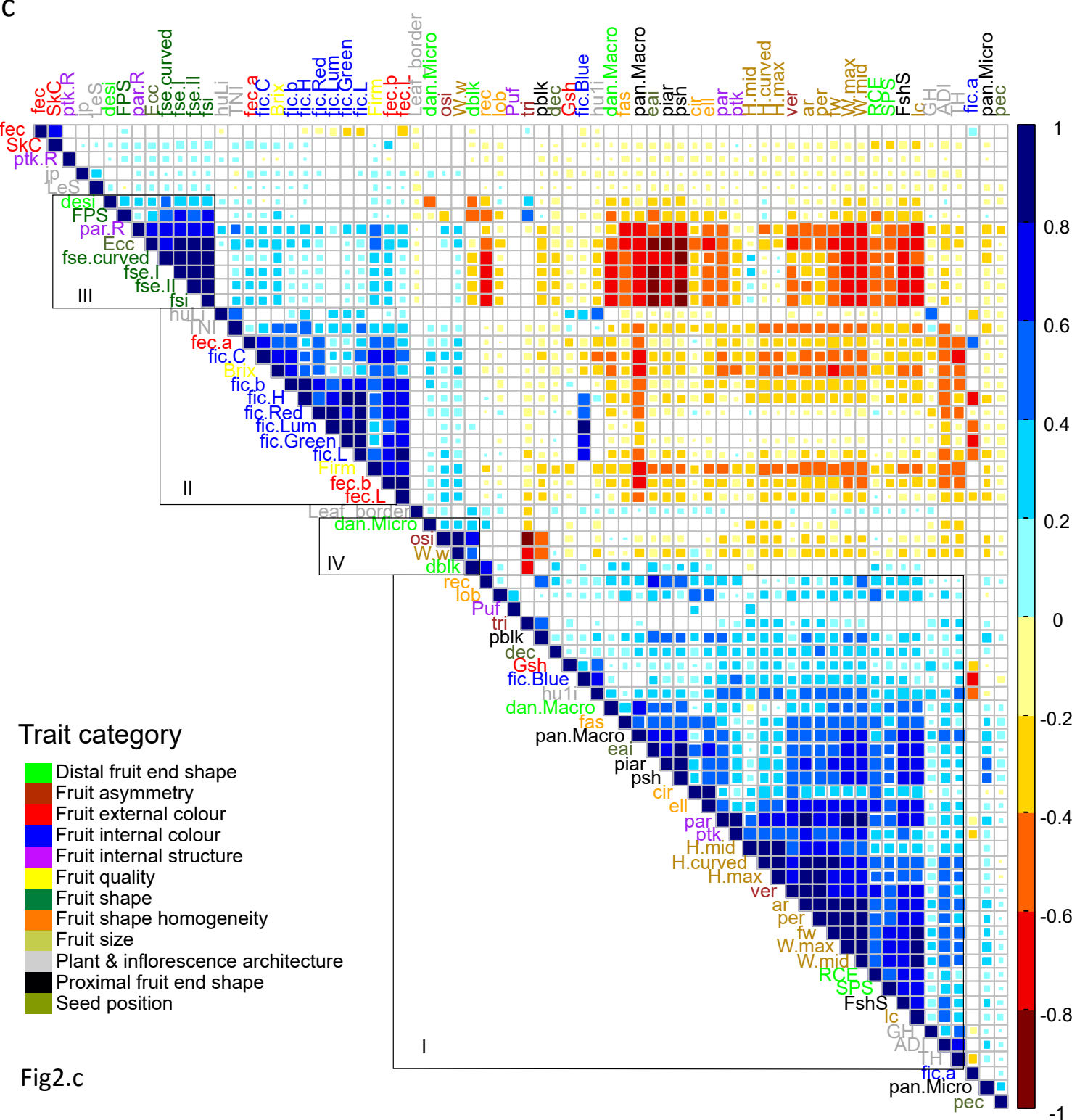

d

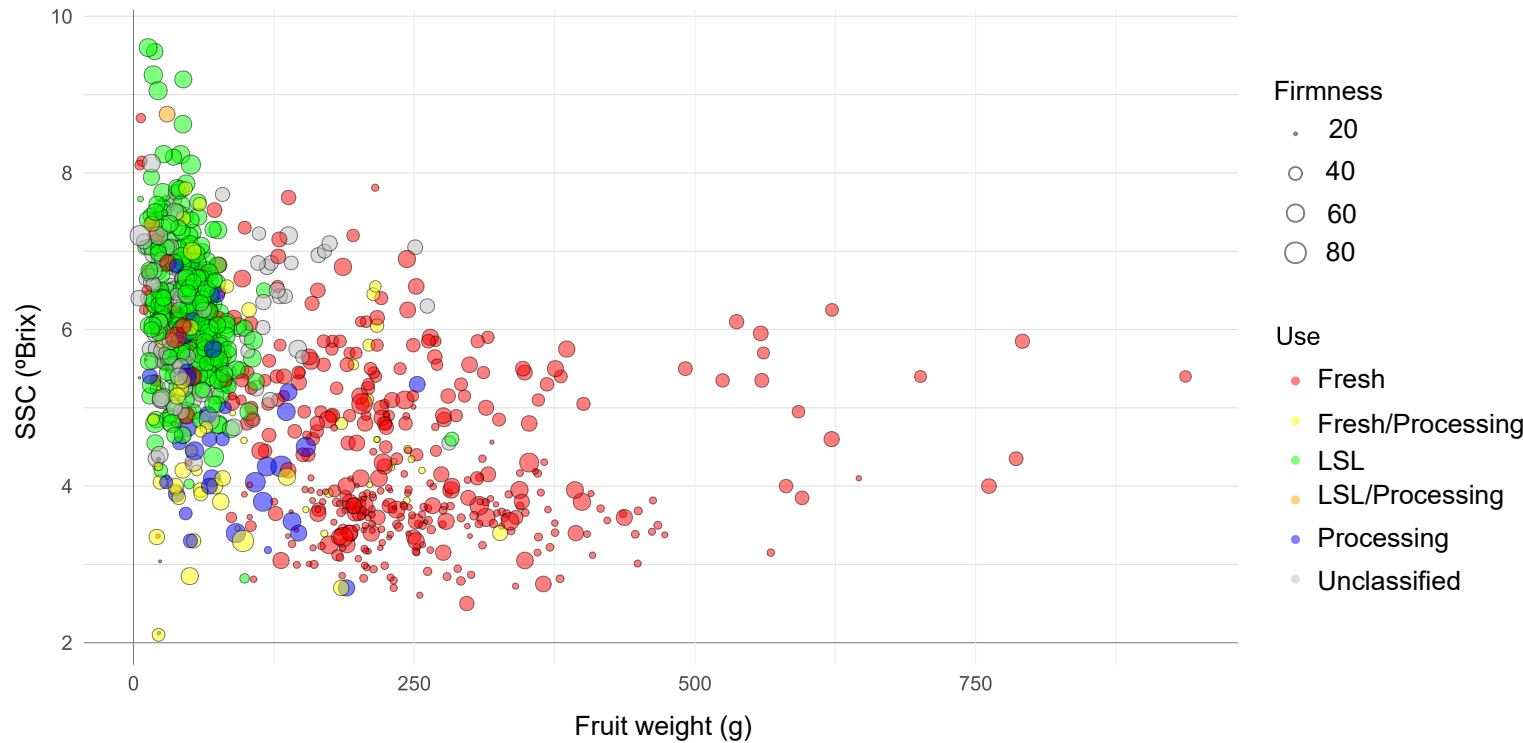

Fig. 2d

a

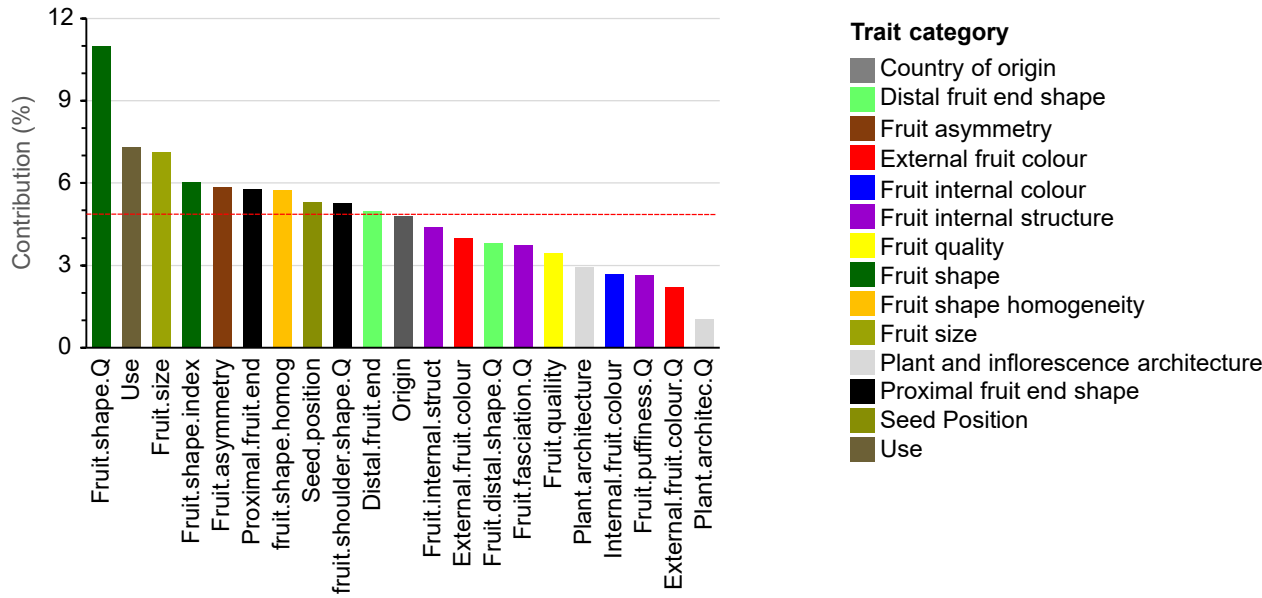

Fig. 3a

b

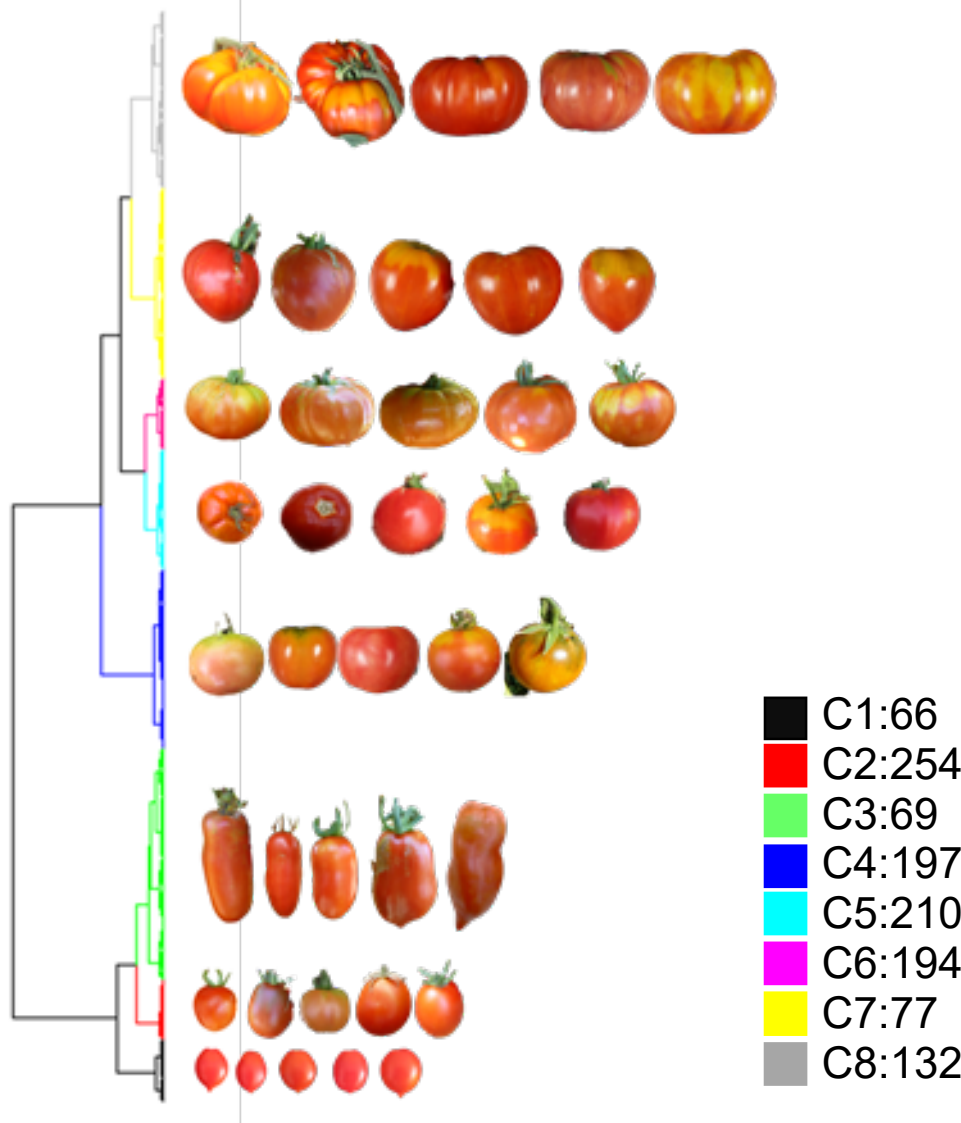

Fig. 3b

C

C8  
C7  
C6  
C5  
C4  
C3  
C2  
C1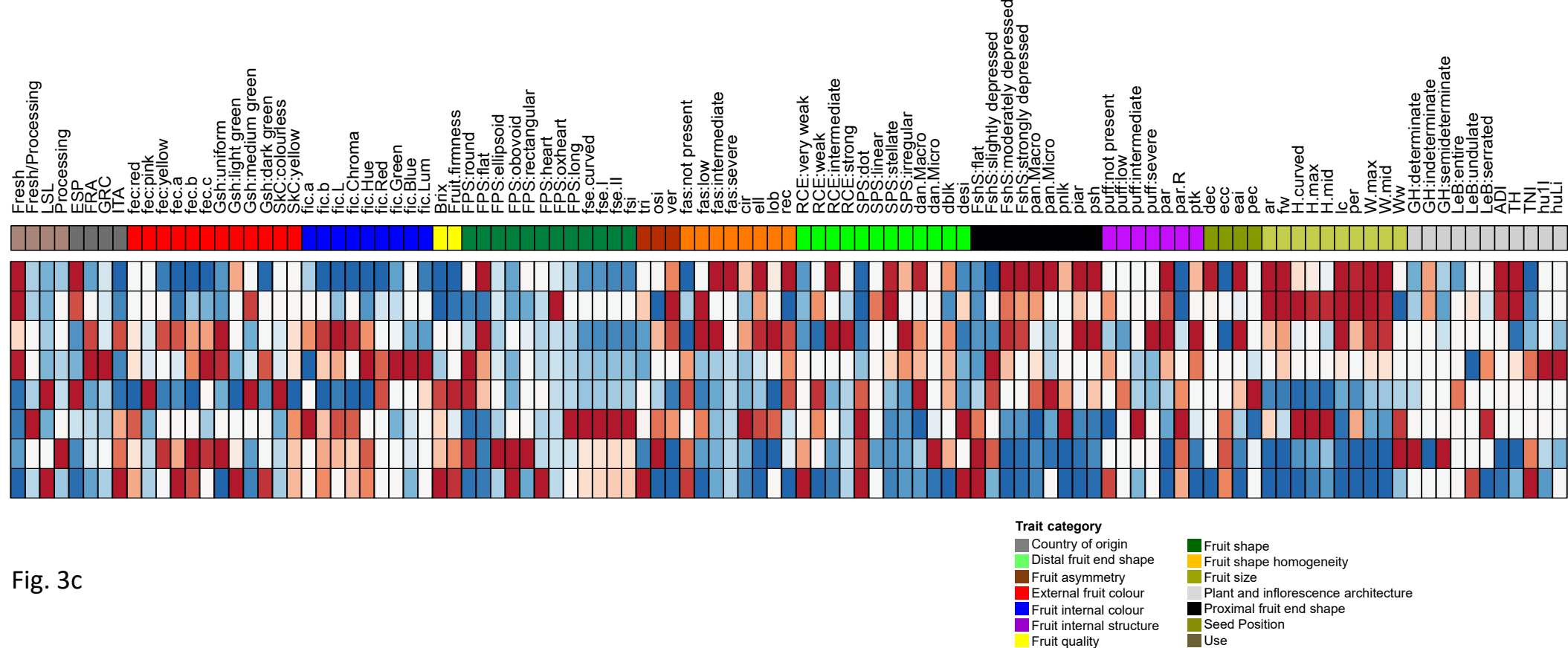

Fig. 3c

d

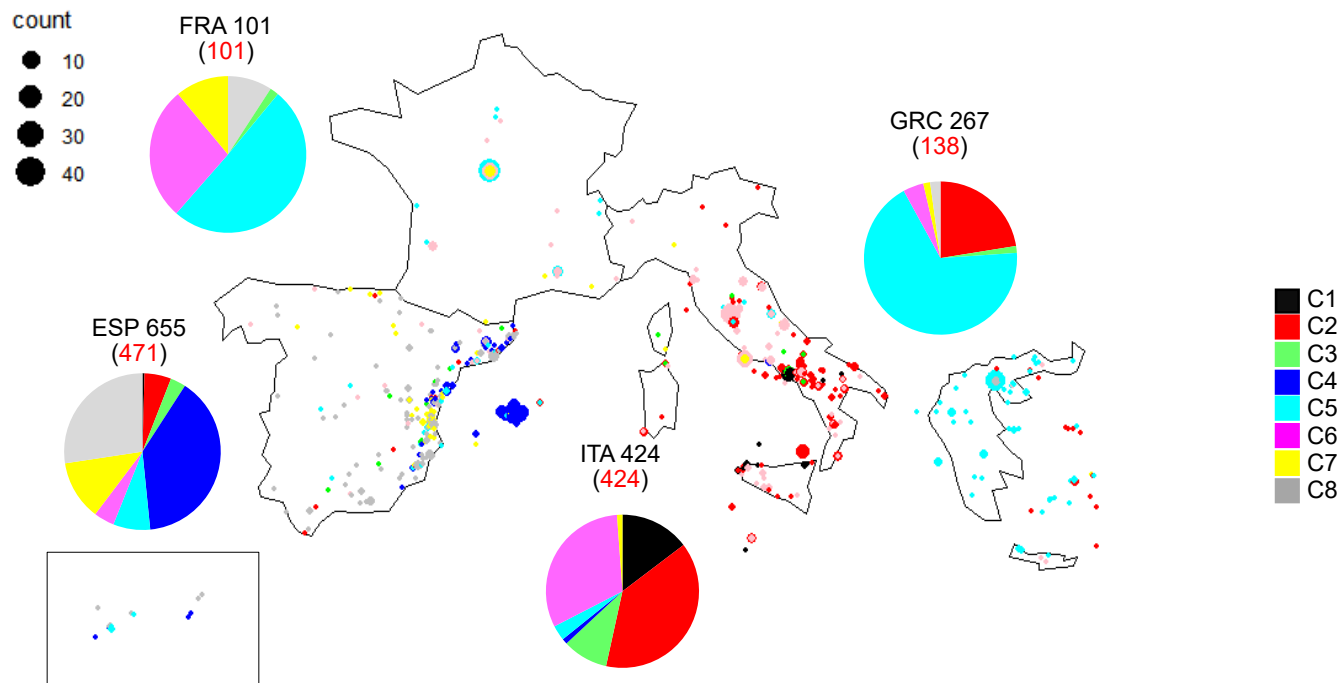

Fig. 3d

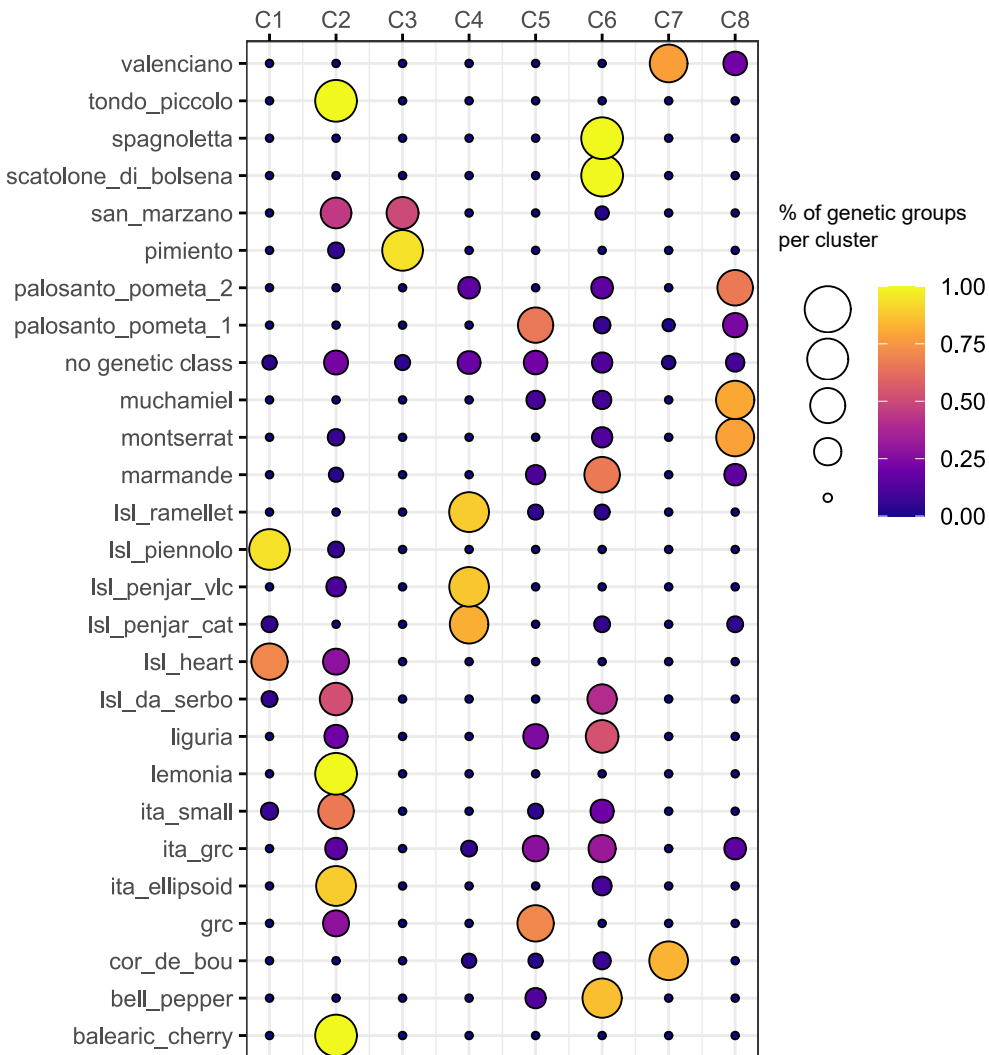

Fig. 3e

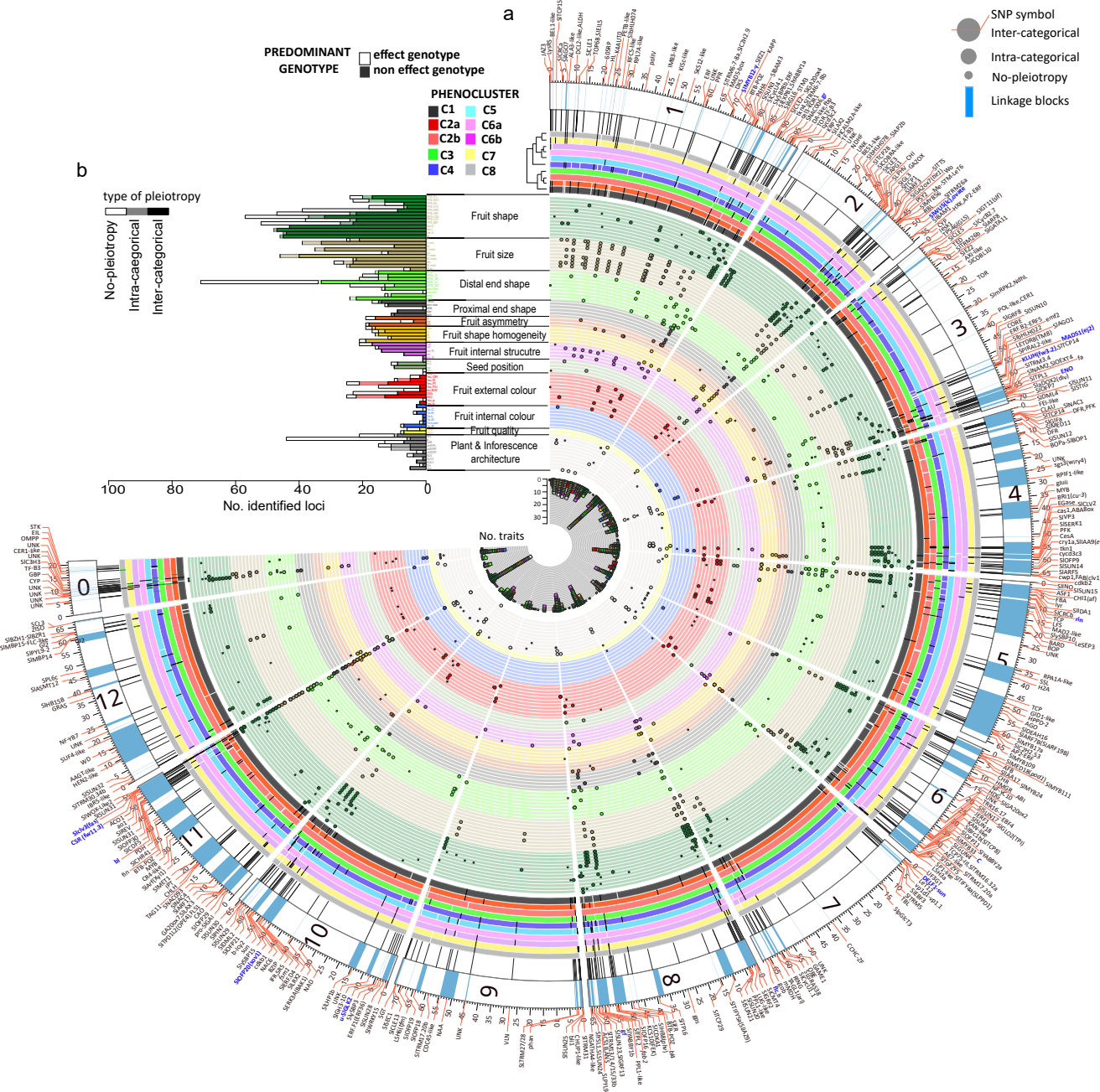

Supplement: Web_Material_uhac112 [file web_material_uhac112.zip › Apendix_main_fig.pdf]
